# Supplementary material for: A unified mechanism for mitochondrial damage sensing in PINK1-Parkin–mediated mitophagy
Source: EMBO J. 2025 Nov 20;45(1):64–105. doi: 10.1038/s44318-025-00604-z (PMC12759083; doi:10.1038/s44318-025-00604-z)
Supplement: Supplementary file 1 — Appendix [file 44318_2025_604_MOESM1_ESM.pdf]

Appendix

A Unified Mechanism for Mitochondrial Damage Sensing in PINK1-Parkin–Mediated Mitophagy

Julia A. Thayer[1], Jennifer D. Petersen[1,7], Xiaoping Huang[1,7], Luiza M. Gruel Budet[1,2], James Hawrot[2,3], Daniel M. Ramos[4], Shiori Sekine[5], Yan Li[6], Michael E. Ward[3], Derek P. Narendra[1]*

Appendix Figure S1: 2 – 4

Appendix Figure S2: 5 – 6

Appendix Figure S3: 7 – 9

Appendix Figure S4: 10 – 11

Appendix Figure S5: 12 – 13

Appendix Figure S6: 14 – 15

Appendix Figure S7: 16 – 17

Appendix Figure S1

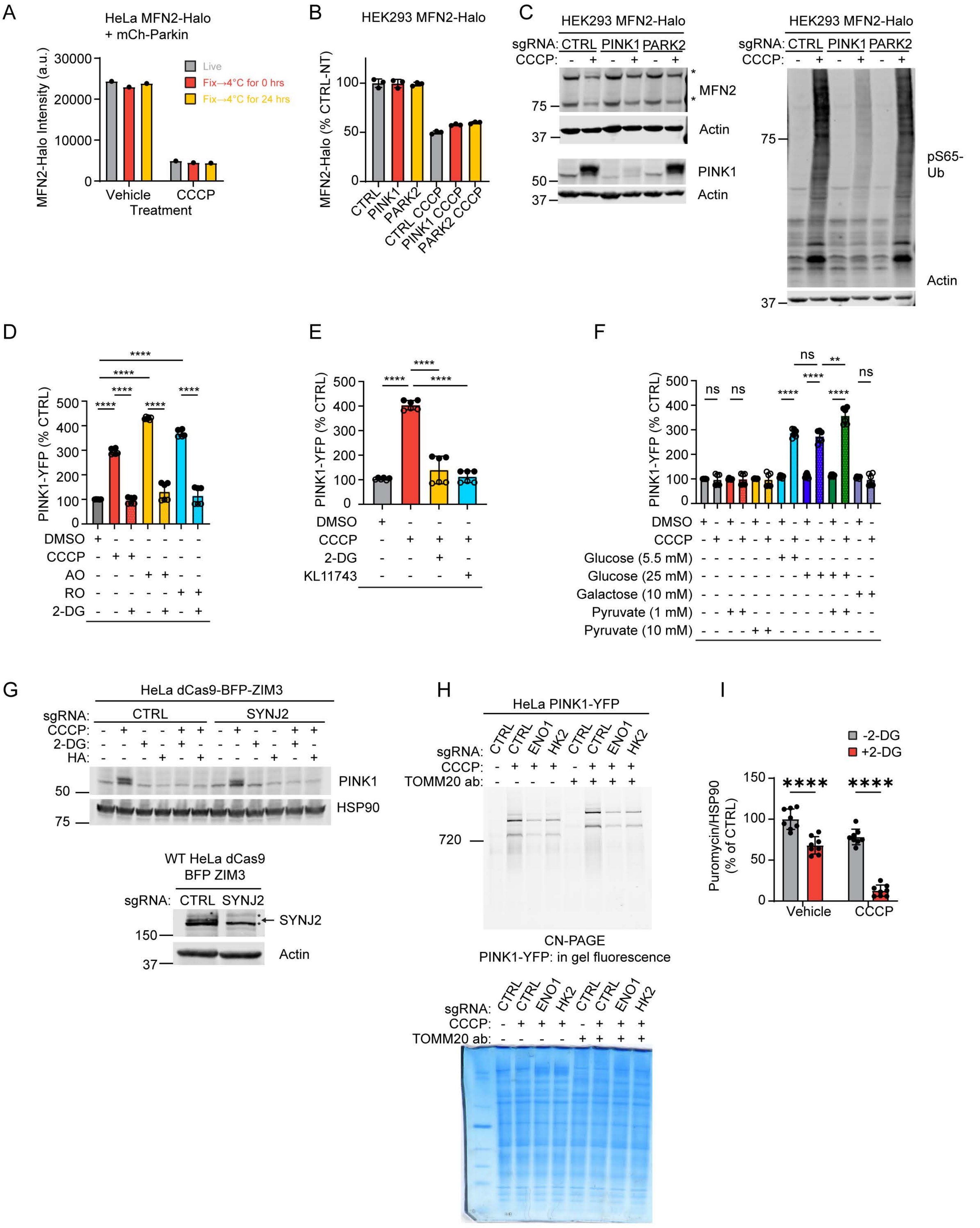

Appendix Figure S1. MFN2-HaLo reporter validation and glycolysis is required for PINK1-YFP stabilization following OXPHOS impairment.

(A) Flow cytometry of HeLa$^{MFN2\text{-}Halo+mCh\text{-}Parkin}$ cells +/- 10 µM CCCP 4hrs with or without fixation, demonstrating reporter retains signal after fixation. N = 1 independent experiment.

(B) Flow cytometry of HEK293$^{MFN2\text{-}Halo}$ cells with the indicated sgRNA +/- 10 µM CCCP O/N, illustrating MFN2-Halo degradation. Error bars mean +/- SD. N = 3 replicates on 1 occasion.

(C) Representative immunoblots of HEK293$^{MFN2\text{-}Halo}$ cells with the indicated sgRNA illustrating endogenously tagged and untagged MFN2 alleles (top and bottom band, respectively) +/- 10 µM CCCP 4 hrs. * denotes ubiquitinated MFN2 band. N = 2 replicates on 1 occasion.

(D) Flow cytometry measurements in HeLa$^{PINK\text{-}YFP}$ cells treated with 10 µM CCCP, and 8 µg/mL antimycin + 10 µg/mL oligomycin, 1 µM rotenone + 10 µg/mL oligomycin, and/or 10 mM 2-DG, for 4 hrs., illustrating PINK1-YFP levels. **** = p ≤ 0.0001 (exact p-values - DMSO vs CCCP, p = 6.9e-07; CCCP vs CCCP+2DG, p = 3.7e-10; DMSO vs AO, p = 1.1e-09; AO vs AO+2DG, p = 2.9e-05; DMSO vs RO, p = 3.1e-07; RO vs RO+2DG, 1.7e-05) by Brown-Forsythe and Welch ANOVA test with Dunnett's T3 multiple comparisons test. Error bars mean +/- SD. N = 6 independent experiments from 2 separate occasions (separate occasions denoted by open or closed circles).

(E) Flow cytometry measurements in HeLa$^{PINK1\text{-}YFP}$ cells treated with 10 µM CCCP, 10 µM KL11743 and/or 10 mM 2-DG, for 4 hrs, illustrating PINK1-YFP levels. **** = p ≤ 0.0001 (exact p-values - DMSO vs CCCP, p = 6.6e-08; CCCP vs CCCP+2DG, p = 1e-04; CCCP vs CCCP+KL11745, p = 1.2e-09) by Brown-Forsythe and Welch ANOVA test with Dunnett's T3 multiple comparisons test. Error bars mean +/- SD. N = 6 independent experiments from 2 separate occasions (separate occasions denoted by open or closed circles).

(F) Flow cytometry measurements in HeLa$^{PINK1\text{-}YFP}$ cells treated with 10 µM CCCP +/- in glucose and pyruvate-free DMEM supplemented with glucose, galactose, and/or pyruvate at the indicated concentrations for 4 hrs, illustrating PINK1-YFP levels. ns (from left to right) = p = 0.9998, >0.9999, 0.8650, 0.9307, ** = p= 0.0051, **** = $p \leq 0.0001$ (exact p-values from left to right, p = 1.6e-06, 2.8e-05, 6.2e-05) by Brown-Forsythe and Welch ANOVA test with Dunnett's T3 multiple comparisons test. Error bars mean +/- SD. N = 6 independent experiments from 2 separate occasions (separate occasions denoted by open or closed circles).

(G) Representative immunoblots of HeLa$^{dCas9\text{-}BFP\text{-}ZIM3}$ cells with indicated sgRNA treated with 10 µM CCCP, 10 µM HA, and/or 10 mM 2-DG 4 hrs, illustrating PINK1 levels are unchanged in the presence of SYNJ2 sgRNA. * denotes non-specific bands, arrow denotes position of SYNJ2 band. N = 2 independent experiments.

(H) CN-PAGE separated PINK1-YFP complexes visualized by in gel fluorescence as in (Fig. 5J) (top) and total protein measured via SimplyBlue SafeStain (bottom). HeLa$^{PINK1\text{-}YFP}$ cells were cultured with indicated sgRNA for at least 7 days before CN-PAGE sample collection. N = 3 replicates from at least 2 transductions.

(I) Quantification of immunoblots represented in figure 2G. **** = $p \leq 0.0001$ (exact p-values, left p = 1.5e-06; right p = 4.1e-13) by two way ANOVA with Šídák's multiple comparisons test. Error bars mean +/- SD. N = 3 independent experiments.

Appendix Figure S2

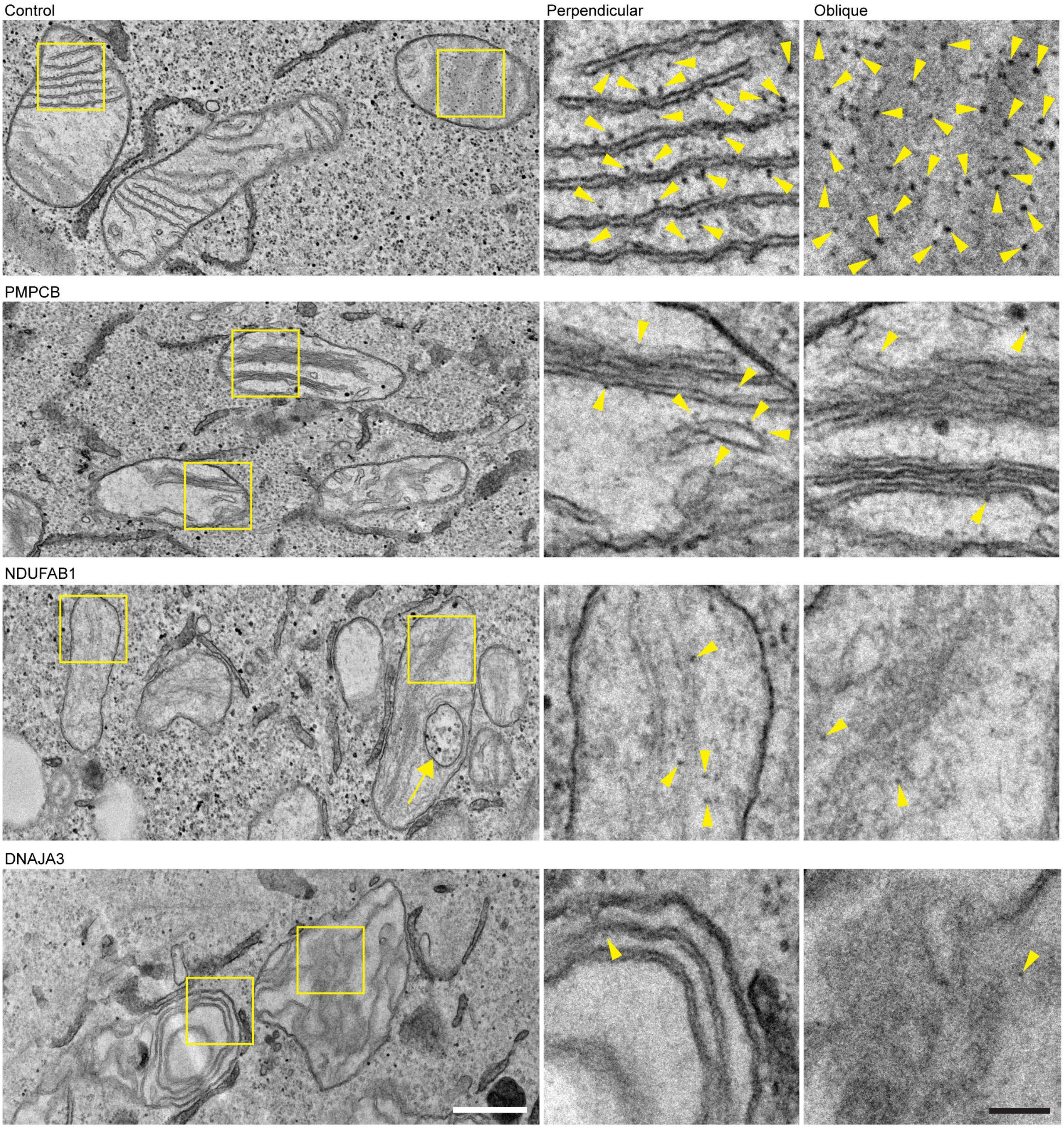

Appendix Figure S2. Cristae membranes may lose F_1F_O-ATP synthase by TEM following mitochondrial perturbations that activate PINK1-Parkin.

(A) EM shows loss of cristae-associated punctate densities that may correspond to the F_1F_O-ATP synthase in $HeLa^{dCas9\text{-}BFP\text{-}ZIM3}$ cells transduced with sgRNA targeting PMPCB, NDUFAB1, and DNAJA3. In control cells (top), numerous punctate structures decorate cristae. Yellow boxed areas shown to the right with yellow arrowheads indicate the abundance of presumed F_1F_O-ATPase synthase visible along cristae cut perpendicularly in thin sections and when cristae are sectioned in an oblique orientation. Possible F_1F_O-ATP synthase particles are observed along cristae in mitochondria of cells expressing sgRNA for PMPCB and NDUFAB1, but at a reduced frequency. Possible F_1F_O-ATP synthase densities are virtually absent along cristae of mitochondria in cells transduced with sgRNA for DNAJA3. White scale bar = 500 nm; black scale bar = 100 nm.

Appendix Figure S3

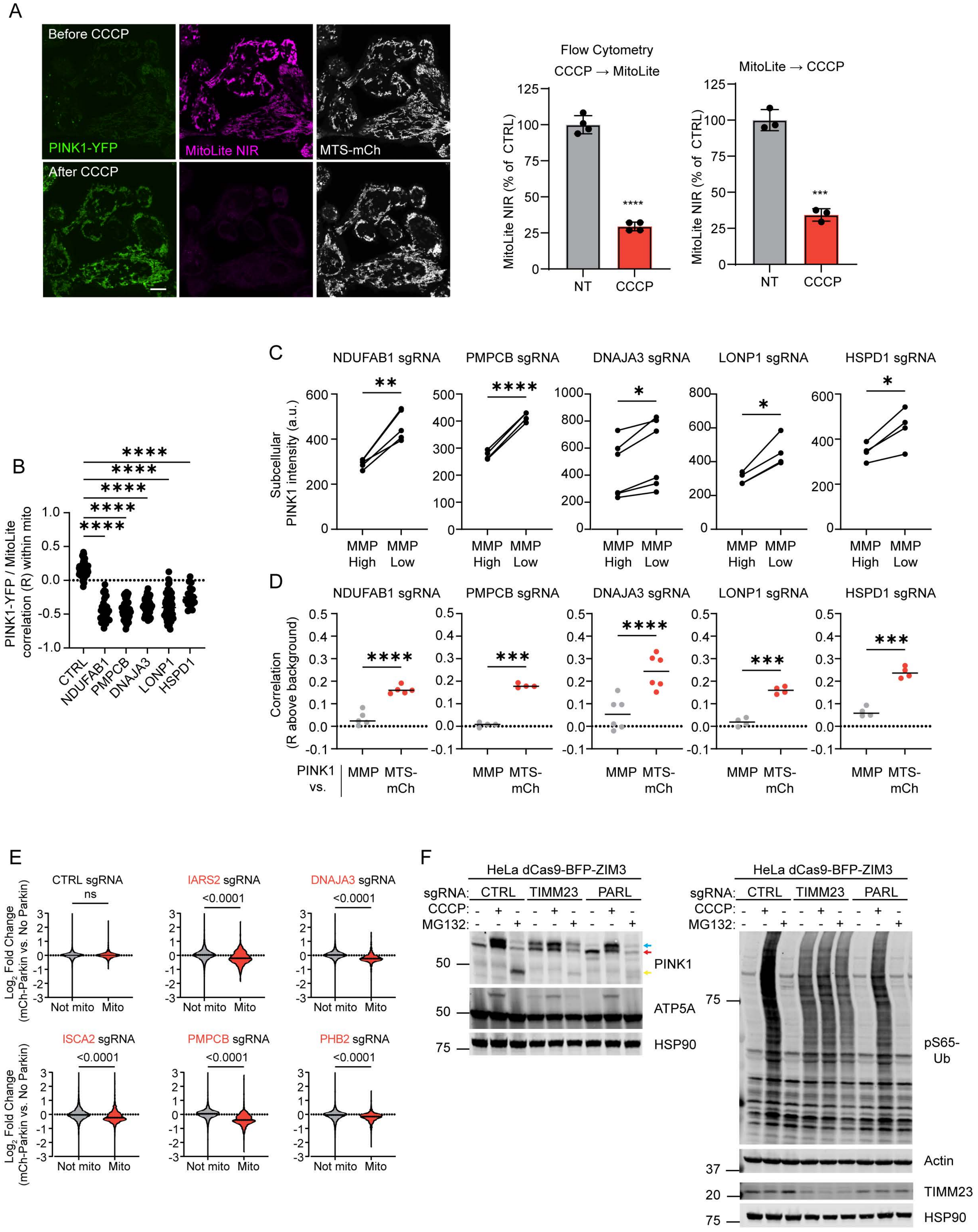

Appendix Figure S3. PINK1-YFP accumulation as a result of PINK1-Parkin activators negatively correlates with MPP.

(A) Left - Representative images of HeLa$^{PINK1\text{-}YFP+MTS\text{-}mCh}$ cells demonstrating loss of MMP after addition of 10 µM CCCP measured by MitoLite NIR dye. Cells were imaged live immediately before CCCP addition and again ~2 hrs following CCCP addition. Left graph - Quantification of flow cytometry data where CCCP was added to cells (4 hrs) followed by MitoLite, **** = p ≤ 0.0001 (exact p-value, p = 9.1e-07) by two-tailed unpaired t test, N = 4 replicates. Right graph - Quantification of flow cytometry data where MitoLite was added to cells followed by CCCP treatment (25 minutes) *** = p = 0.0002, by two-tailed unpaired t test, N = 3 replicates. Error bars mean +/- SD.

(B) Graph depicting the negative correlation between PINK1-YFP and MPP within mitochondria in HeLa $^{YFP\text{-}PINK+MTS\text{-}mCh}$ cells transduced with the indicated sgRNA. Each data point represents the correlation between the two channels for all pixels within a mitochondria mask in a single cell. These data are drawn from the same data set as figures 3F-G, 4I, 6H, 6J, EV5G. **** = p ≤ 0.0001 (exact p-values – CTRL vs NDUFAB1, p < 1e-15; CTRL vs PMPCB, p < 1e-15; CTRL vs DNAJA3, p < 1e-15; CTRL vs LONP1, p < 1e-15; CTRL vs HSPD1, p = 2.5e-10) by Brown-Forsythe and Welch Anova tests.

(C) HeLa $^{YFP\text{-}PINK+MTS\text{-}mCh}$ cells transduced with the indicated sgRNA demonstrating that PINK1 intensity is higher in cells with low MMP. Each data point represents the average value from all cells within a replicate. These data are drawn from the same data set as figures 3F-G, 4I, 6H, 6J, EV5G. DNAJA3 * = p = 0.0101, LONP1 * = p = 0.0131, HSPD1 * = p = 0.0226, NDUFAB1 ** = p = 0.0023, PMPCB **** = p ≤ 0.0001 (exact p-value, p = 4.27e-05) by two-tailed paired t test.

(D) HeLa $^{YFP\text{-}PINK+MTS\text{-}mCh}$ cells transduced with the indicated sgRNA demonstrating that PINK1-YFP accumulation correlates with MTS-mCh not MPP. Each data point represents the average

value from all cells within a replicate. These data are drawn from the same data set as figures 3F-G, 4I, 6H, 6J, EV5G PMPCB *** = p = 0.0004, LONP1 *** = p = 0.0009, HSPD1 *** = p = 0.0006, **** = p ≤ 0.0001 (exact p-values - NDUFAB1 p = 7.74e10-05; DNAJA3 p = 1e-05) by two-tailed paired t test.

(E) LFQ performed as described in (Fig. 3H) comparing knockdowns in HeLa cells with mCh-Parkin vs. HeLa cells with no Parkin. HeLa cells without Parkin are from the same data that appears in (Fig. 3H). Statistics CTRL = ns = p = 0.4941, **** = p ≤ 0.0001 (exact p-values – IARS2, p < 1e-15; DNAJA3, p < 1e-15; ISCA2, p < 1e-15; PMPCB, p < 1e-15; PHB2, p < 1e-15) by two-tailed Mann Whitney test.

(F) Representative immunoblots of HeLa$^{dCas9\text{-}BFP\text{-}ZIM3}$ cells transduced with indicated sgRNAs and treated with 10 µM CCCP or 50 µM MG132 for 4 hrs, illustrating PINK1 stabilization and activation. N = 4 replicates from 2 independent transductions. Arrows: blue – FL PINK1, red – Δ-MTS PINK1, yellow – 52-kDa PINK1.

Appendix Figure S4

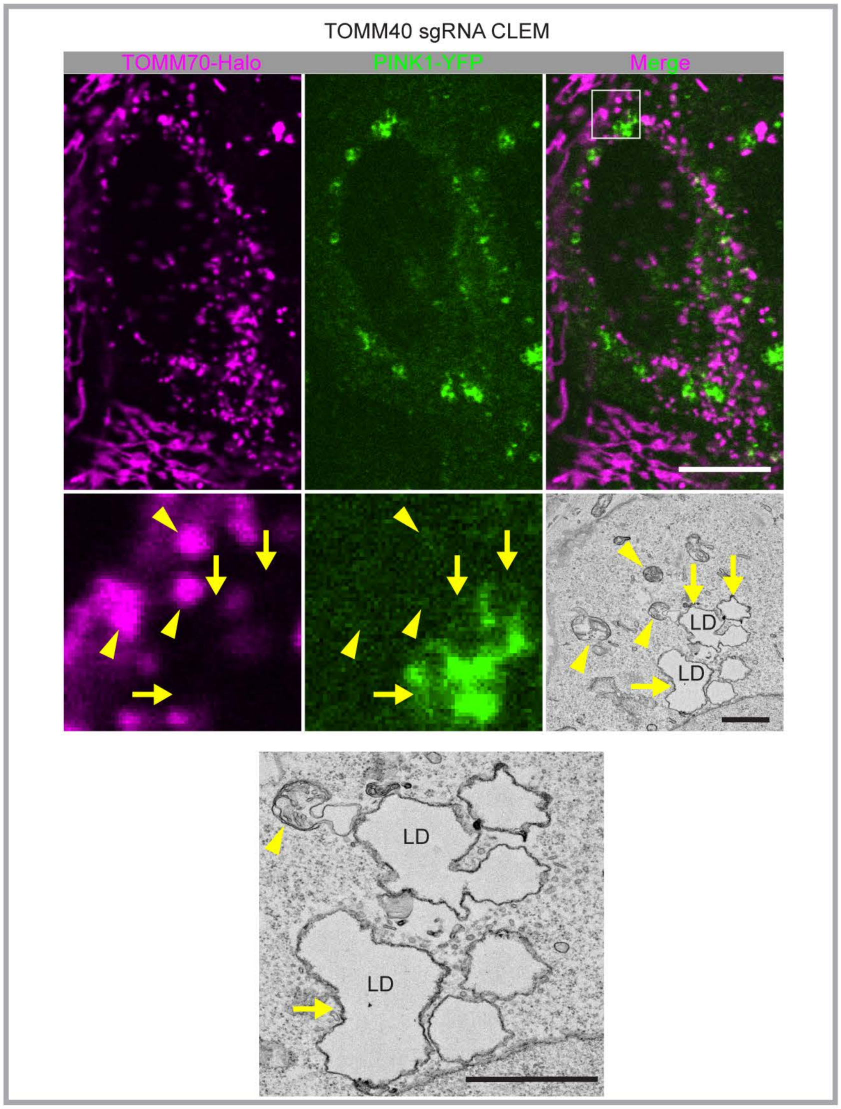

Appendix Figure S4. TOMM70 labels mitochondria in the absence of TOMM40.

(A) CLEM of HeLa$^{PINK1\text{-}YFP}$ cells endogenously tagged with TOMM70-Halo and transduced with sgRNA targeting TOMM40. Fluorescence confocal microscopy shows that in cells with sgRNA-mediated knockdown of TOMM40, the mitochondrial marker, TOMM70-Halo and PINK1-YFP were not colocalized. The same cell examined at the EM level and aligned with the confocal image showed TOMM70-Halo label colocalized with small round mitochondria while the PINK1-YFP signal localized to a cluster of lipid droplets (LD). Yellow arrowheads indicate Halo-labeled mitochondria. Yellow arrows indicate site of PINK1-YFP accumulation around lipid droplets. White scale bars = 10 µm, black scale bars = 1 µm.

Appendix Figure S5

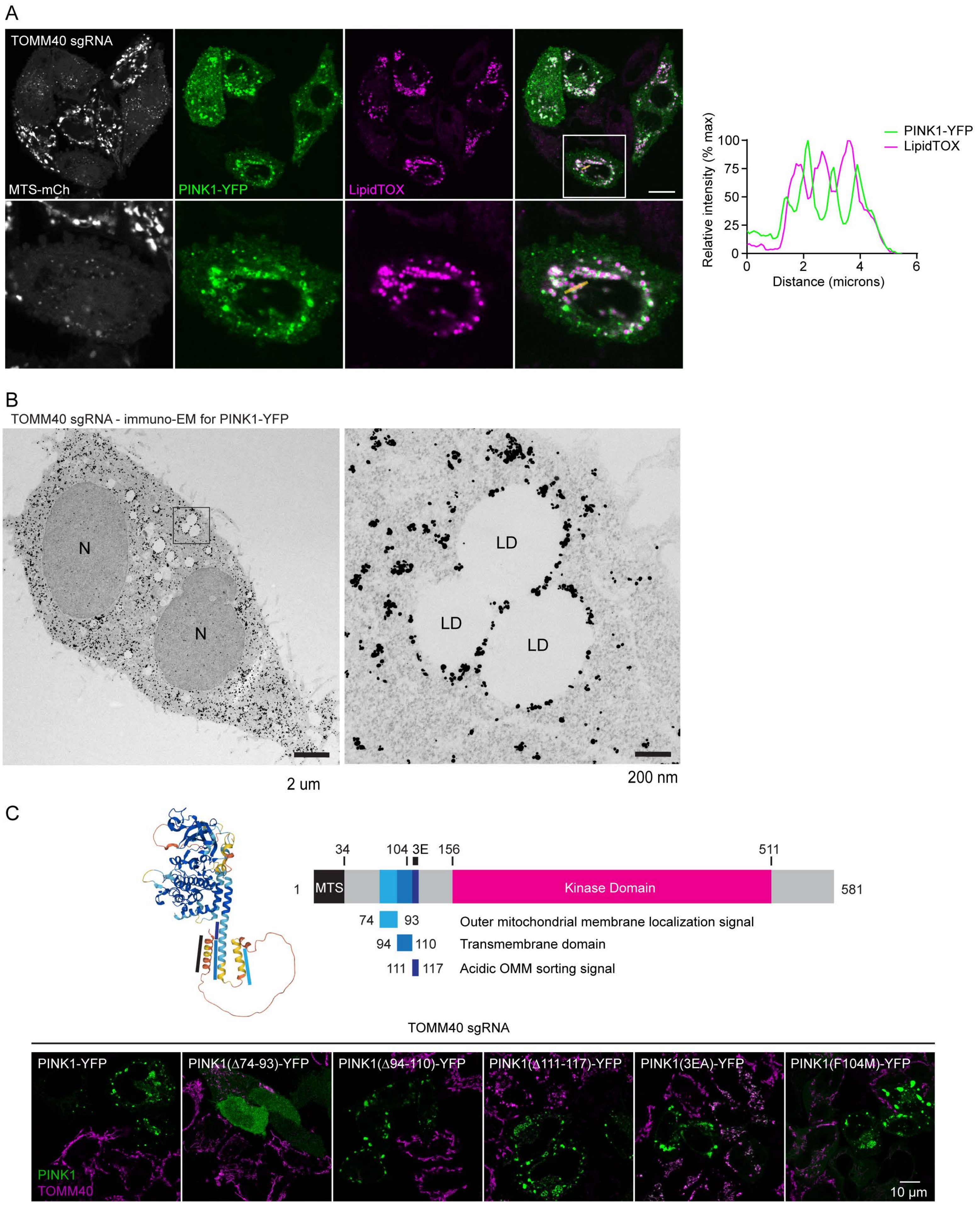

Appendix Figure S5. PINK1-YFP accumulates on lipid droplets in the presence of TOMM40 KD.

(A) Representative images of HeLa$^{PINK1\text{-}YFP+MTS\text{-}mCh}$ cells transduced with TOMM40 sgRNA, demonstrating accumulation of PINK1-YFP on lipid droplets (LipidTOX). White box outlines zoomed in region on bottom row, Graph – line scan of yellow line in bottom right image. Scale bar = 10 µm.

(B) Representative TEM images of HeLa$^{PINK1\text{-}YFP}$ cells transduced with TOMM40 sgRNA and stained with GFP antibody/immunogold labeling, demonstrating GFP accumulation on the lipid droplets (LD). Right image (Scale bar = 200 nm) is the zoomed in box image from left image (Scale bar = 2 µm). N = nucleus.

(C) Top - Structure of PINK1 (left) and domain structure of PINK1(right) with deletions utilized in the bottom images labeled. Bottom – Representative images of HeLa cells transiently transfected with the indicated PINK1-YFP constructs and imaged to visualize PINK1-YFP localization to mitochondria (TOMM40 staining). Scale bar = 10 µm.

Appendix Figure S6

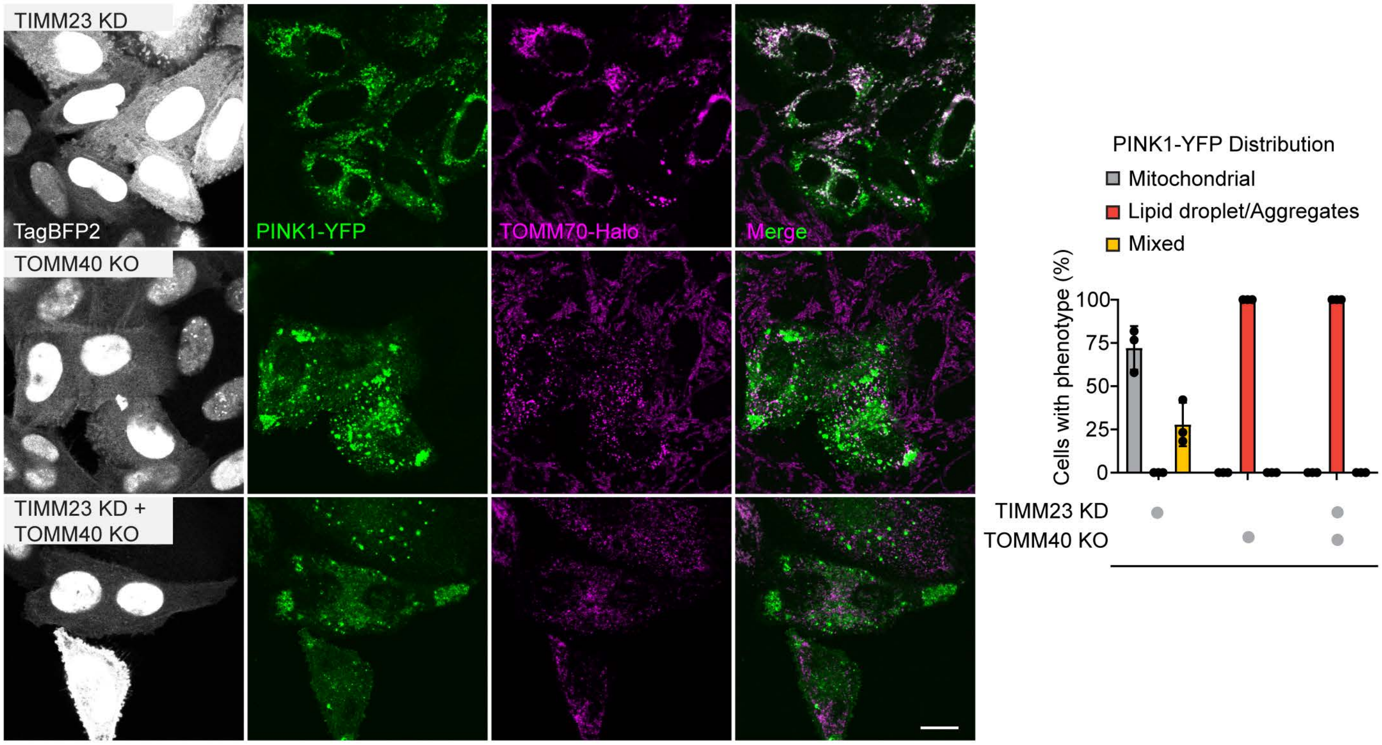

Appendix Figure S6. Disruption of both TOMM40 and TIMM23 phenocopies TOMM40 KO.

Representative images of HeLa $^{PINK1\text{-}YFP\ +\ TOMM70\text{-}Halo}$ cells with TOMM40 KO via nucleofection, TIMM23 KD via sgRNA, or both demonstrating that loss of TOMM40 and TIMM23 phenocopies TOMM40 KO/KD. Cells were fixed 8 days post KO/7 days post KD. N = 3 replicates from 1 nucleofection/transduction. 141 cells were counted in total. Error bars mean +/- SD.

Appendix Figure S7

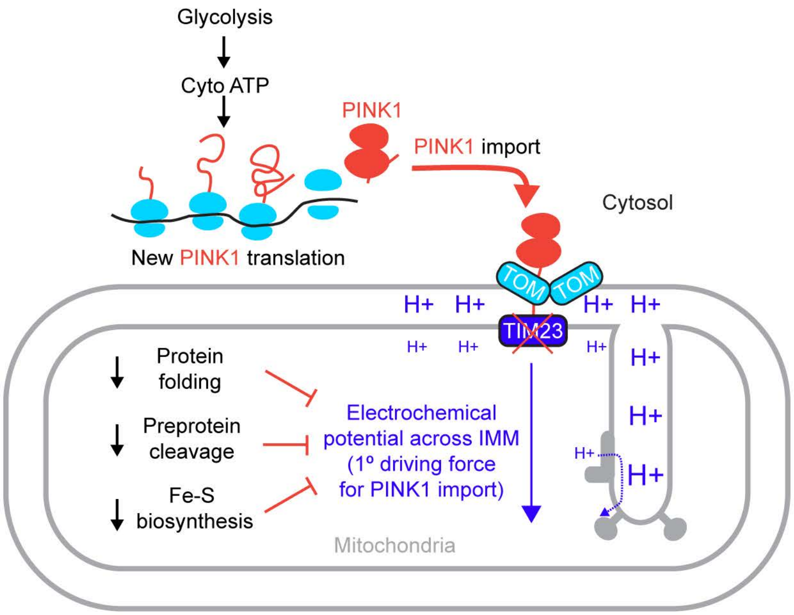

Damage-sensing on-off switch for PINK1-Parkin mitophagy

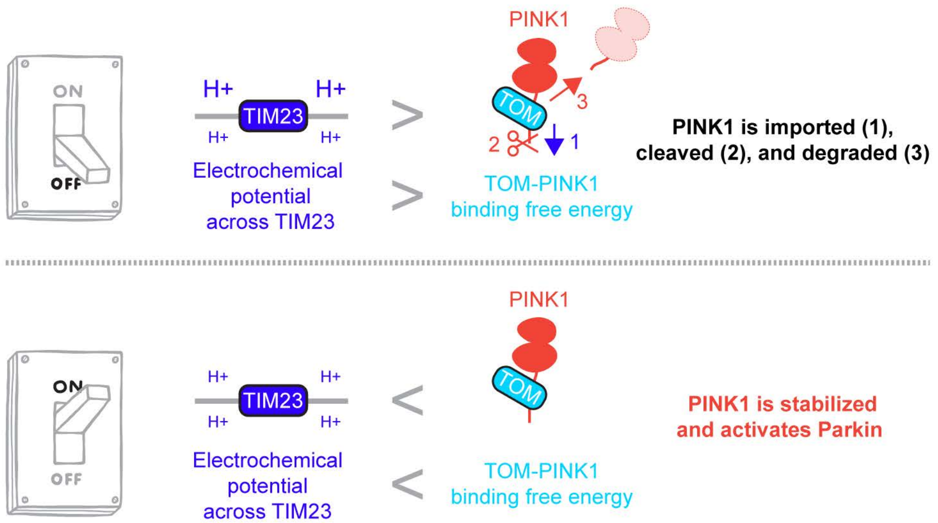

Appendix Figure S7. Schematic summarizing that the electrochemical potential across the IMM is the driving force of PINK1 import.

Top – Depiction of the requirement of glycolysis for PINK1 translation and block of PINK1 import in the absence of the TIM23 translocase as well as modifiers that disrupt the mitochondrial membrane potential (MPP) resulting in import block.

Bottom – Illustration demonstrating the damage-sensing on-off switch for PINK1-Parkin mitophagy. The switch is in the off position when MMP is stable allowing PINK1 import, cleavage and degradation. The switch is turned on when the electrochemical potential across TIM23 is disrupted resulting in the stabilization of PINK1 and activation of Parkin.
