# Supplementary figures and images for: A unified mechanism for mitochondrial damage sensing in PINK1-Parkin–mediated mitophagy (part 1 of 2)
Source: EMBO J. 2025 Nov 20;45(1):64–105. doi: 10.1038/s44318-025-00604-z (PMC12759083; doi:10.1038/s44318-025-00604-z)

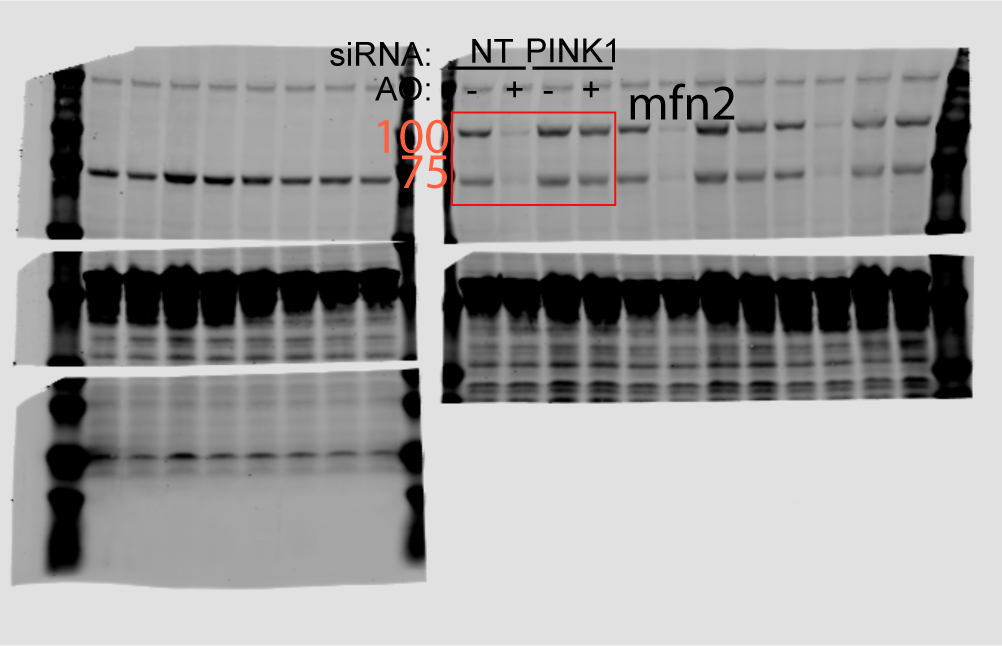

Supplement: Supplementary file 13 — Source data Fig. 1 [file 44318_2025_604_MOESM13_ESM.zip › Figure 1/1D/1D_western mfn2.tif]

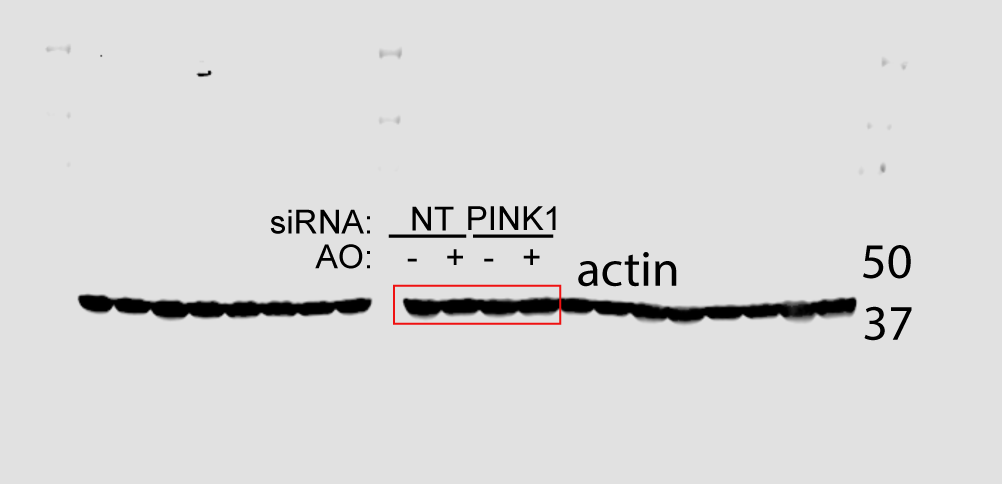

Supplement: Supplementary file 13 — Source data Fig. 1 [file 44318_2025_604_MOESM13_ESM.zip › Figure 1/1D/1D_western actin for pink1.tif]

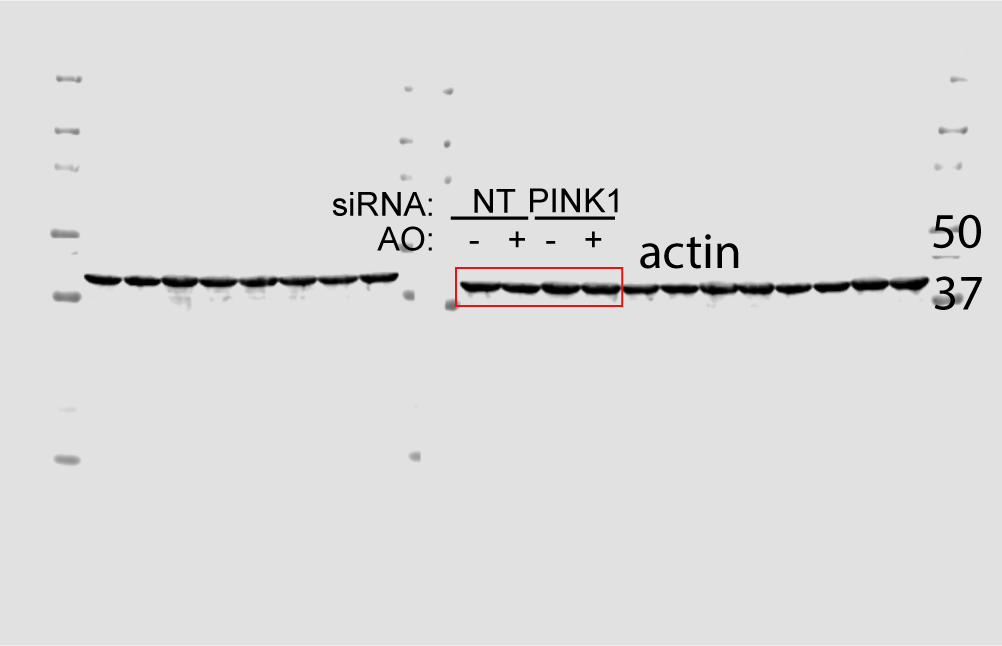

Supplement: Supplementary file 13 — Source data Fig. 1 [file 44318_2025_604_MOESM13_ESM.zip › Figure 1/1D/1D_western actin for mfn2_parkin.tif]

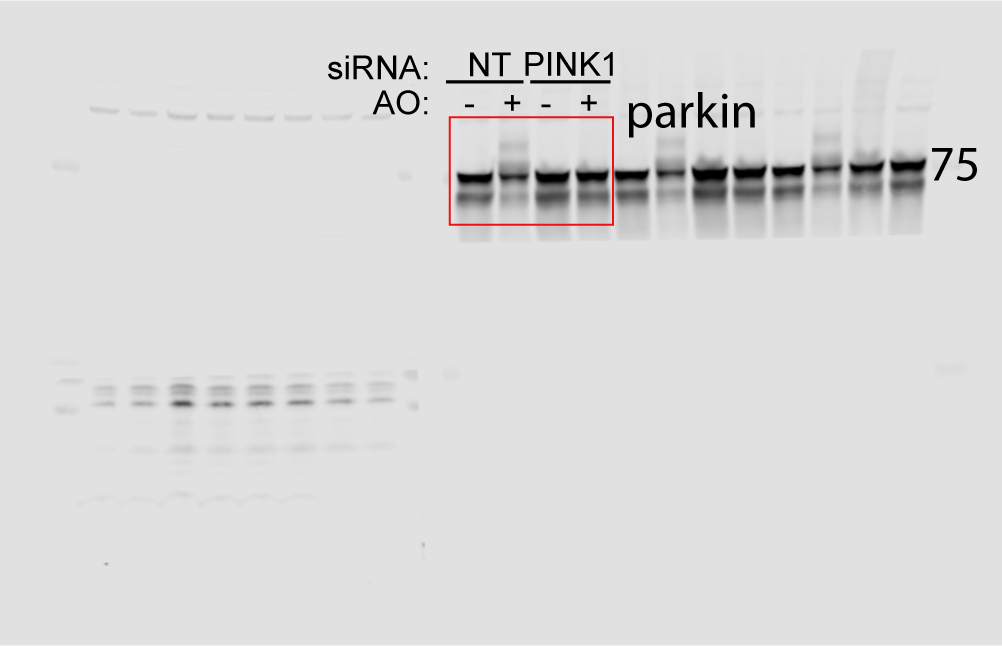

Supplement: Supplementary file 13 — Source data Fig. 1 [file 44318_2025_604_MOESM13_ESM.zip › Figure 1/1D/1D_western parkin.tif]

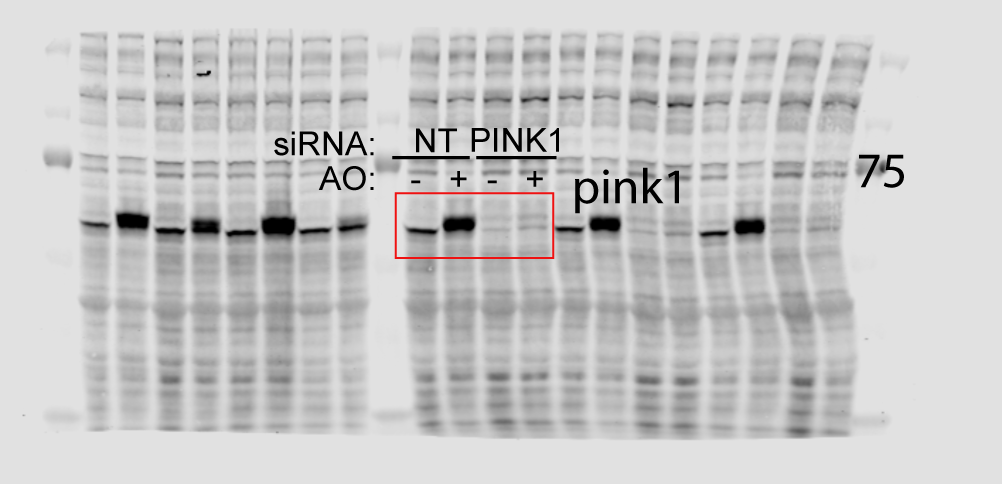

Supplement: Supplementary file 13 — Source data Fig. 1 [file 44318_2025_604_MOESM13_ESM.zip › Figure 1/1D/1D_western pink1.tif]

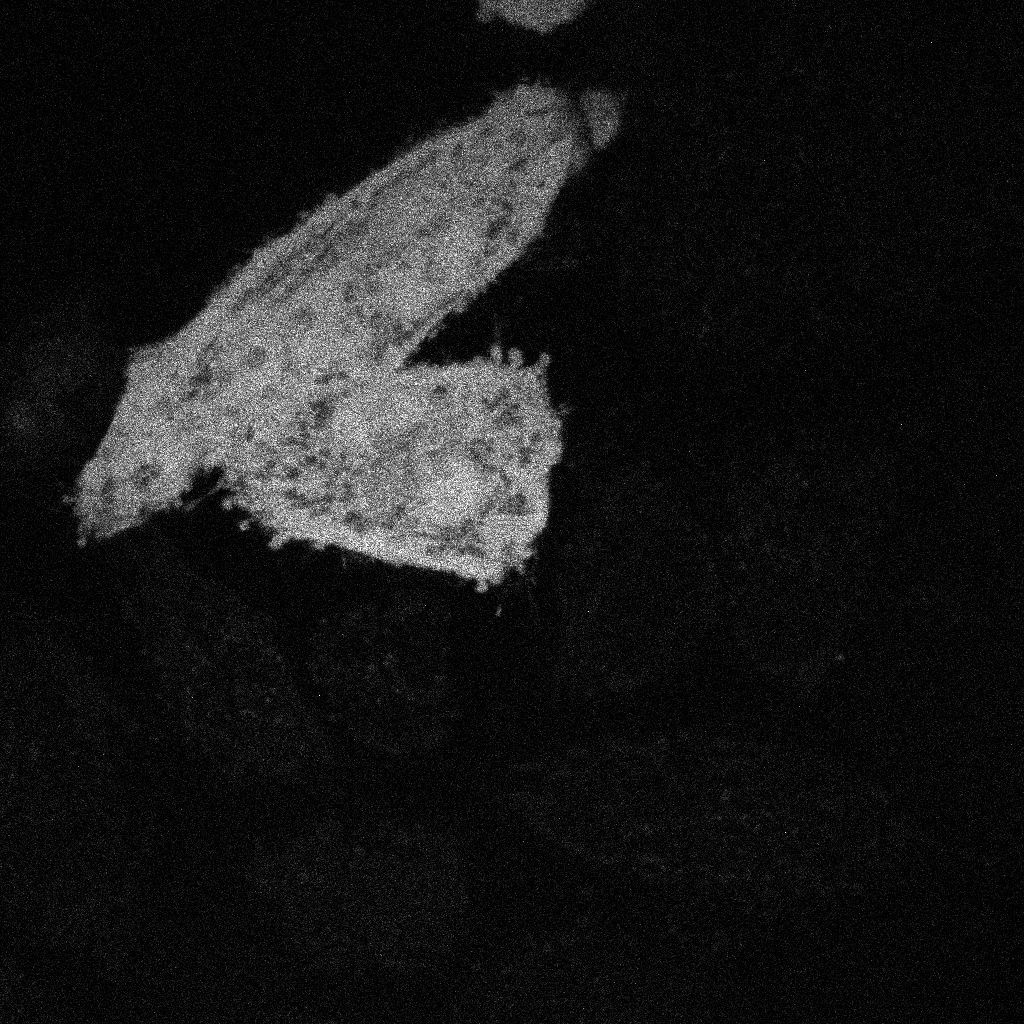

Supplement: Supplementary file 13 — Source data Fig. 1 [file 44318_2025_604_MOESM13_ESM.zip › Figure 1/1B/1B_image_channel1_TagBFP2.tif]

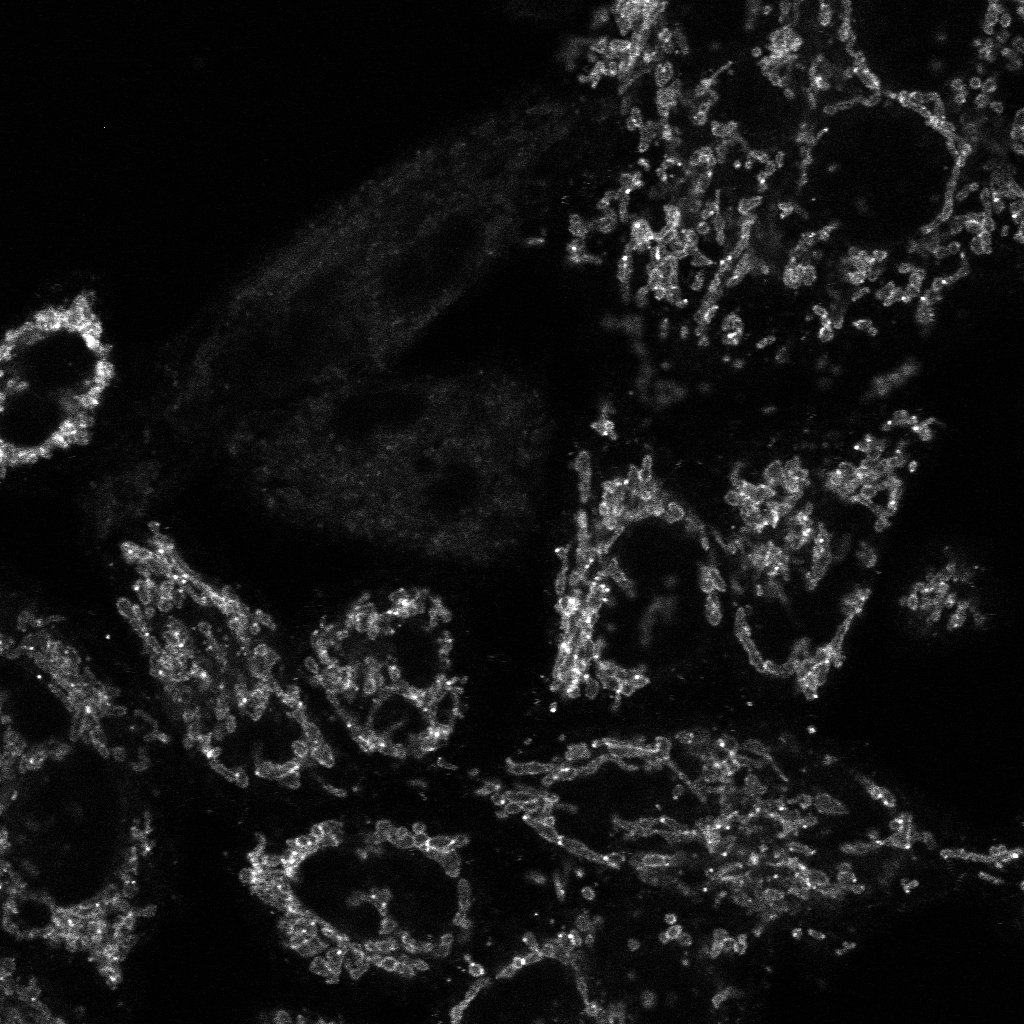

Supplement: Supplementary file 13 — Source data Fig. 1 [file 44318_2025_604_MOESM13_ESM.zip › Figure 1/1B/1B_image_channel3_MFN2HaloJF646.tif]

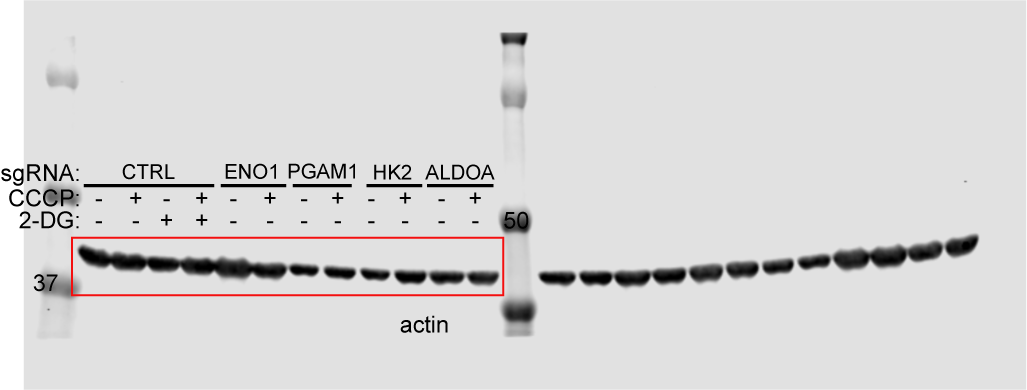

Supplement: Supplementary file 14 — Source data Fig. 2 [file 44318_2025_604_MOESM14_ESM.zip › Figure 2/2C/2C_western actin for pink1.tif]

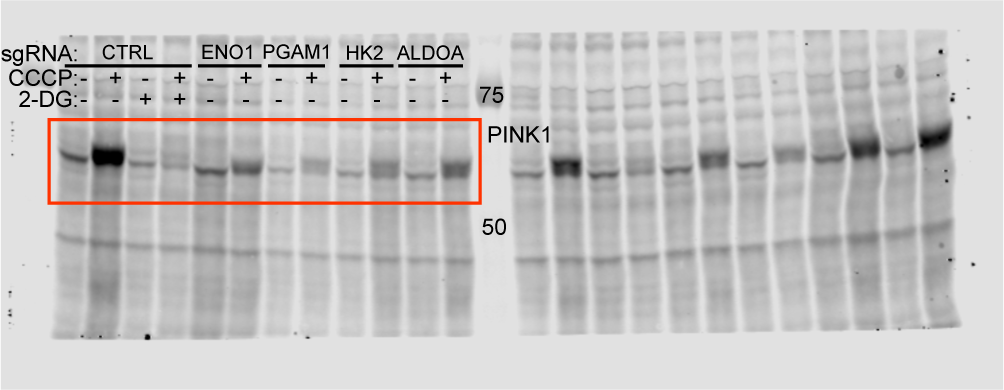

Supplement: Supplementary file 14 — Source data Fig. 2 [file 44318_2025_604_MOESM14_ESM.zip › Figure 2/2C/2C_western pink1.tif]

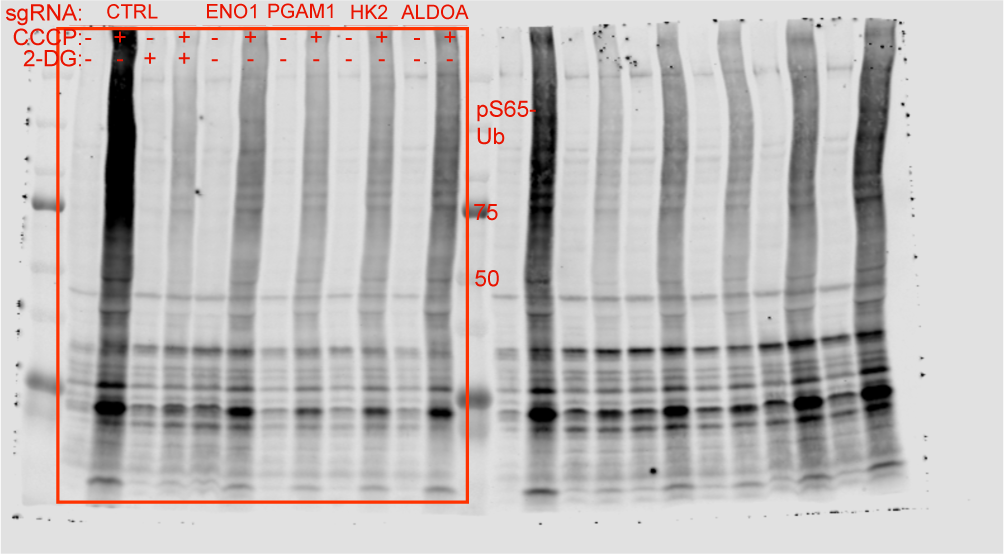

Supplement: Supplementary file 14 — Source data Fig. 2 [file 44318_2025_604_MOESM14_ESM.zip › Figure 2/2C/2C_western pS65-Ub.tif]

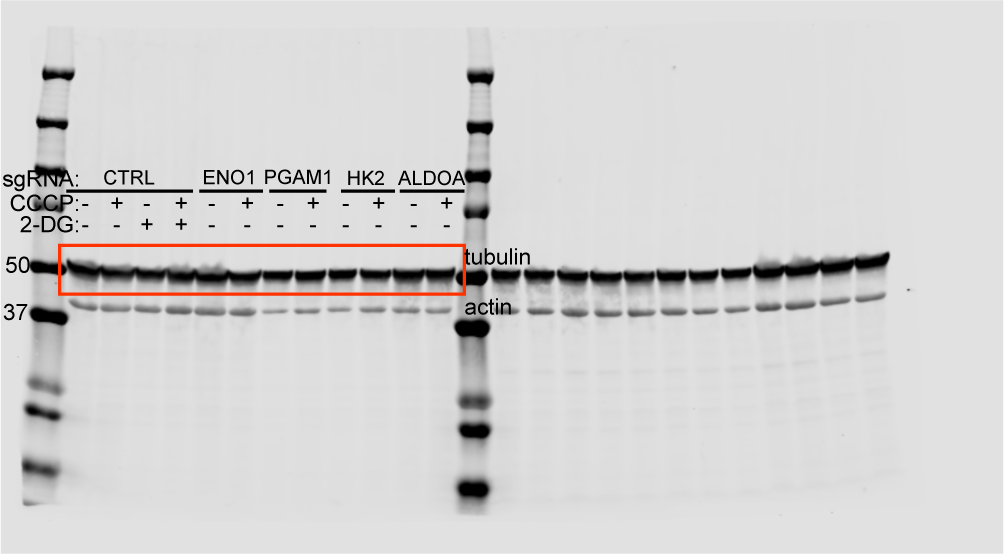

Supplement: Supplementary file 14 — Source data Fig. 2 [file 44318_2025_604_MOESM14_ESM.zip › Figure 2/2C/2C_western tubulin and actin for pS65-Ub.tif]

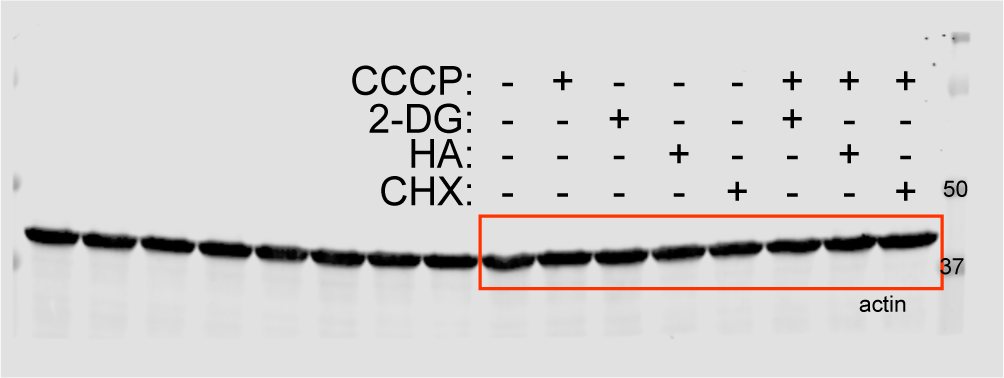

Supplement: Supplementary file 14 — Source data Fig. 2 [file 44318_2025_604_MOESM14_ESM.zip › Figure 2/2D/2D_western actin for pink1.tif]

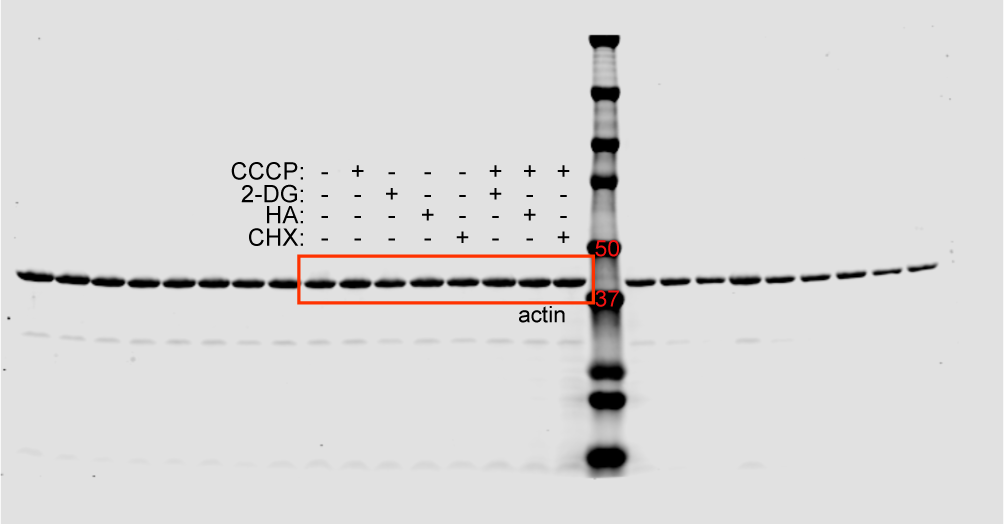

Supplement: Supplementary file 14 — Source data Fig. 2 [file 44318_2025_604_MOESM14_ESM.zip › Figure 2/2D/2D_western actin for pS65 Ub.tif]

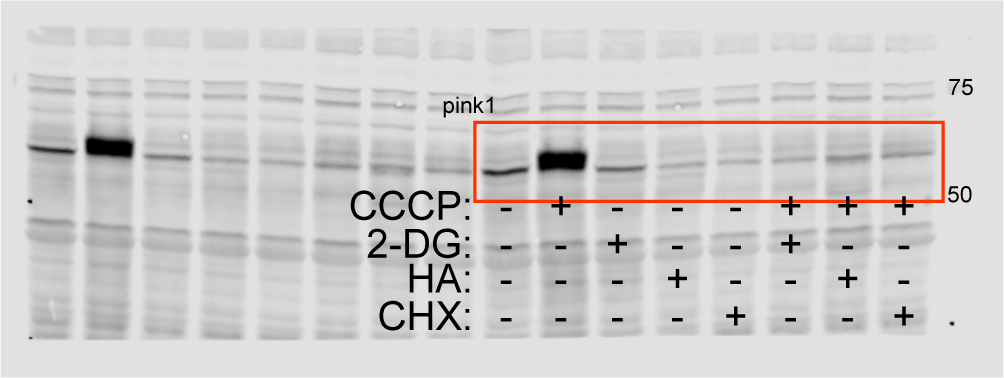

Supplement: Supplementary file 14 — Source data Fig. 2 [file 44318_2025_604_MOESM14_ESM.zip › Figure 2/2D/2D_western pink1.tif]

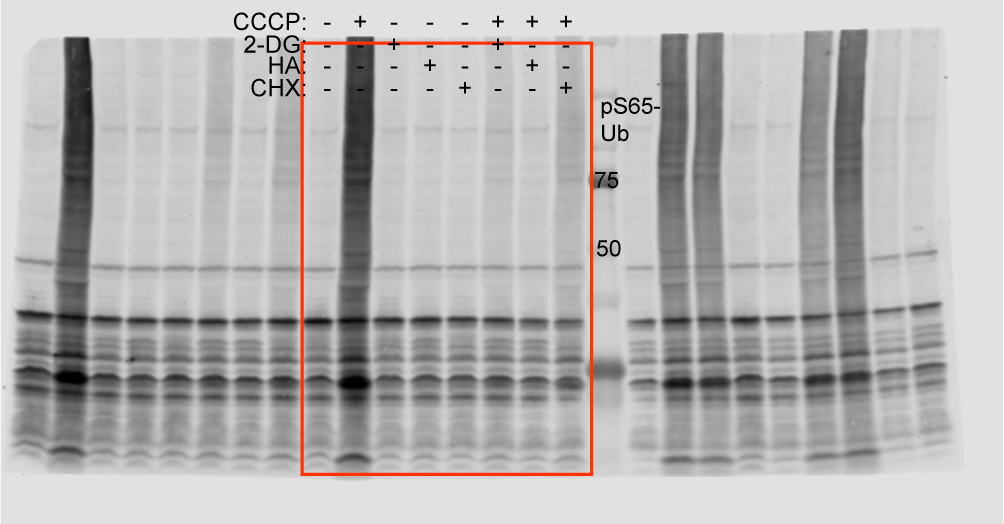

Supplement: Supplementary file 14 — Source data Fig. 2 [file 44318_2025_604_MOESM14_ESM.zip › Figure 2/2D/2D_western pS65 Ub.tif]

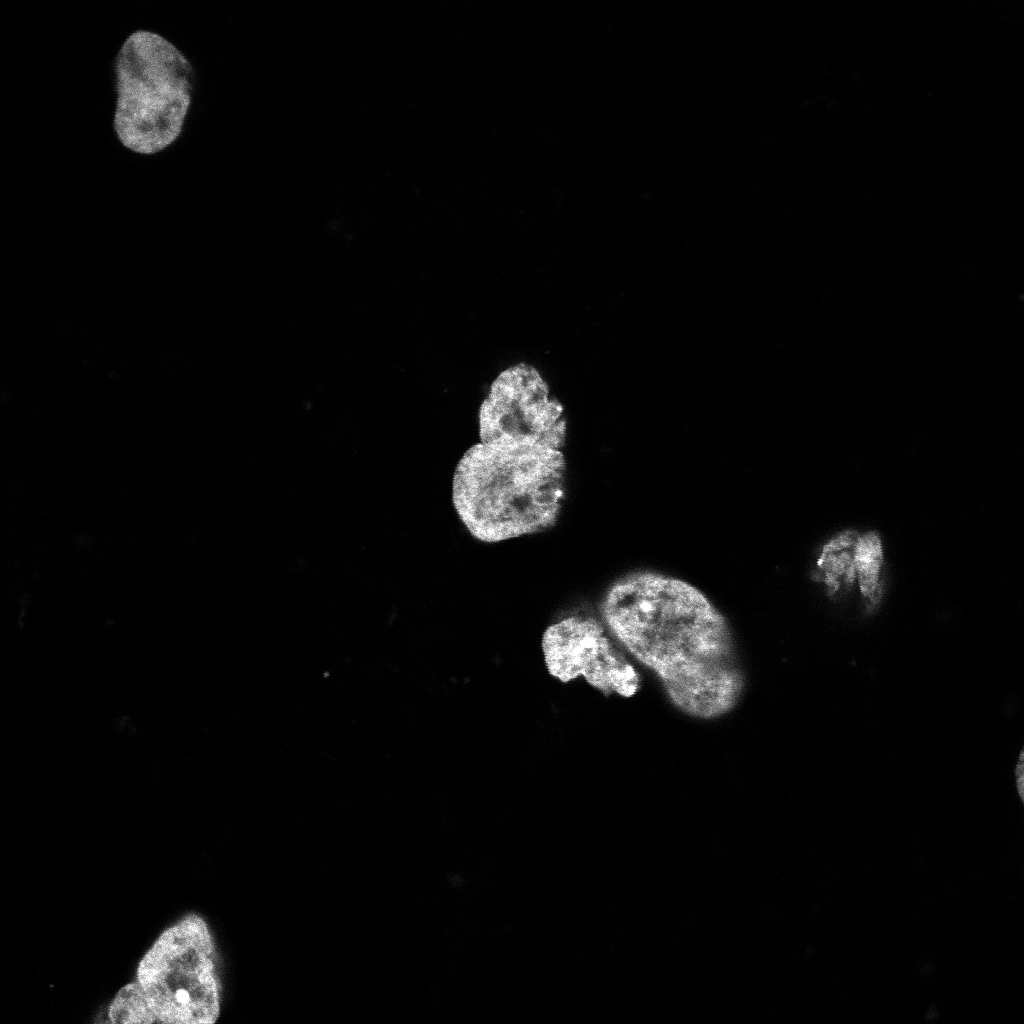

Supplement: Supplementary file 14 — Source data Fig. 2 [file 44318_2025_604_MOESM14_ESM.zip › Figure 2/2E/2E_image_ C=0_DAPI.tif]

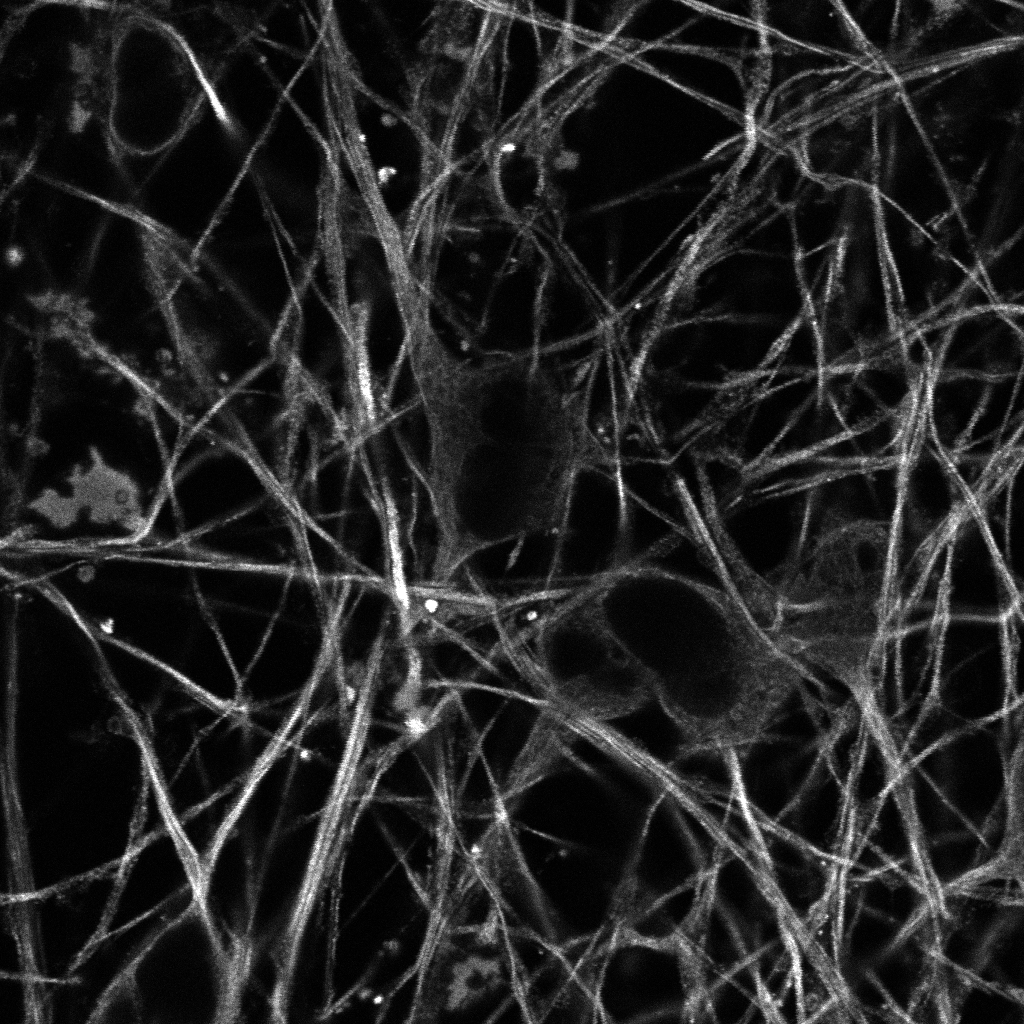

Supplement: Supplementary file 14 — Source data Fig. 2 [file 44318_2025_604_MOESM14_ESM.zip › Figure 2/2E/2E_image_C=1_TUBB3.tif]

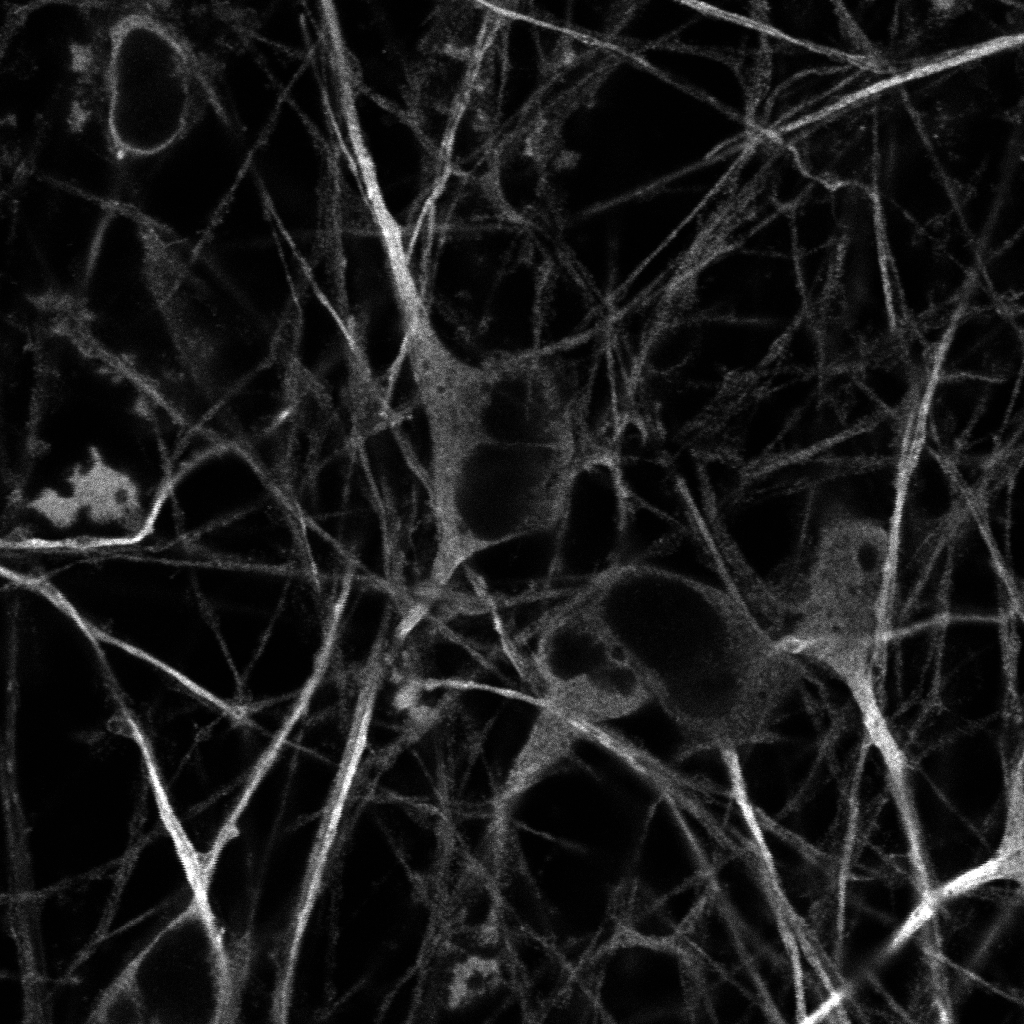

Supplement: Supplementary file 14 — Source data Fig. 2 [file 44318_2025_604_MOESM14_ESM.zip › Figure 2/2E/2E_image_C=2_MAP2.tif]

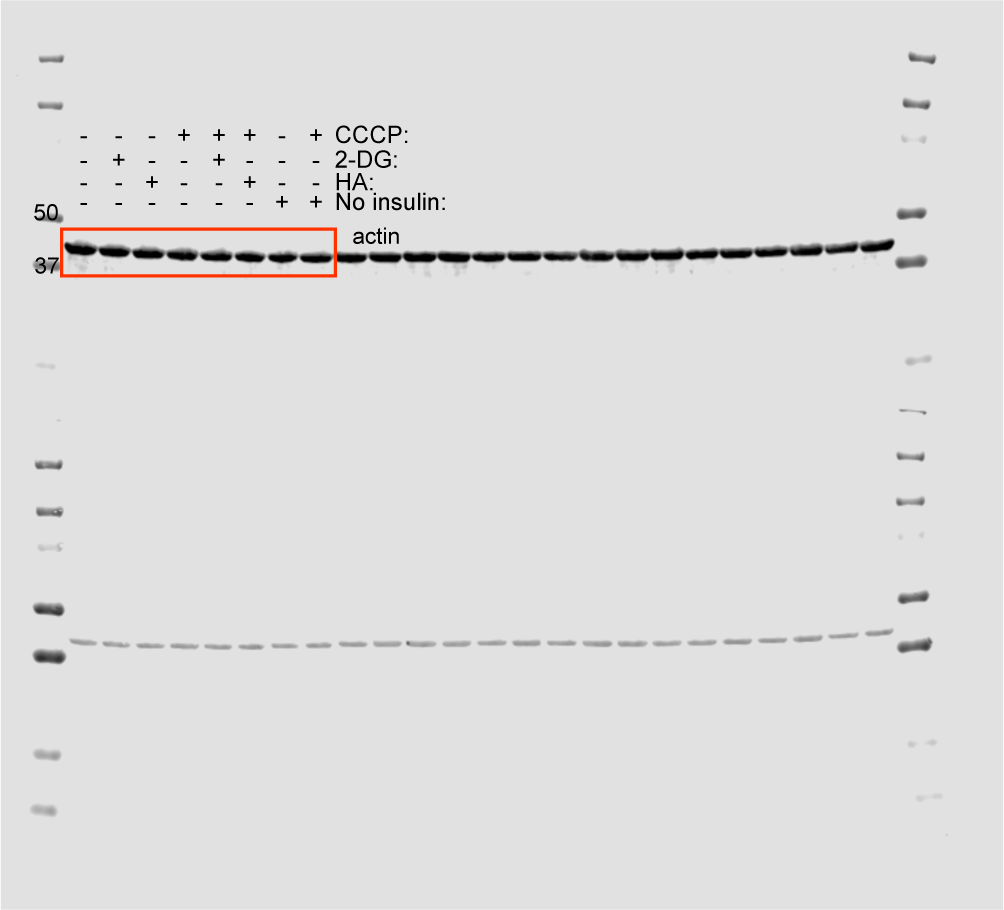

Supplement: Supplementary file 14 — Source data Fig. 2 [file 44318_2025_604_MOESM14_ESM.zip › Figure 2/2F/2F_western actin for mfn2.tif]

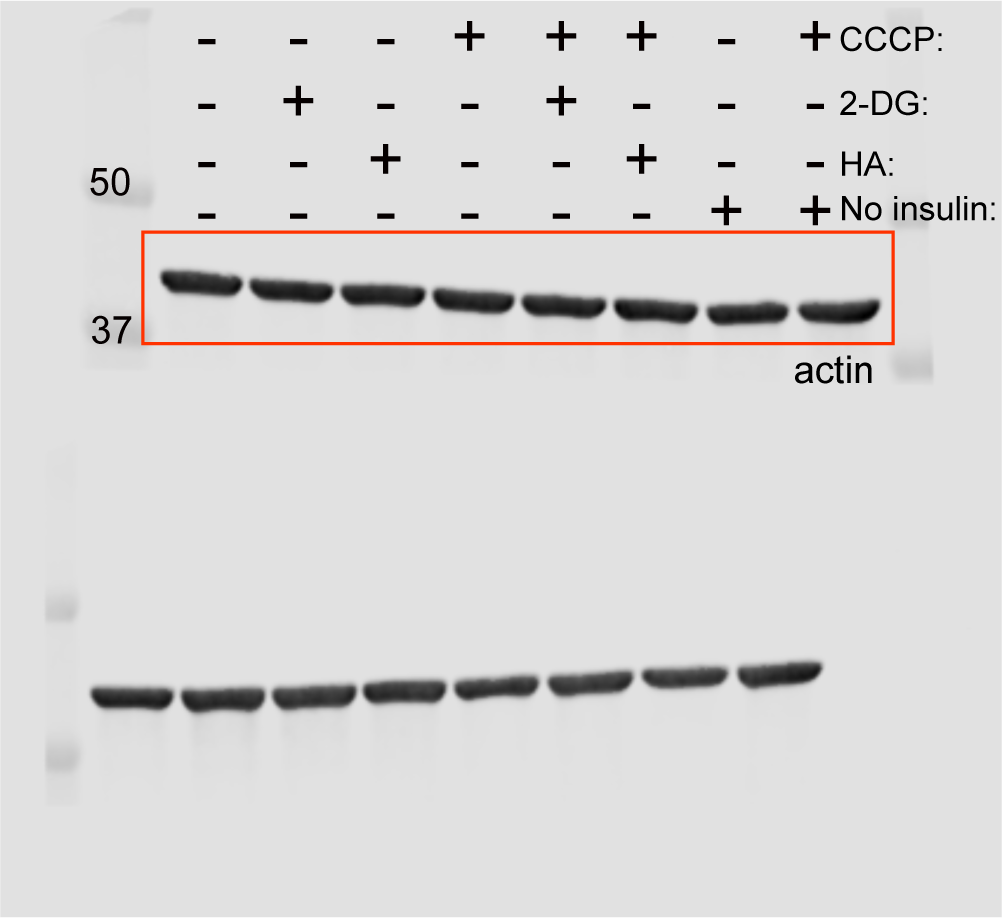

Supplement: Supplementary file 14 — Source data Fig. 2 [file 44318_2025_604_MOESM14_ESM.zip › Figure 2/2F/2F_western actin for pink1.tif]

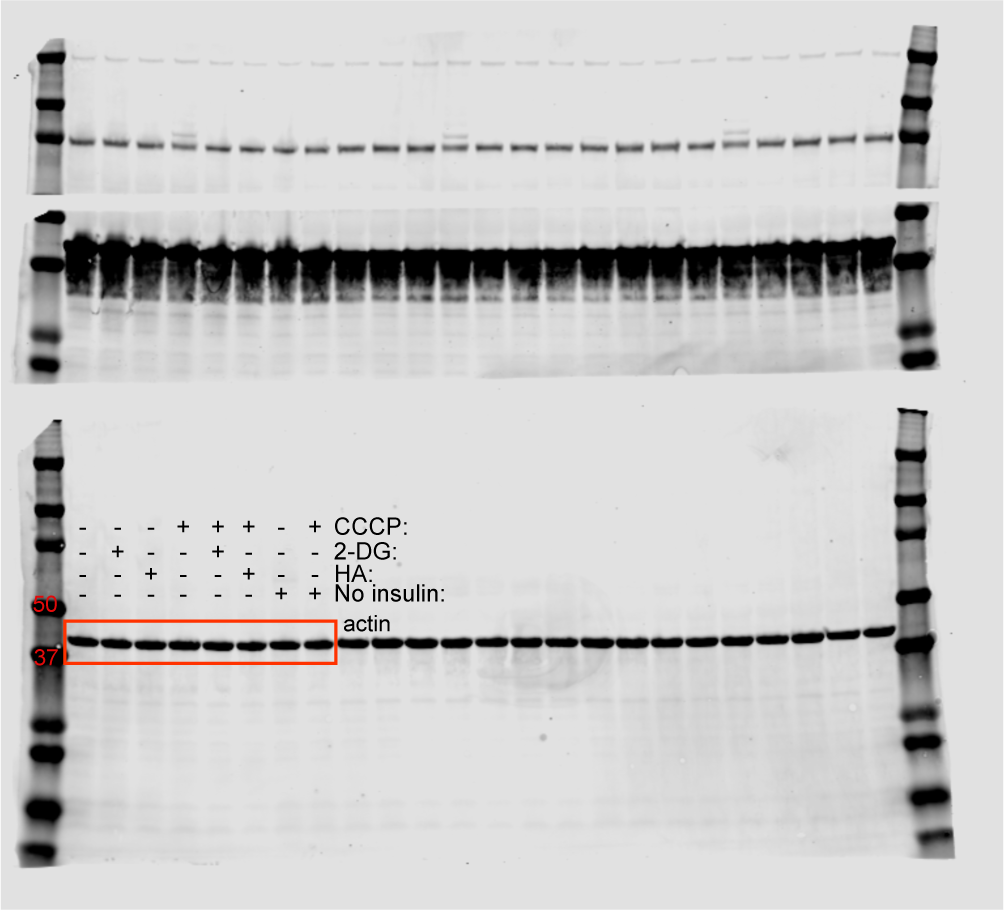

Supplement: Supplementary file 14 — Source data Fig. 2 [file 44318_2025_604_MOESM14_ESM.zip › Figure 2/2F/2F_western actin for pS65 Ub.tif]

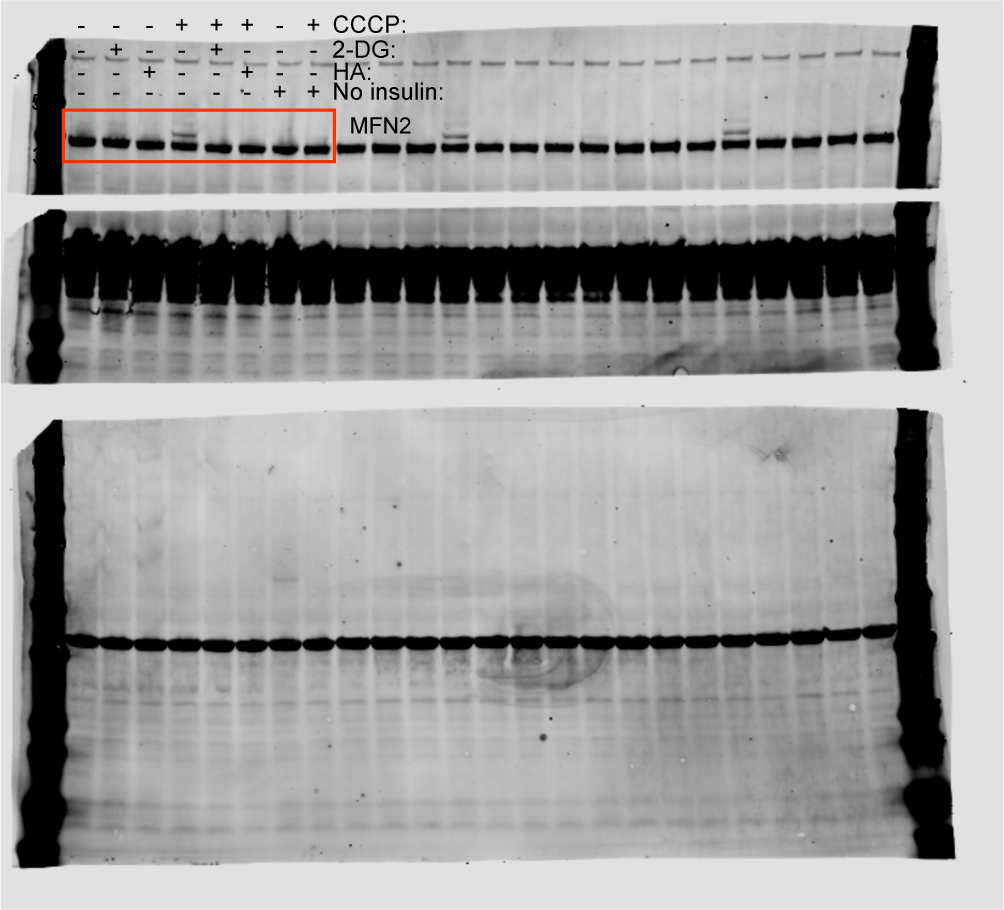

Supplement: Supplementary file 14 — Source data Fig. 2 [file 44318_2025_604_MOESM14_ESM.zip › Figure 2/2F/2F_western mfn2.tif]

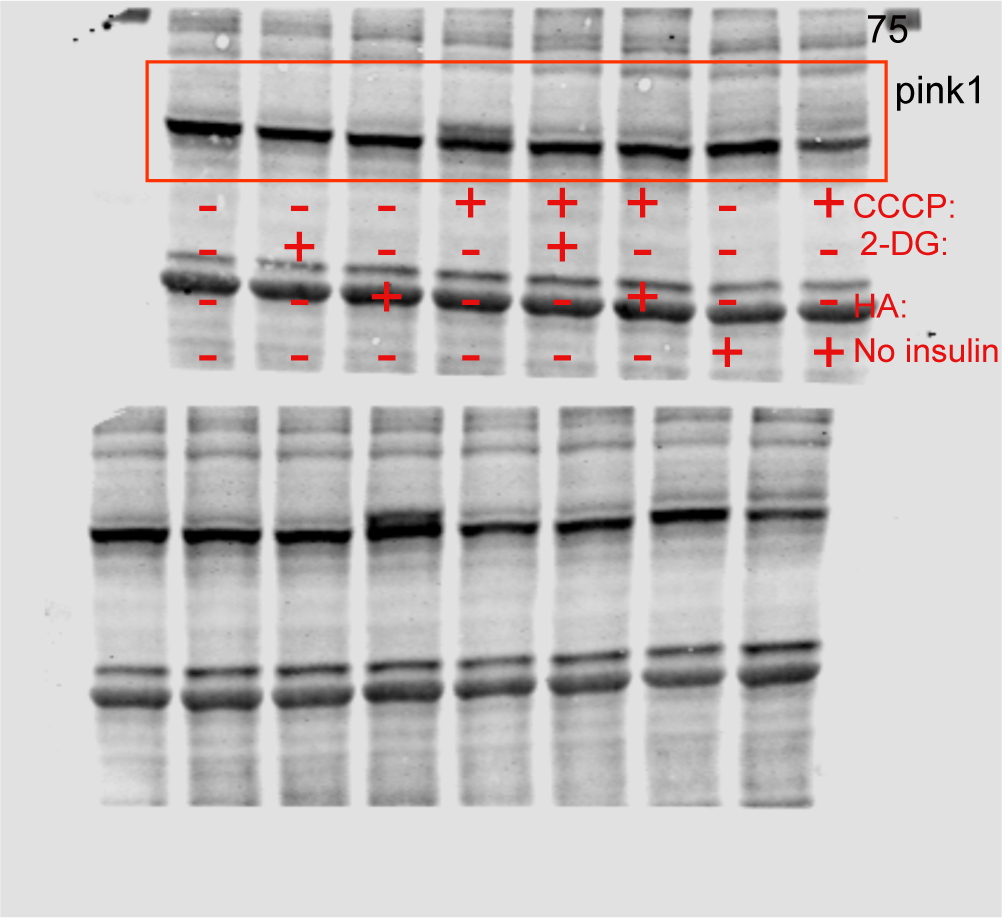

Supplement: Supplementary file 14 — Source data Fig. 2 [file 44318_2025_604_MOESM14_ESM.zip › Figure 2/2F/2F_western pink1.tif]

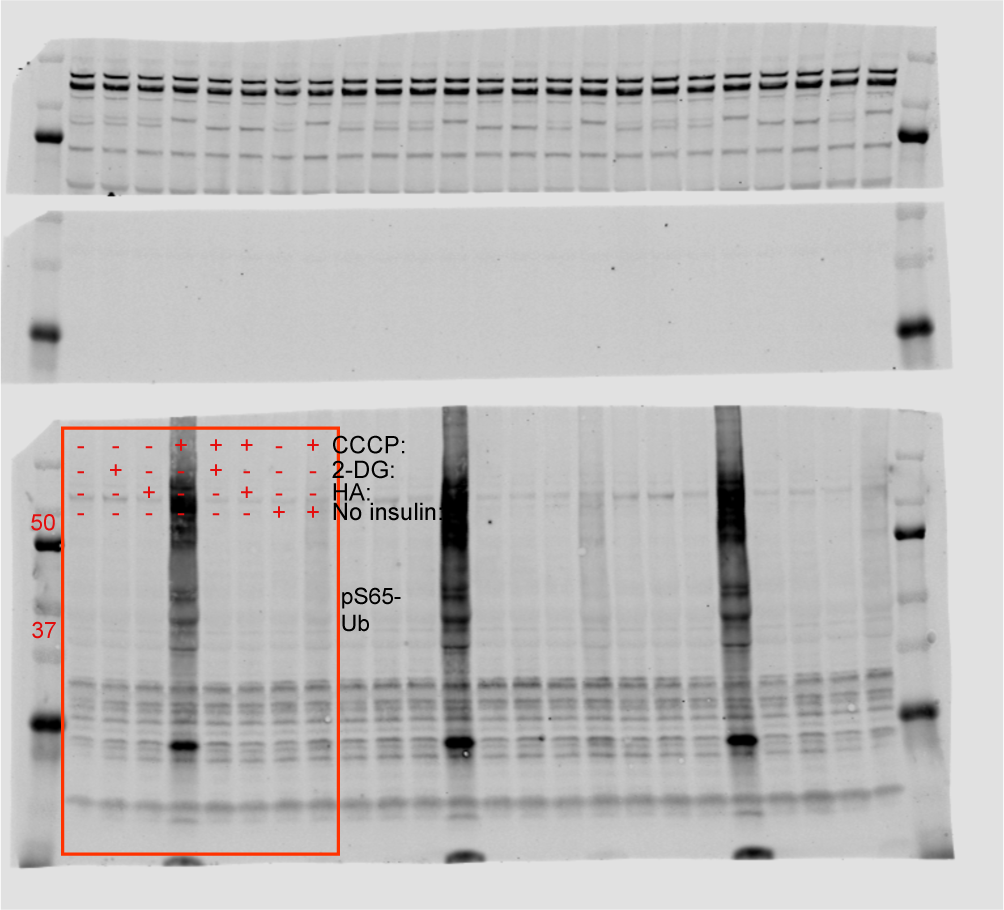

Supplement: Supplementary file 14 — Source data Fig. 2 [file 44318_2025_604_MOESM14_ESM.zip › Figure 2/2F/2F_western pS65 Ub.tif]

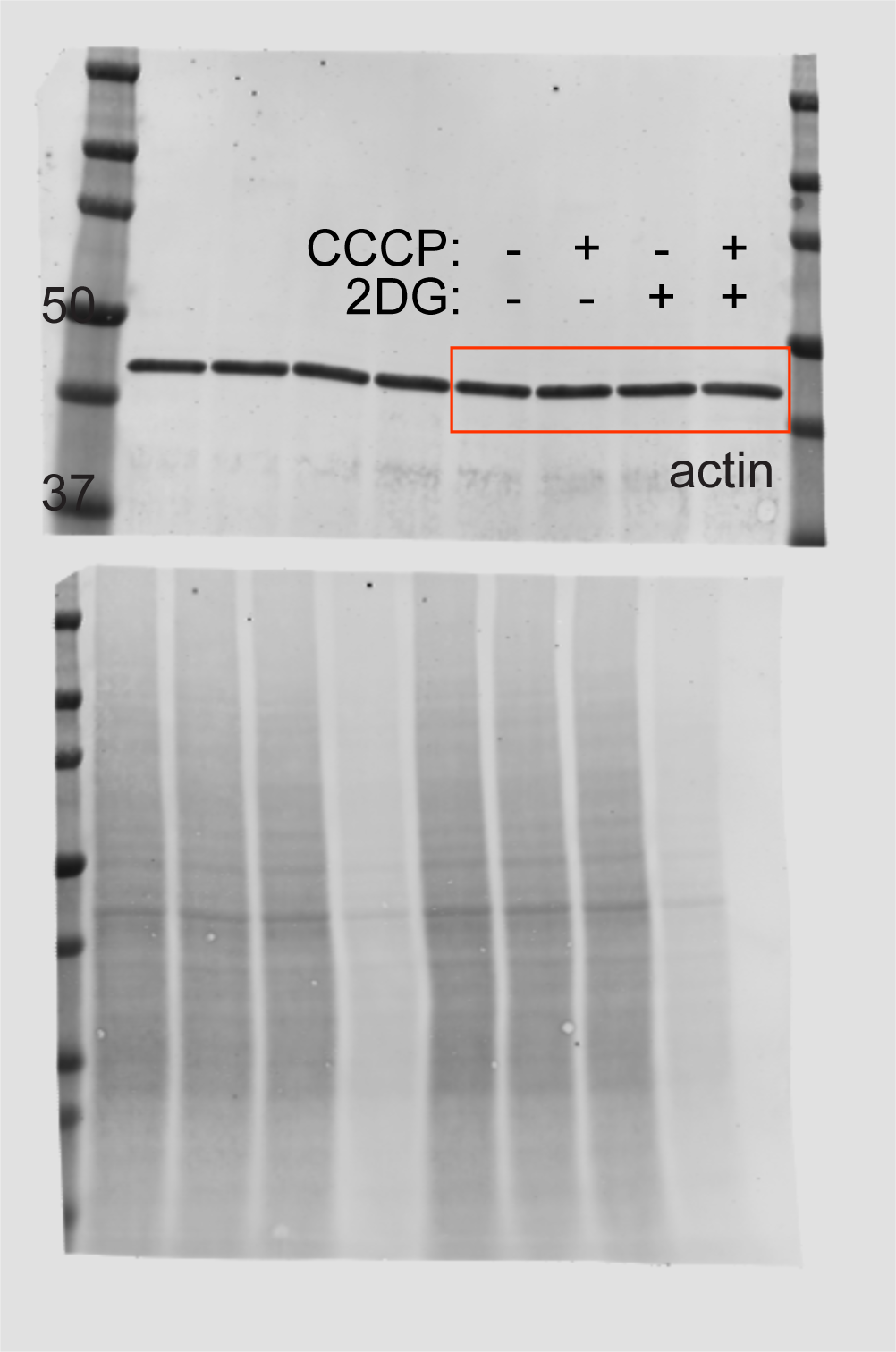

Supplement: Supplementary file 14 — Source data Fig. 2 [file 44318_2025_604_MOESM14_ESM.zip › Figure 2/2G/2G_western actin for pink1.tif]

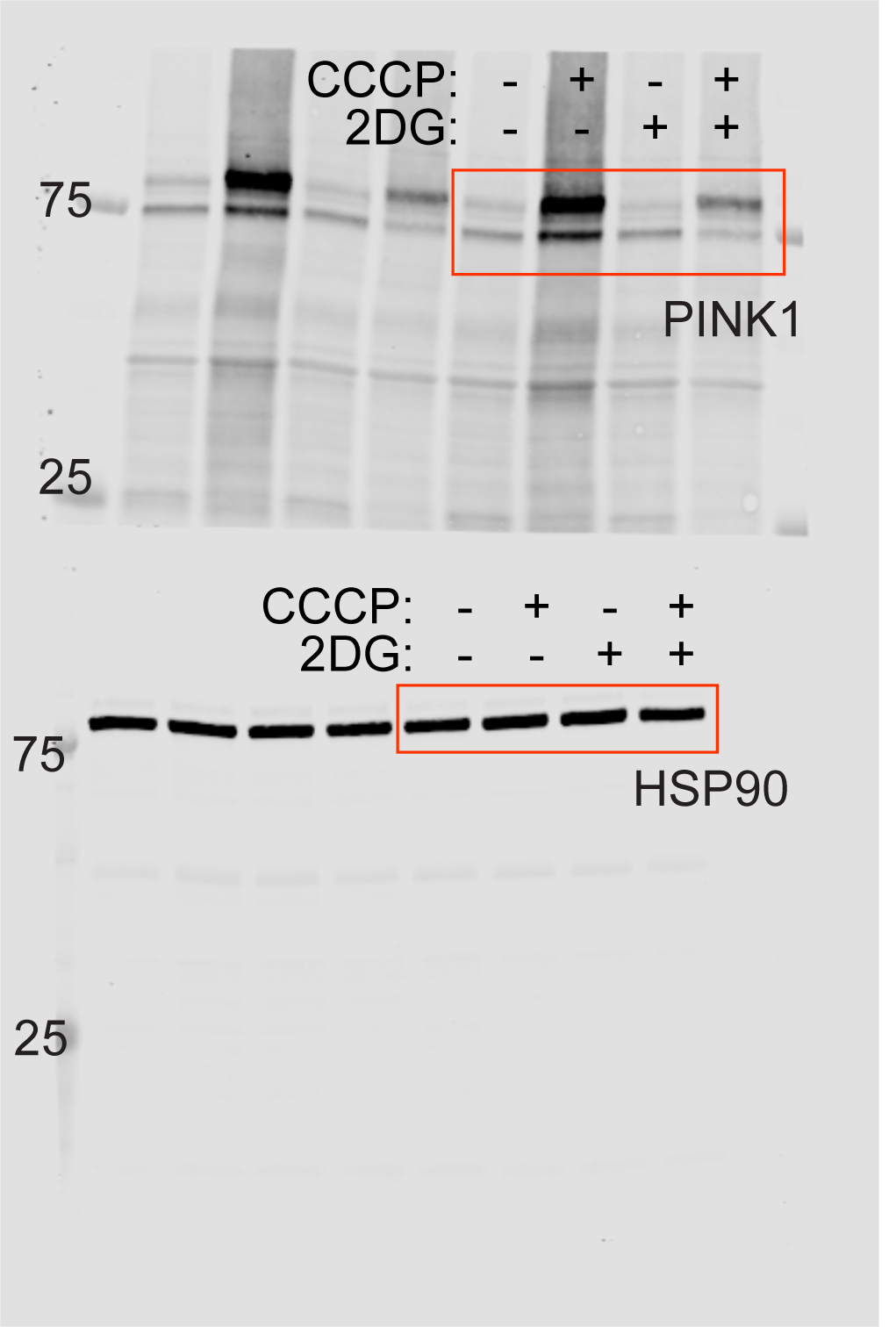

Supplement: Supplementary file 14 — Source data Fig. 2 [file 44318_2025_604_MOESM14_ESM.zip › Figure 2/2G/2G_western pink1 and hsp90 for puro.tif]

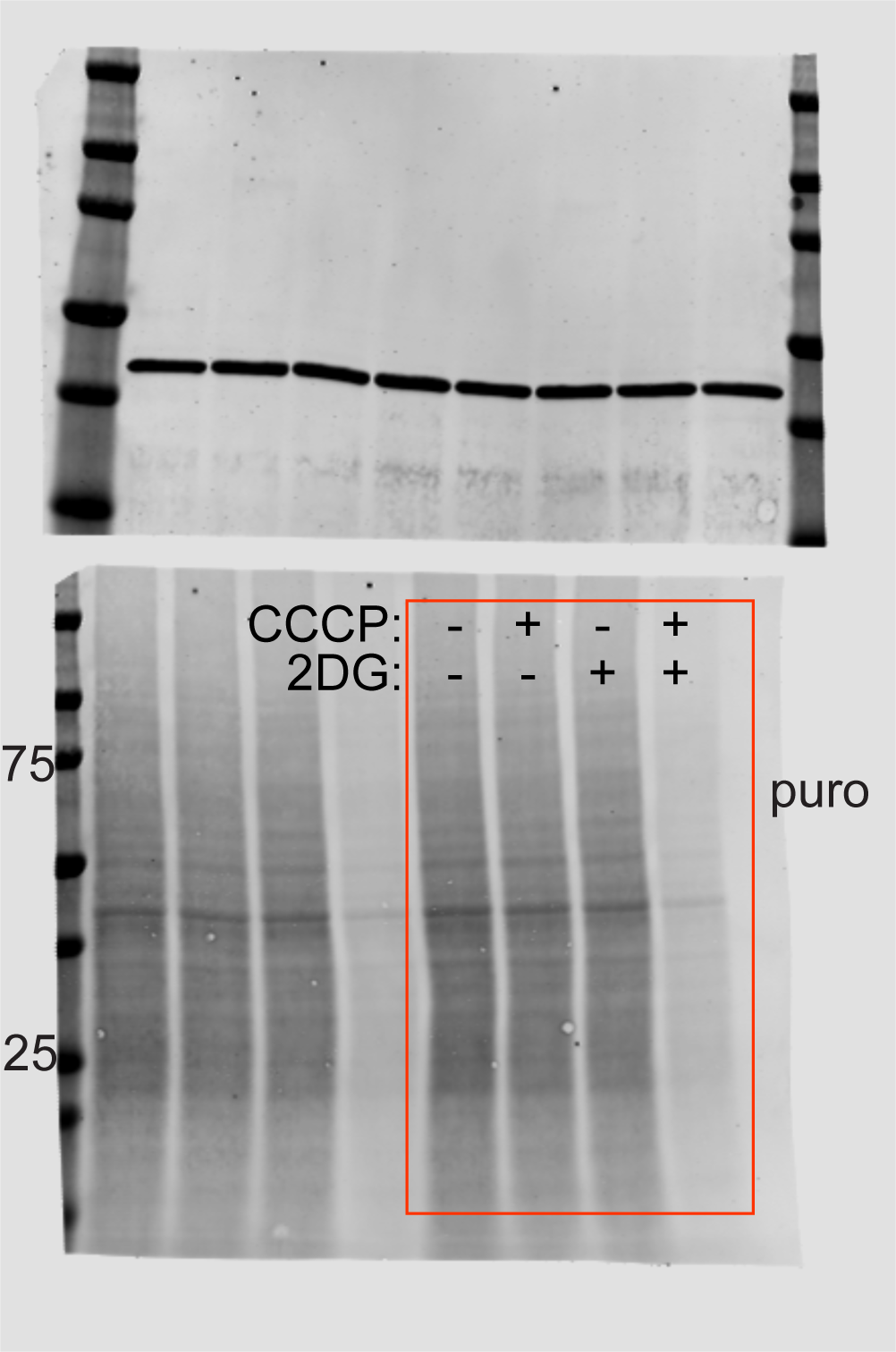

Supplement: Supplementary file 14 — Source data Fig. 2 [file 44318_2025_604_MOESM14_ESM.zip › Figure 2/2G/2G_western puro.tif]

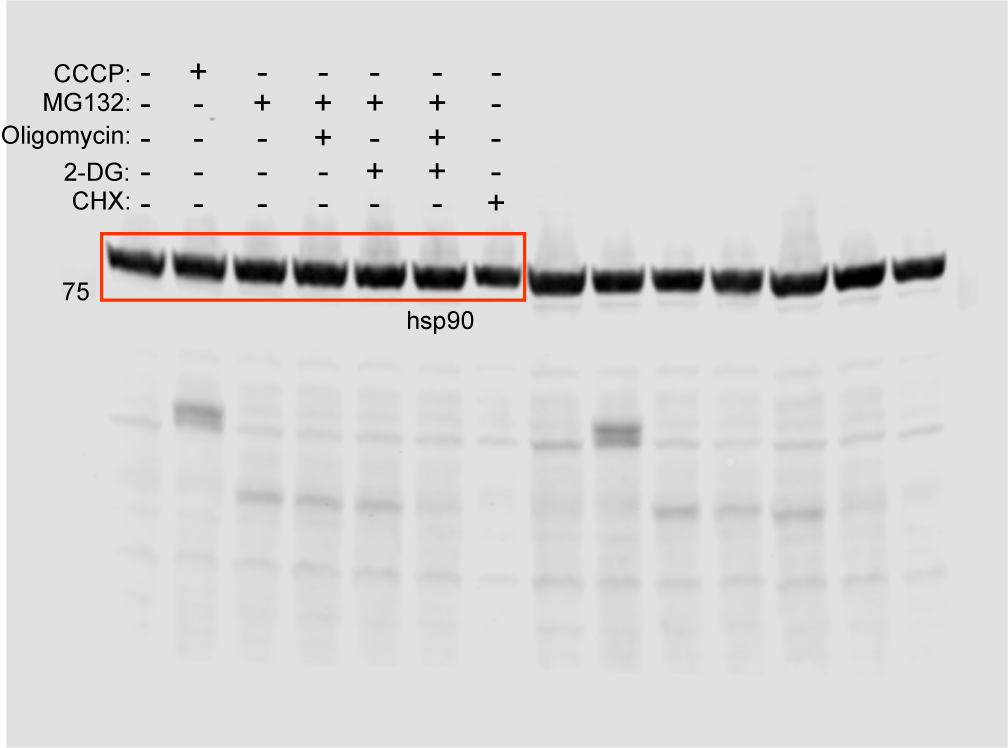

Supplement: Supplementary file 14 — Source data Fig. 2 [file 44318_2025_604_MOESM14_ESM.zip › Figure 2/2H/2H_western hsp90.tif]

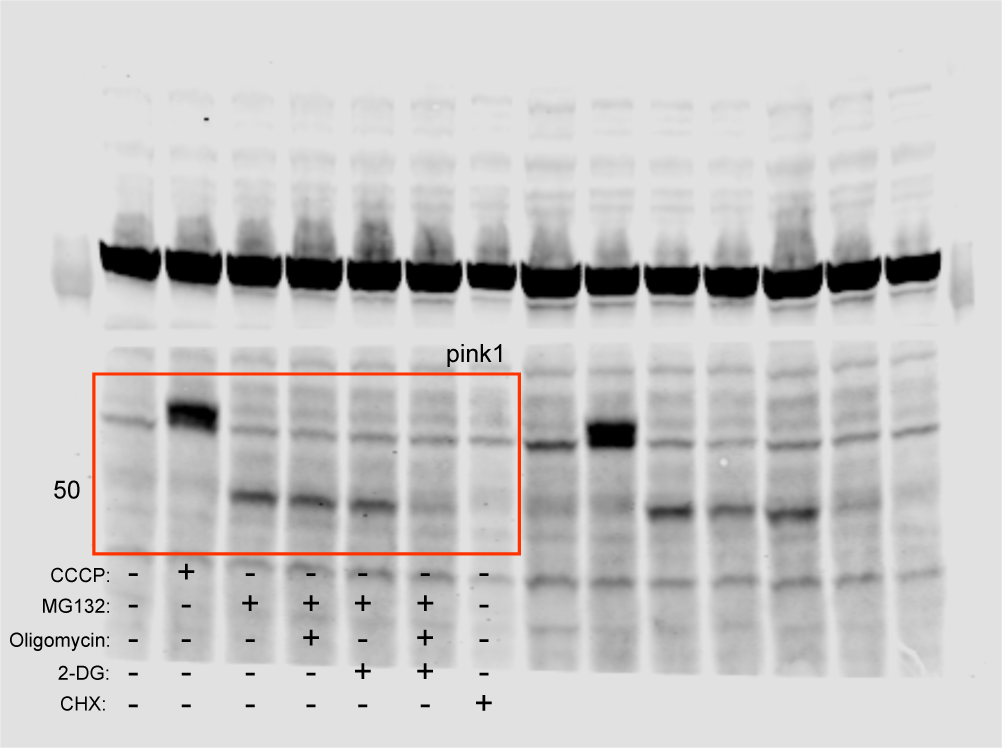

Supplement: Supplementary file 14 — Source data Fig. 2 [file 44318_2025_604_MOESM14_ESM.zip › Figure 2/2H/2H_western pink1.tif]

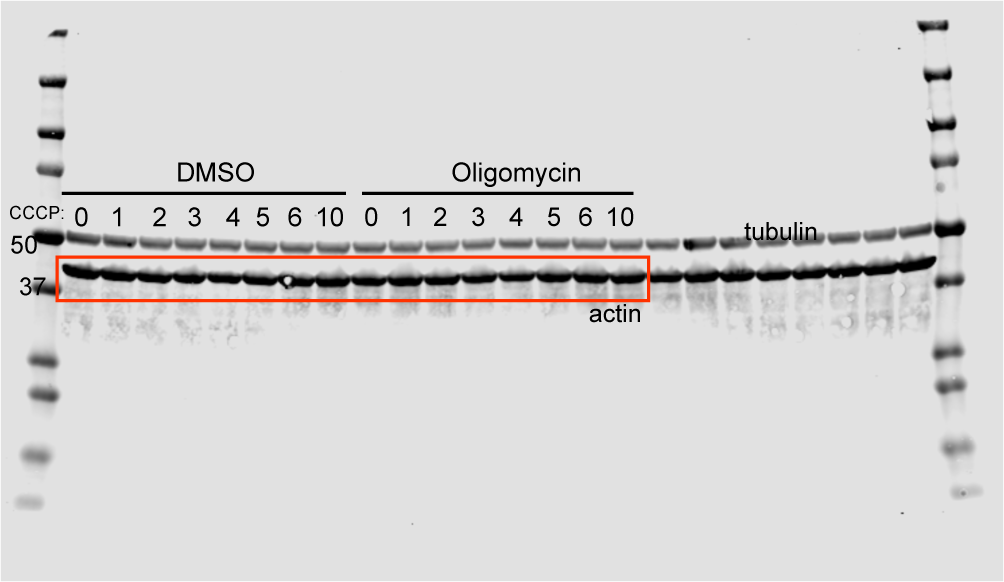

Supplement: Supplementary file 14 — Source data Fig. 2 [file 44318_2025_604_MOESM14_ESM.zip › Figure 2/2J/2J_western actin for pS65 Ub.tif]

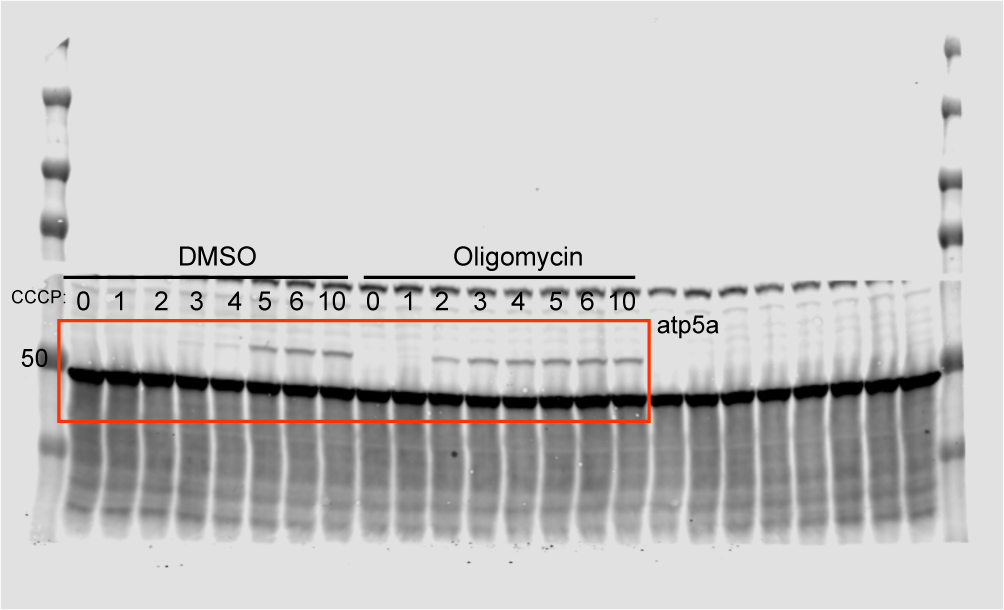

Supplement: Supplementary file 14 — Source data Fig. 2 [file 44318_2025_604_MOESM14_ESM.zip › Figure 2/2J/2J_western atp5a.tif]

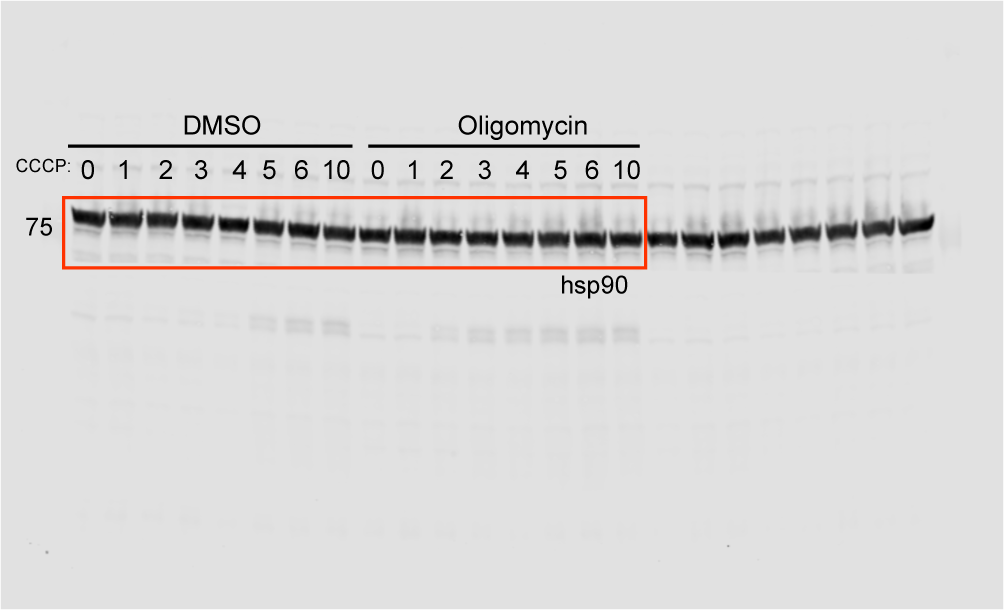

Supplement: Supplementary file 14 — Source data Fig. 2 [file 44318_2025_604_MOESM14_ESM.zip › Figure 2/2J/2J_western hsp90 for atp5a and pink1.tif]

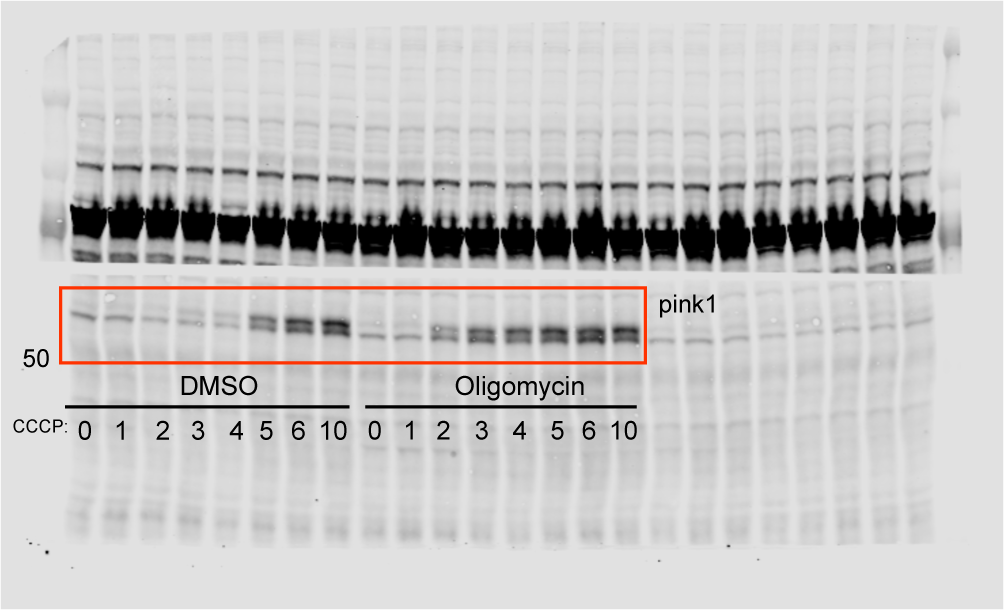

Supplement: Supplementary file 14 — Source data Fig. 2 [file 44318_2025_604_MOESM14_ESM.zip › Figure 2/2J/2J_western PINK1.tif]

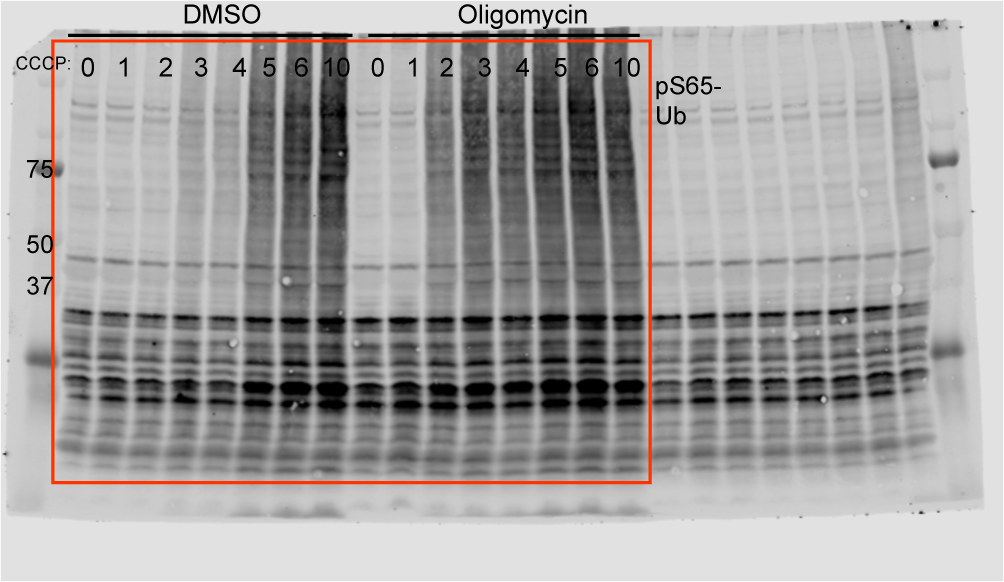

Supplement: Supplementary file 14 — Source data Fig. 2 [file 44318_2025_604_MOESM14_ESM.zip › Figure 2/2J/2J_western pS65 Ub.tif]

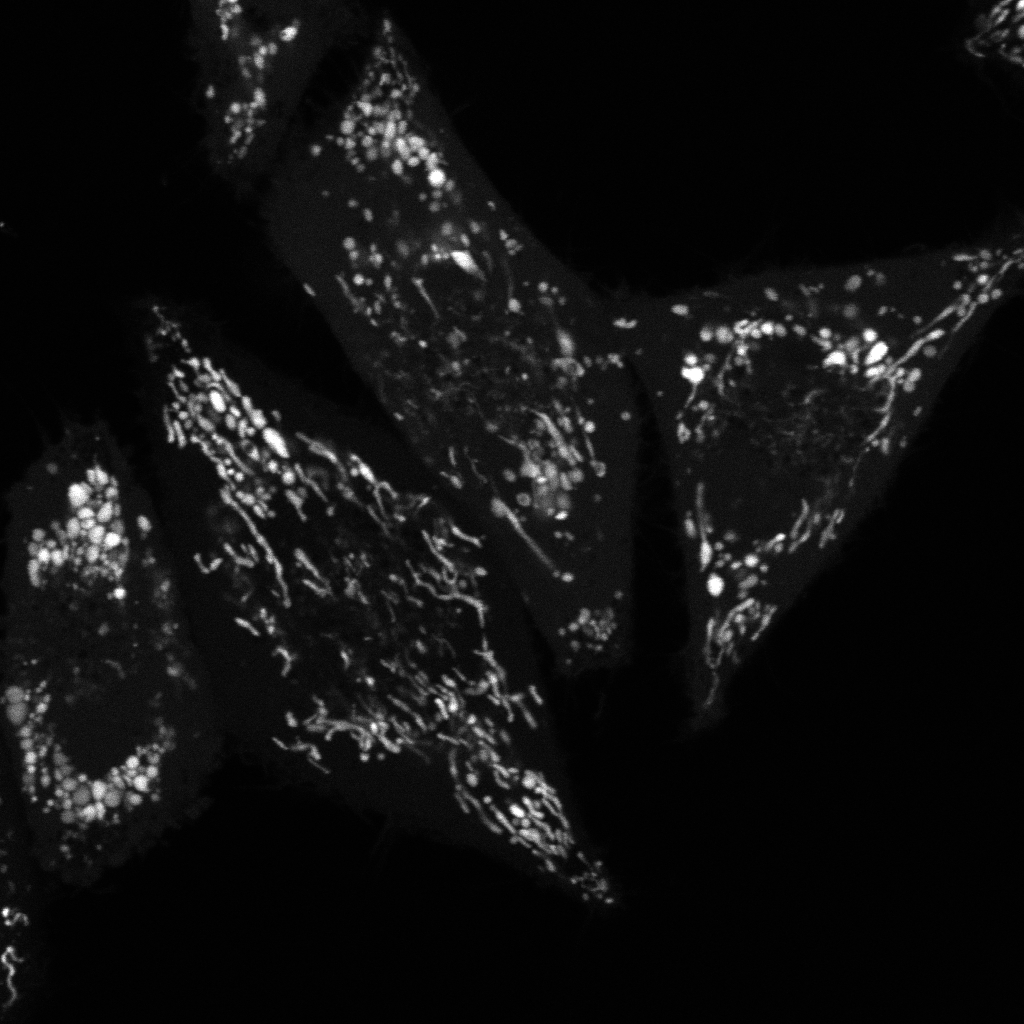

Supplement: Supplementary file 15 — Source data Fig. 3 - A and E-H [file 44318_2025_604_MOESM15_ESM.zip › Figure 3 - A and E-H/3E/3E_image_NDUFAB1sgRNA_7days_live_channel3_MTSCherry.tif]

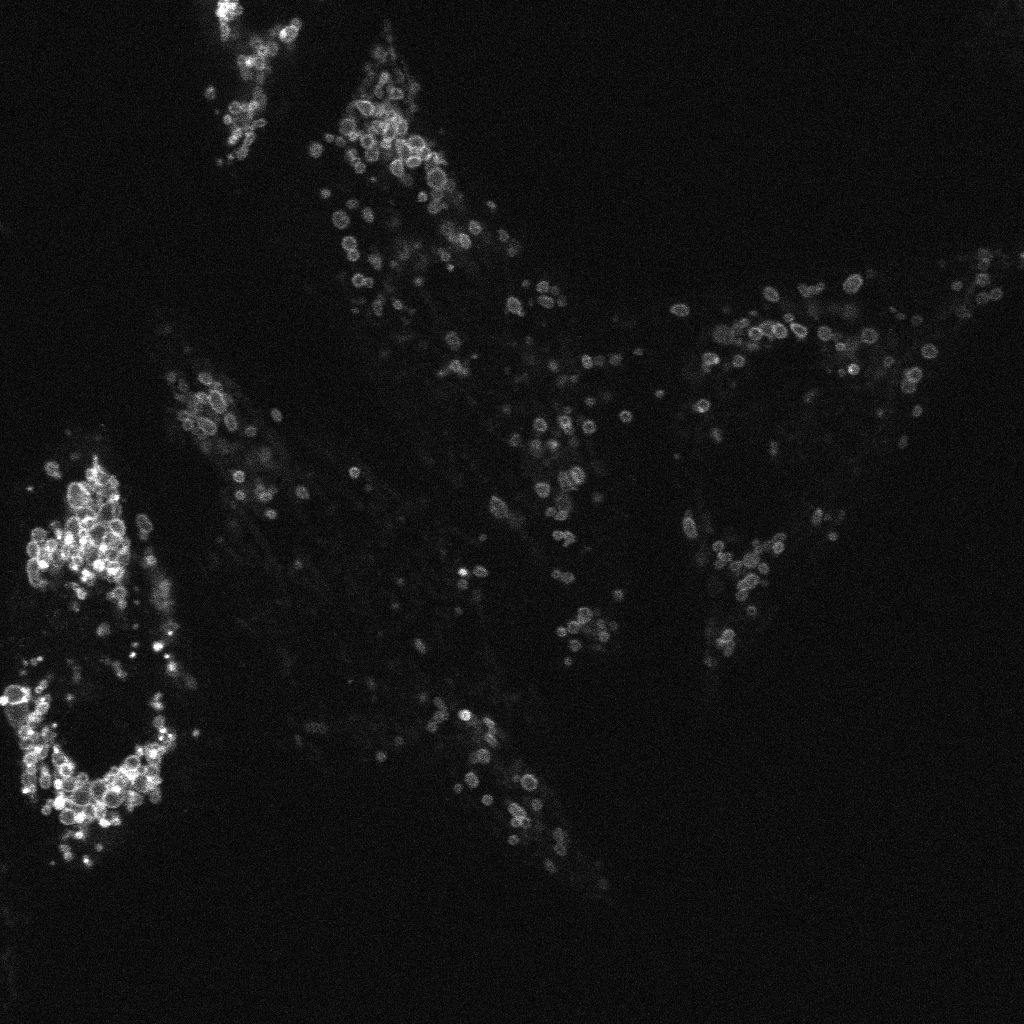

Supplement: Supplementary file 15 — Source data Fig. 3 - A and E-H [file 44318_2025_604_MOESM15_ESM.zip › Figure 3 - A and E-H/3E/3E_image_NDUFAB1sgRNA_7days_live_channel2_PINK1YFP.tif]

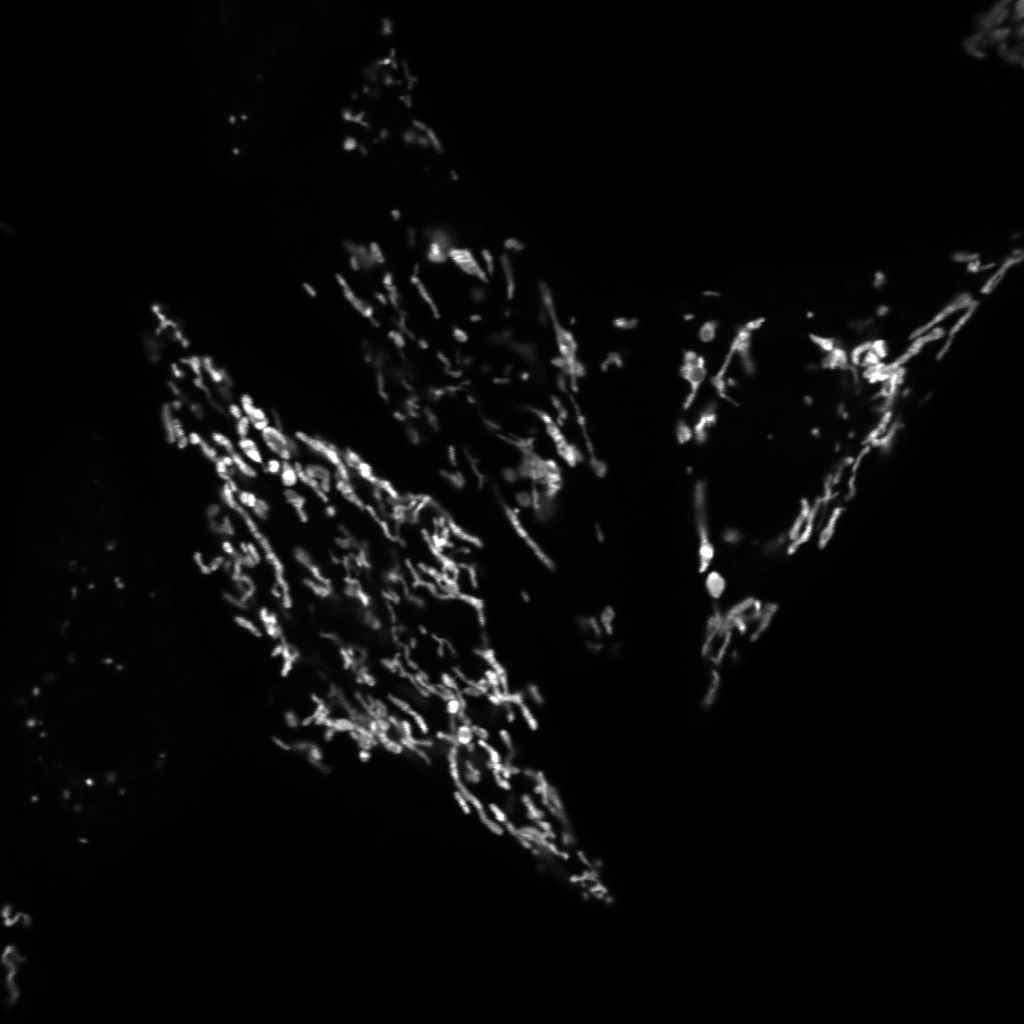

Supplement: Supplementary file 15 — Source data Fig. 3 - A and E-H [file 44318_2025_604_MOESM15_ESM.zip › Figure 3 - A and E-H/3E/3E_image_NDUFAB1sgRNA_7days_live_channel4_MitoLiteNIR.tif]

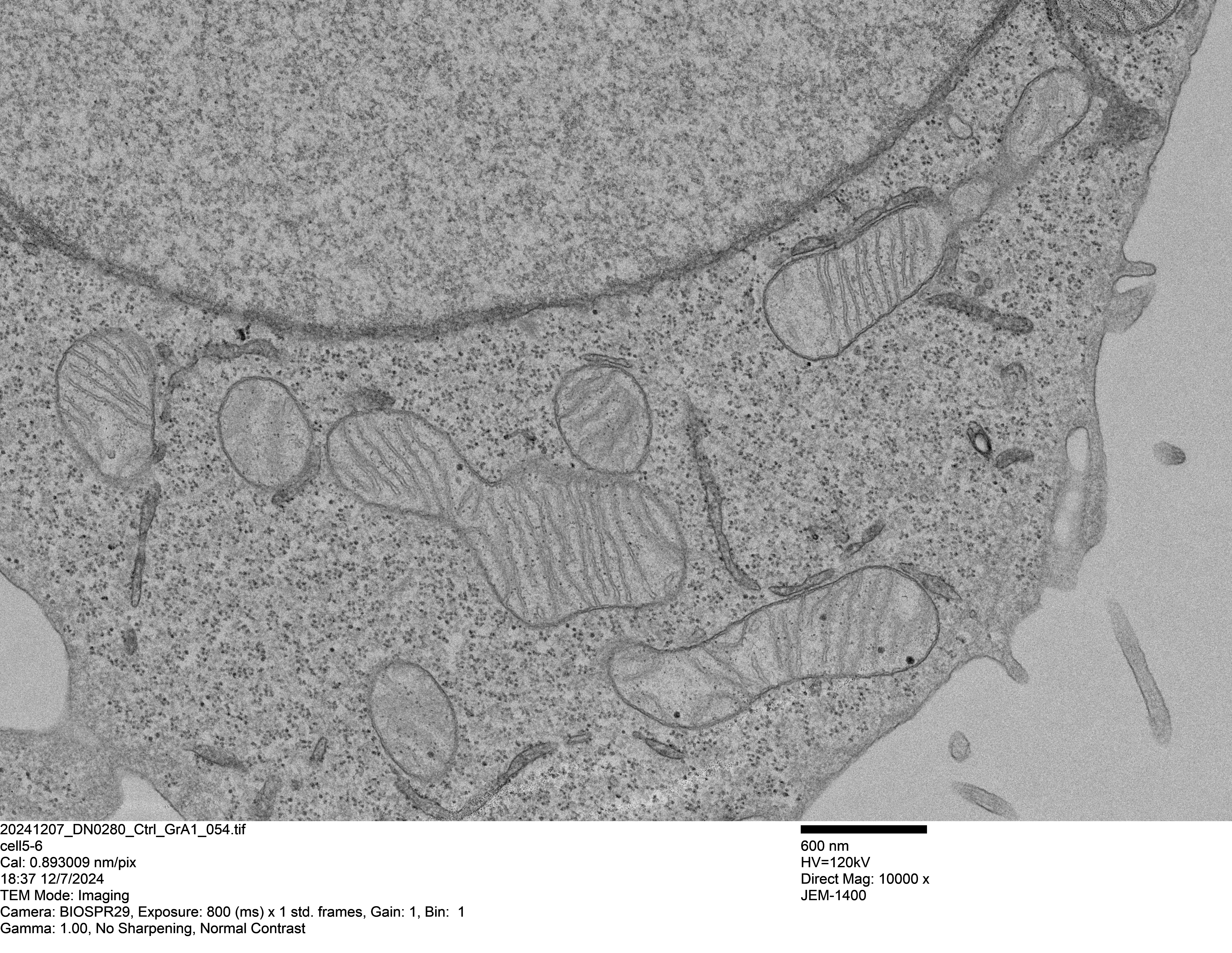

Supplement: Supplementary file 16 — Source data Fig. 3C Part 1 [file 44318_2025_604_MOESM16_ESM.zip › Fig3C_part1/3C_image_Ctrl.tif]

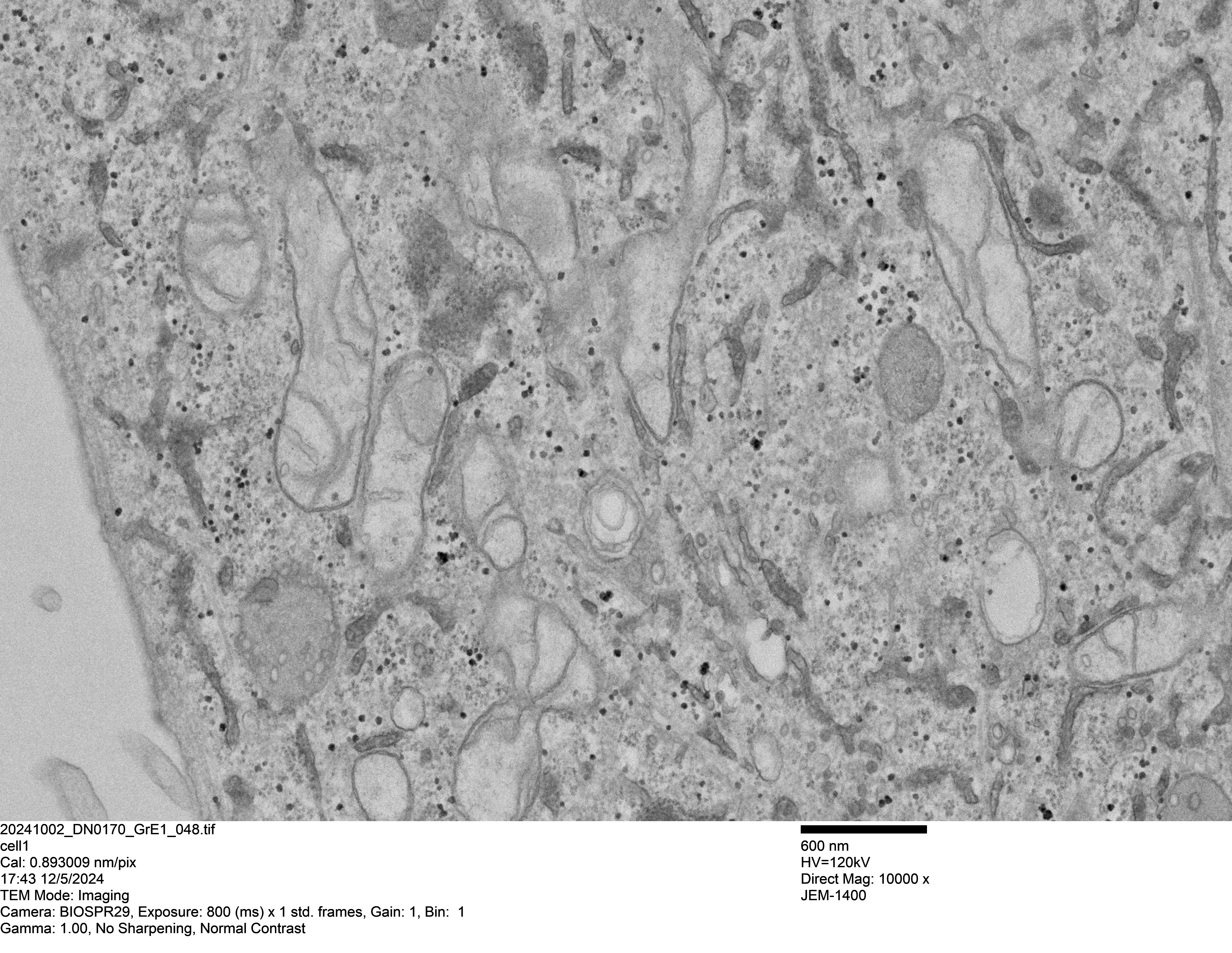

Supplement: Supplementary file 17 — Source data Fig. 3C Part 2 [file 44318_2025_604_MOESM17_ESM.zip › Fig3C_part2/3C_image_ISCA2.tif]

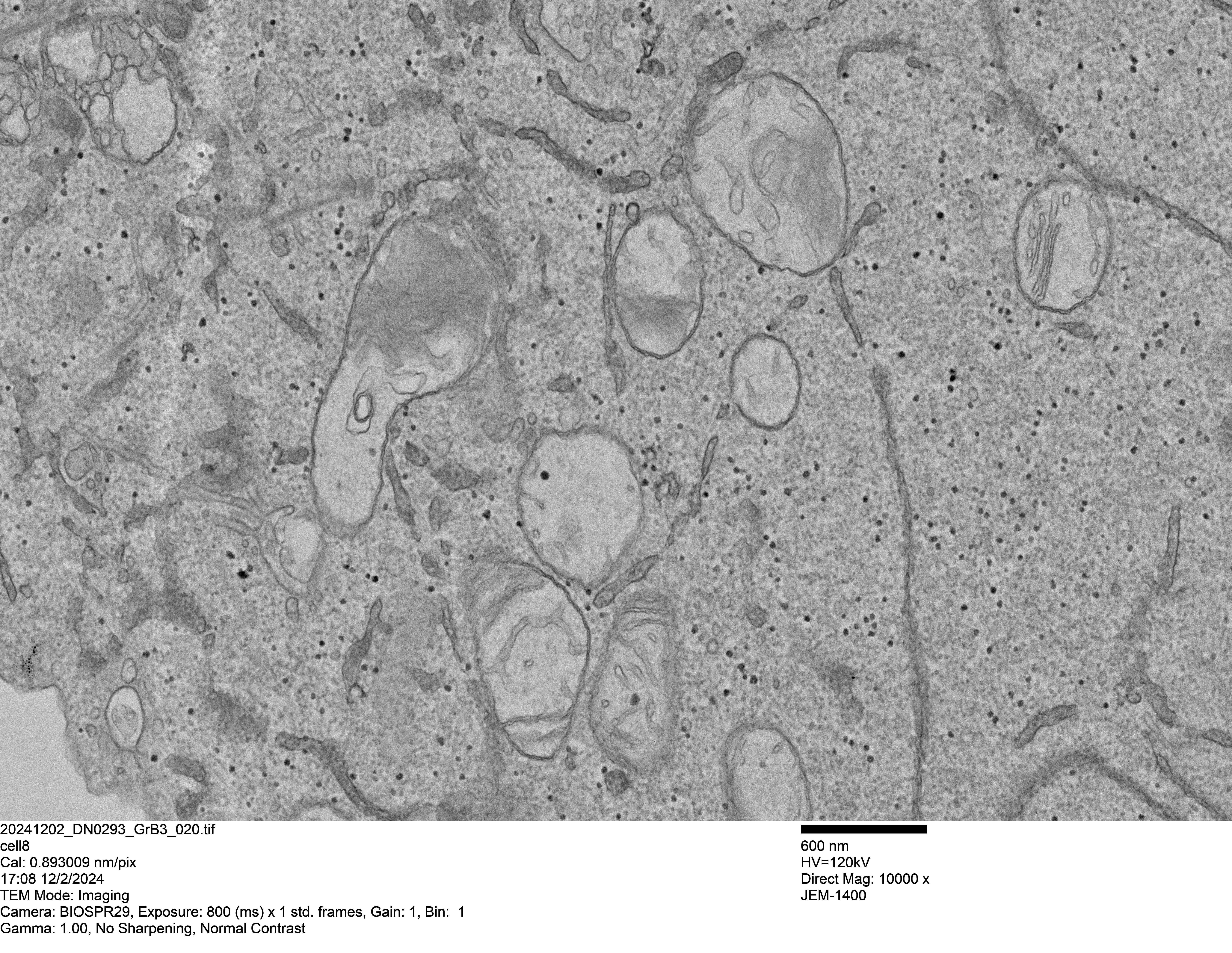

Supplement: Supplementary file 18 — Source data Fig. 3C Part 3 [file 44318_2025_604_MOESM18_ESM.zip › Fig3C_part3/3C_image_PMPCB.tif]

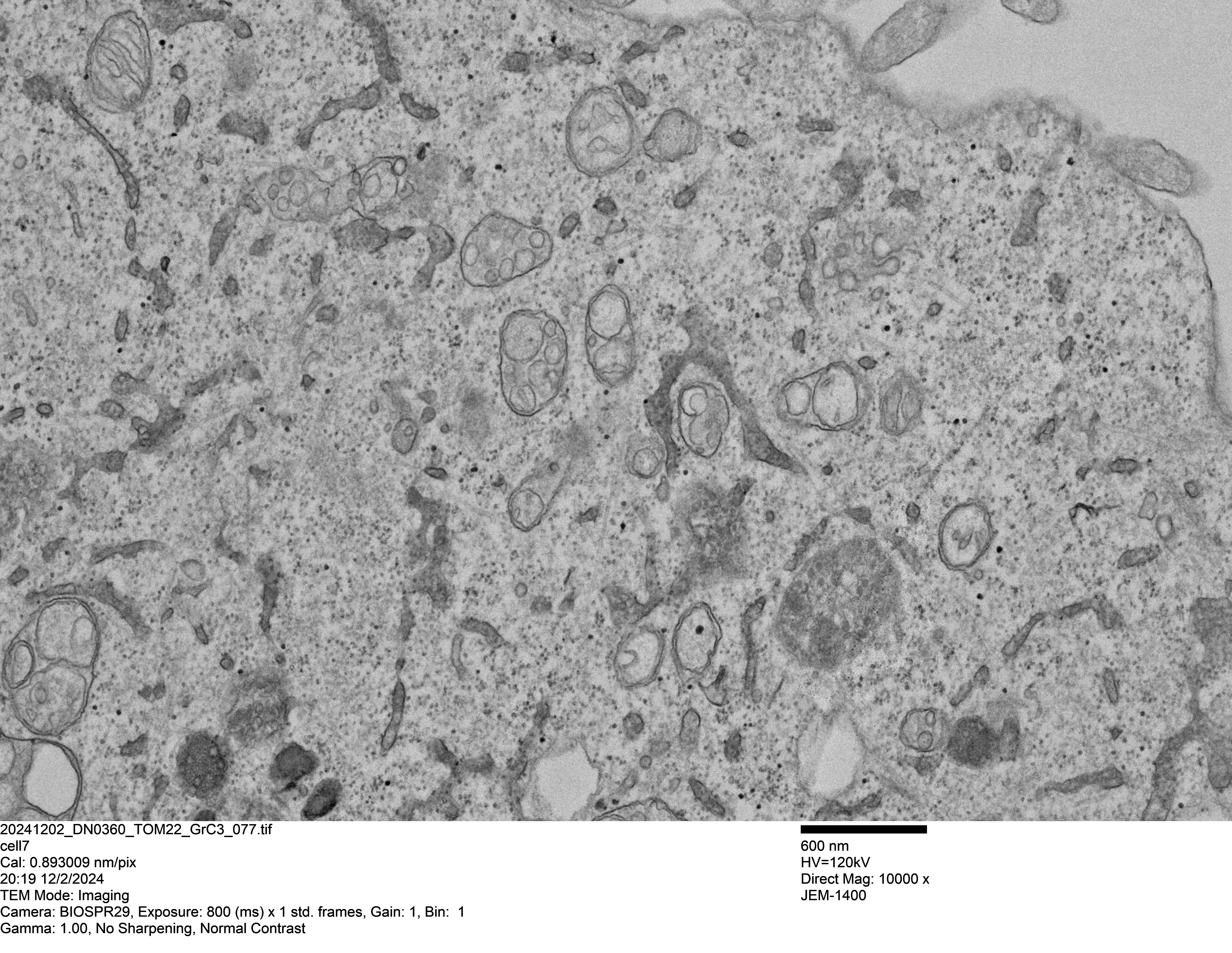

Supplement: Supplementary file 18 — Source data Fig. 3C Part 3 [file 44318_2025_604_MOESM18_ESM.zip › Fig3C_part3/3C_image_TOMM22.tif]

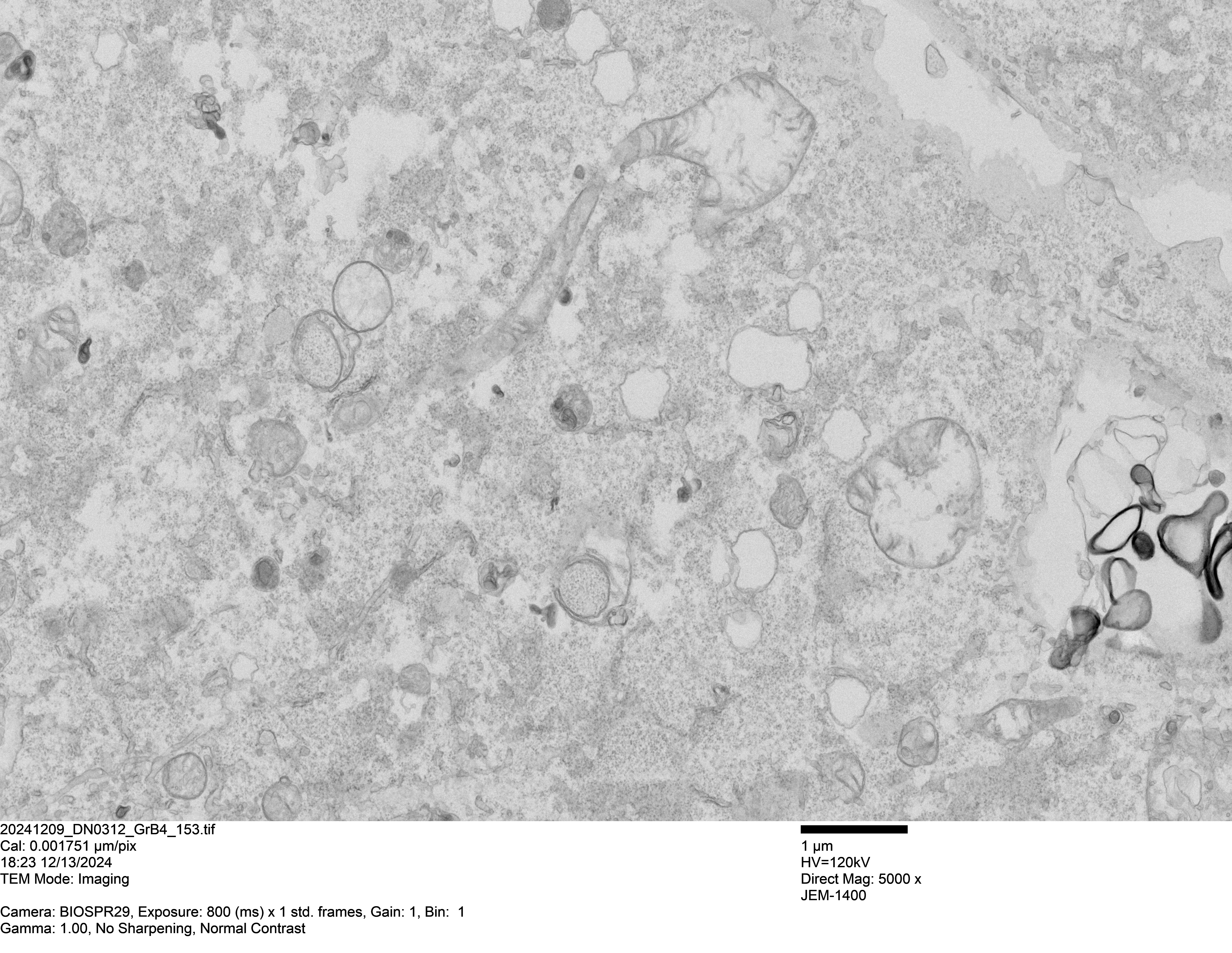

Supplement: Supplementary file 25 — Source data Fig. 3D Part 7 [file 44318_2025_604_MOESM25_ESM.zip › Fig3D_whole field EM and Confocal/Fig3D whole field EM.tif]

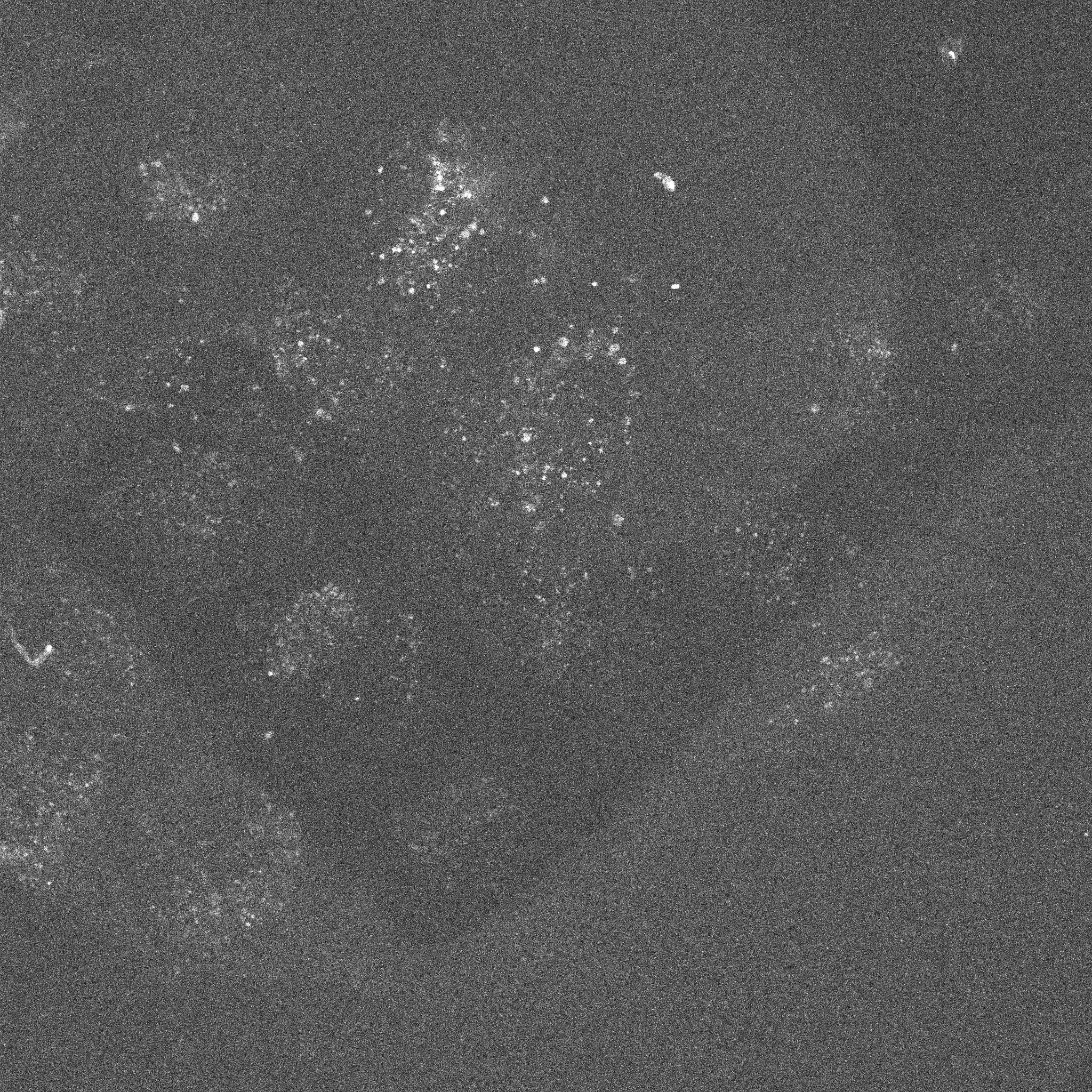

Supplement: Supplementary file 25 — Source data Fig. 3D Part 7 [file 44318_2025_604_MOESM25_ESM.zip › Fig3D_whole field EM and Confocal/Fig3D_channel 1 PINK1 YFP.tif]

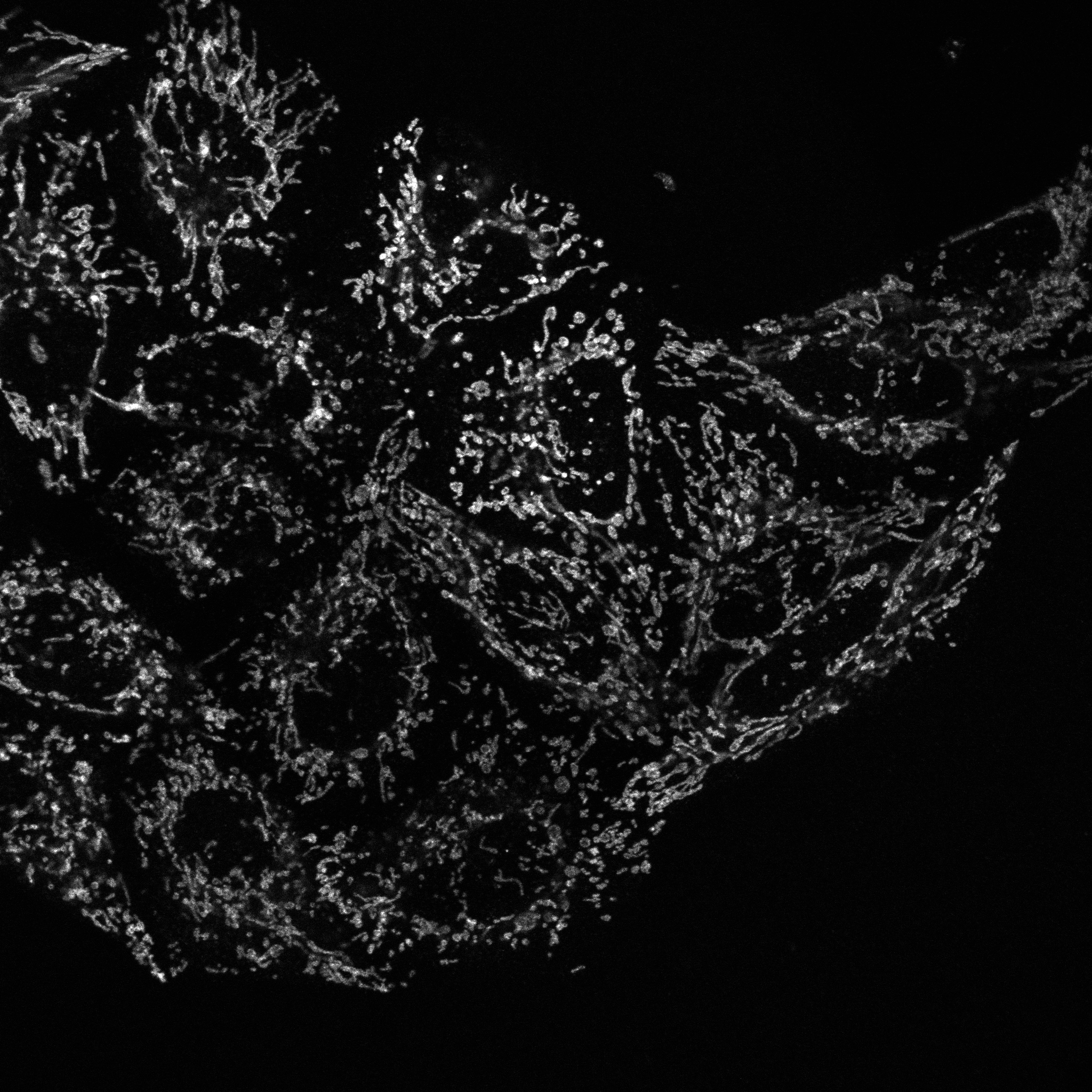

Supplement: Supplementary file 25 — Source data Fig. 3D Part 7 [file 44318_2025_604_MOESM25_ESM.zip › Fig3D_whole field EM and Confocal/Fig3D_channel 2 MTS mCherry.tif]

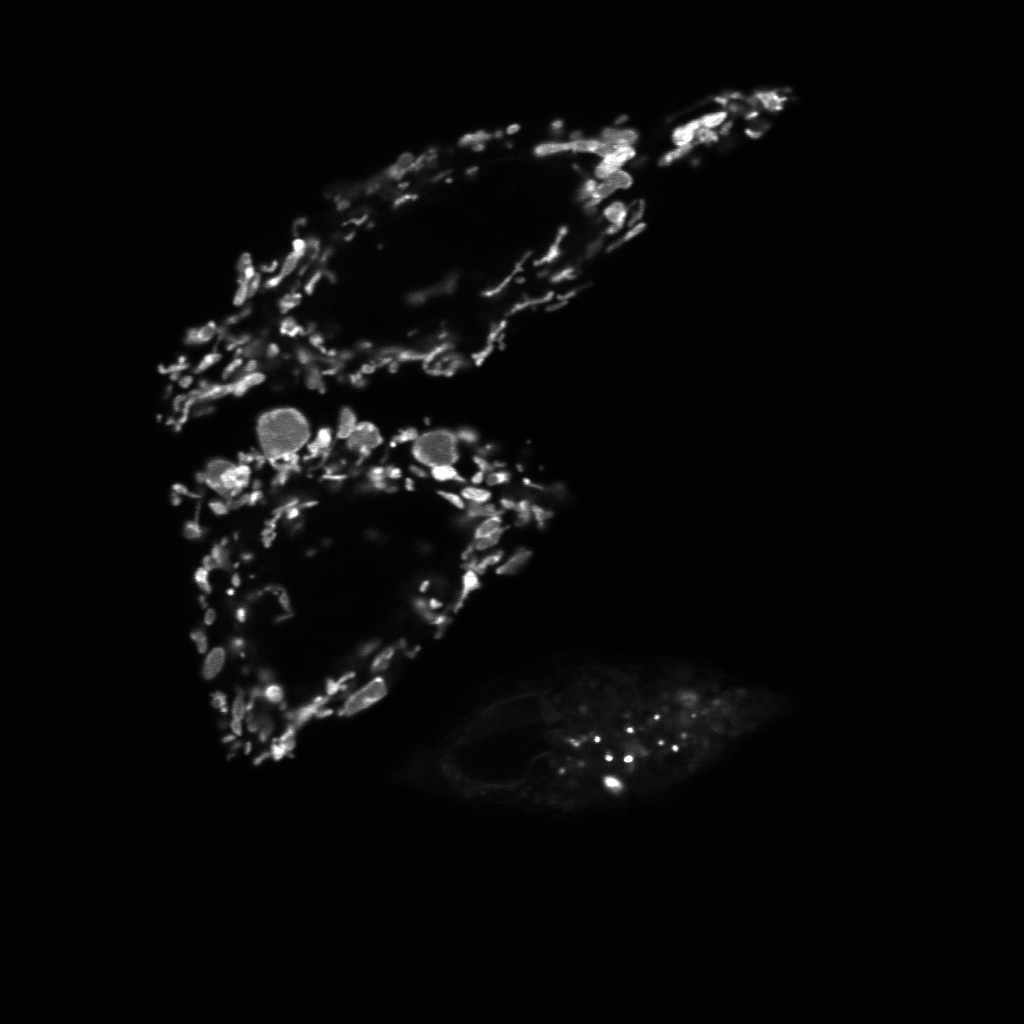

Supplement: Supplementary file 26 — Source data Fig. 4 [file 44318_2025_604_MOESM26_ESM.zip › Figure 4/4H/4H_image_PMPCB_sgRNA_channel4_MitoLiteNIR.tif]

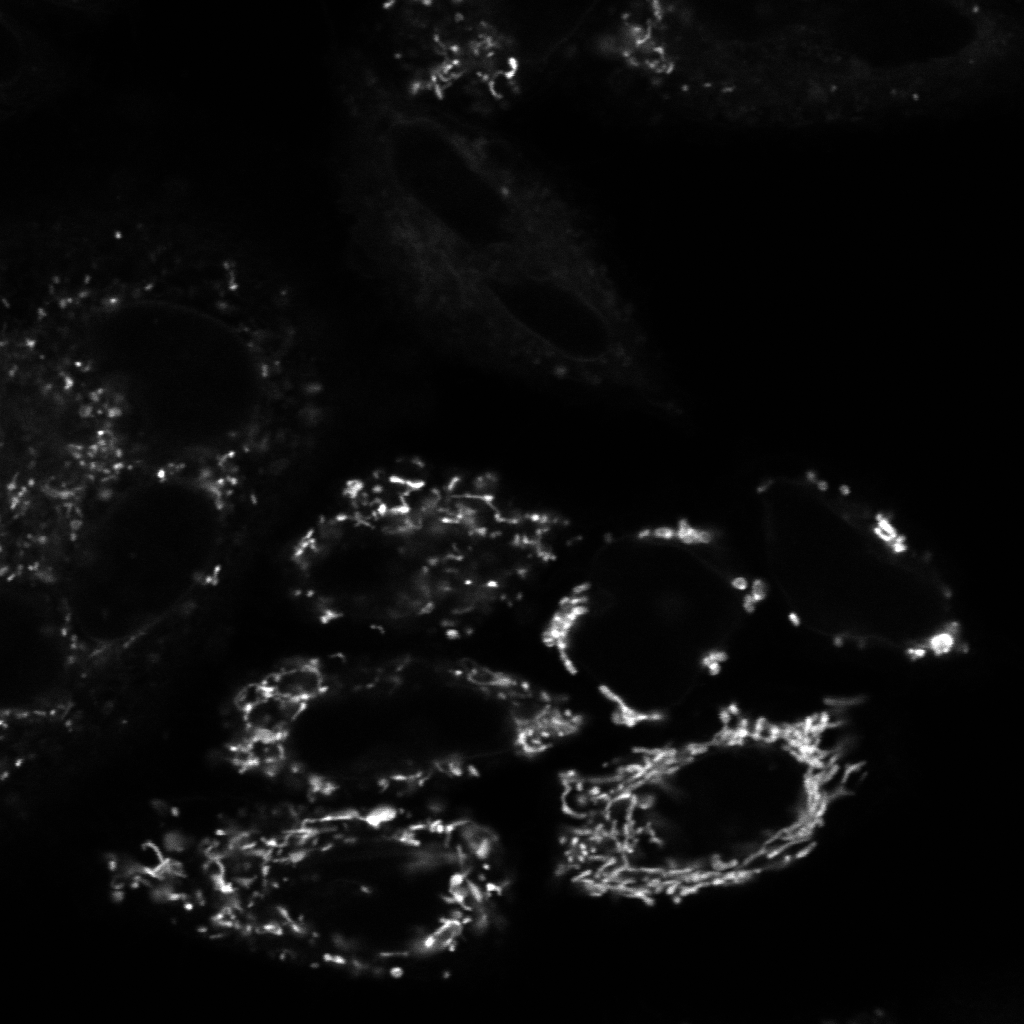

Supplement: Supplementary file 26 — Source data Fig. 4 [file 44318_2025_604_MOESM26_ESM.zip › Figure 4/4H/4H_image_TIMM23_sgRNA_channel4_MitoLiteNIR.tif]

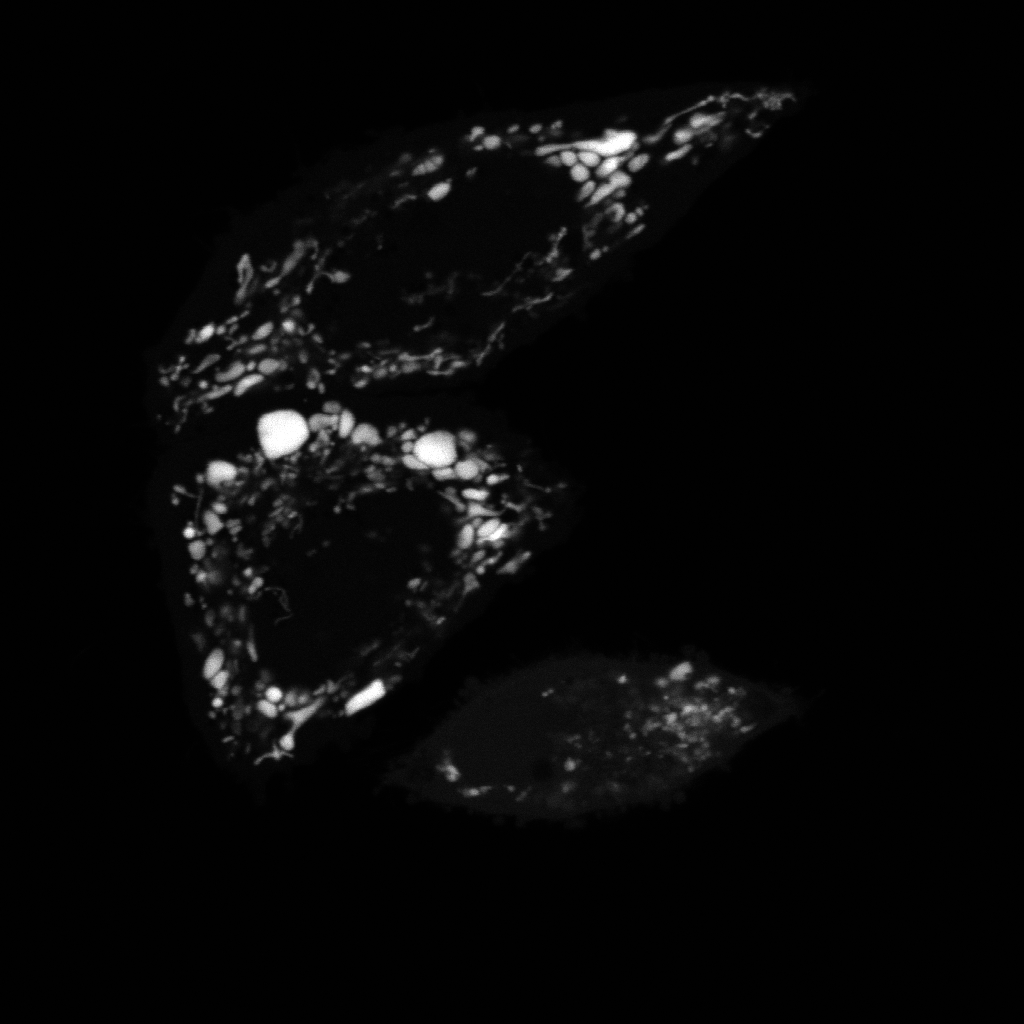

Supplement: Supplementary file 26 — Source data Fig. 4 [file 44318_2025_604_MOESM26_ESM.zip › Figure 4/4H/4H_image_PMPCB_sgRNA_channel3_MTSmCherry.tif]

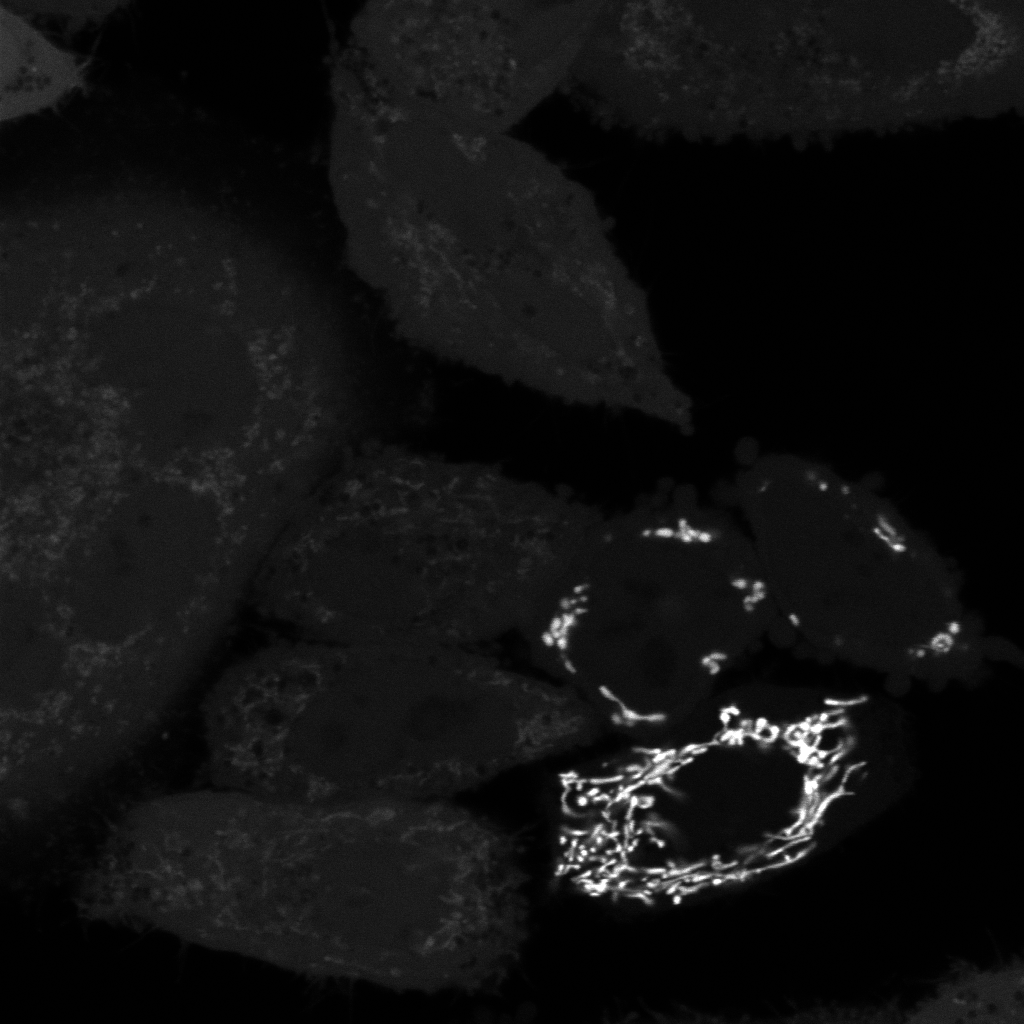

Supplement: Supplementary file 26 — Source data Fig. 4 [file 44318_2025_604_MOESM26_ESM.zip › Figure 4/4H/4H_image_TIMM23_sgRNA_channel3_MTSmCherry.tif]

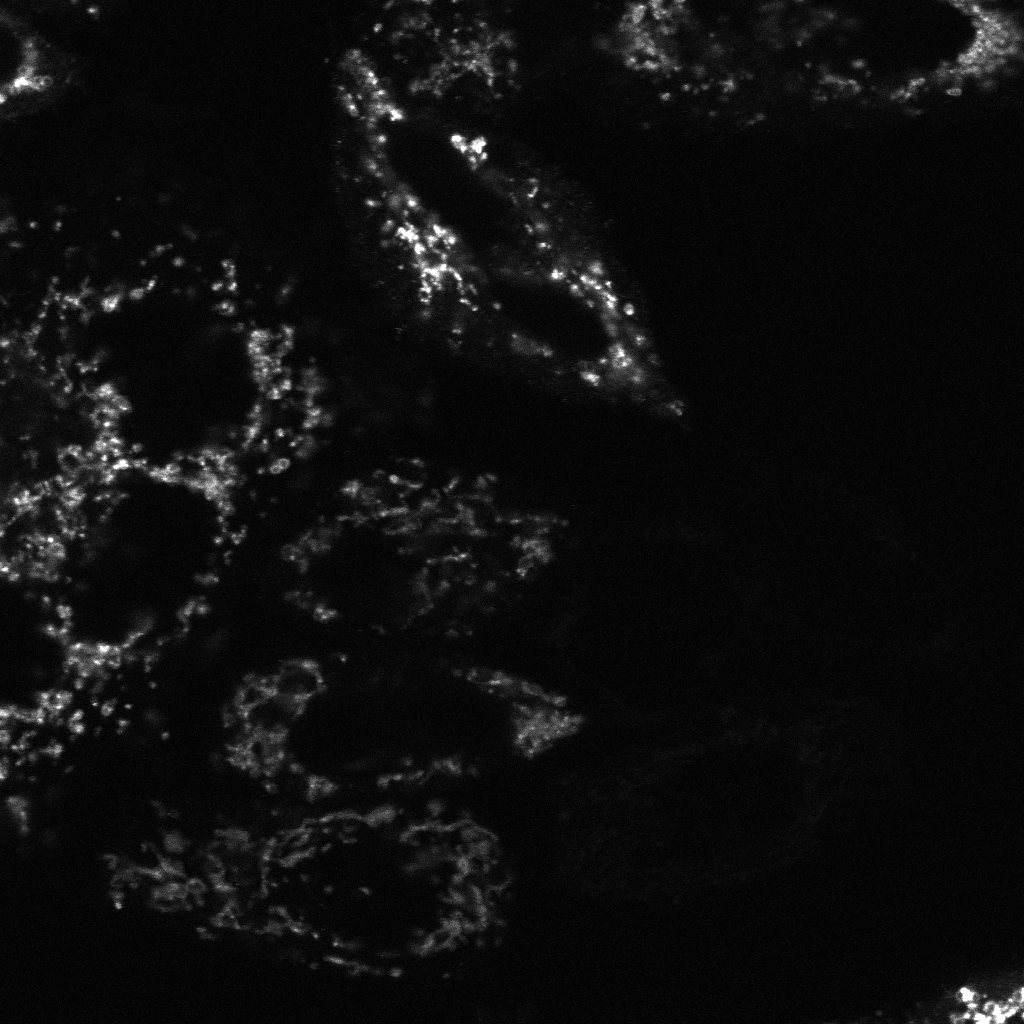

Supplement: Supplementary file 26 — Source data Fig. 4 [file 44318_2025_604_MOESM26_ESM.zip › Figure 4/4H/4H_image_TIMM23_sgRNA_channel2_PINK1YFP.tif]

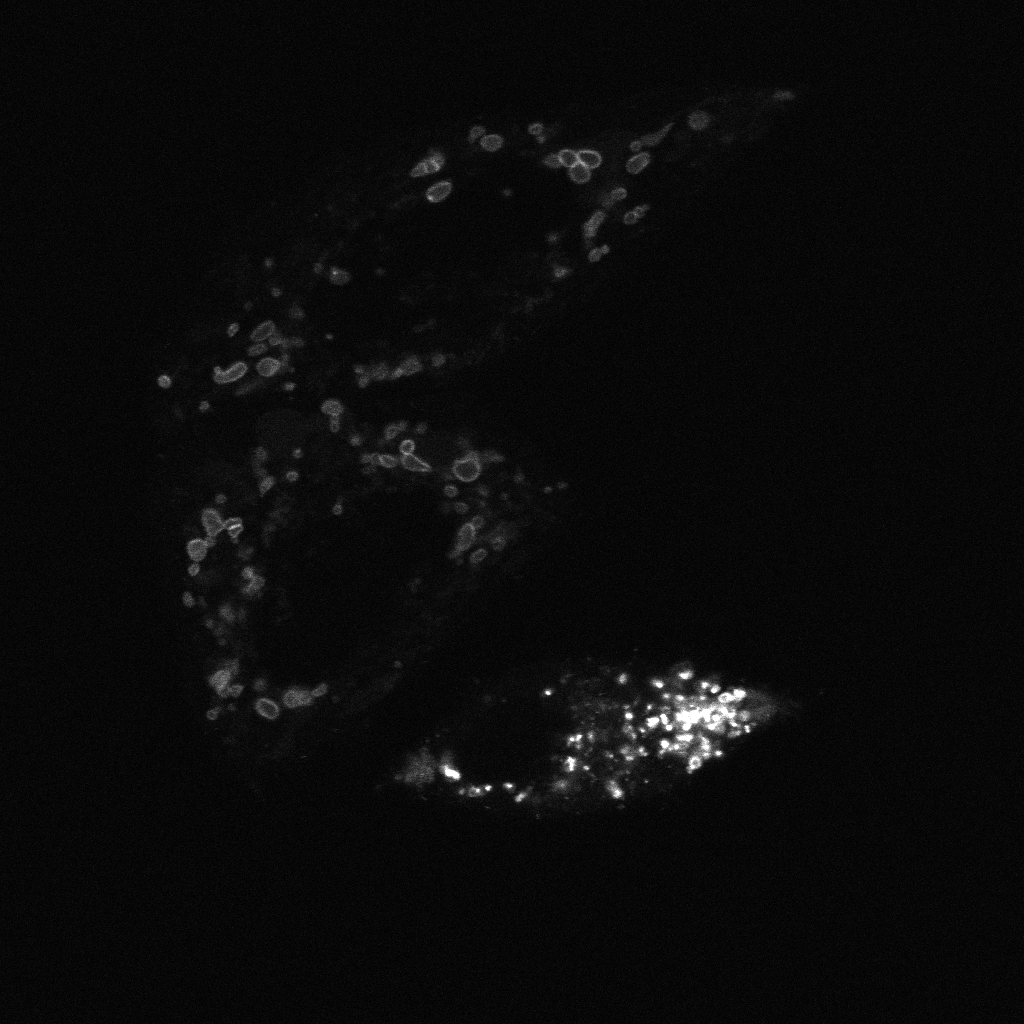

Supplement: Supplementary file 26 — Source data Fig. 4 [file 44318_2025_604_MOESM26_ESM.zip › Figure 4/4H/4H_image_PMPCB_sgRNA_channel2_PINK1YFP.tif]

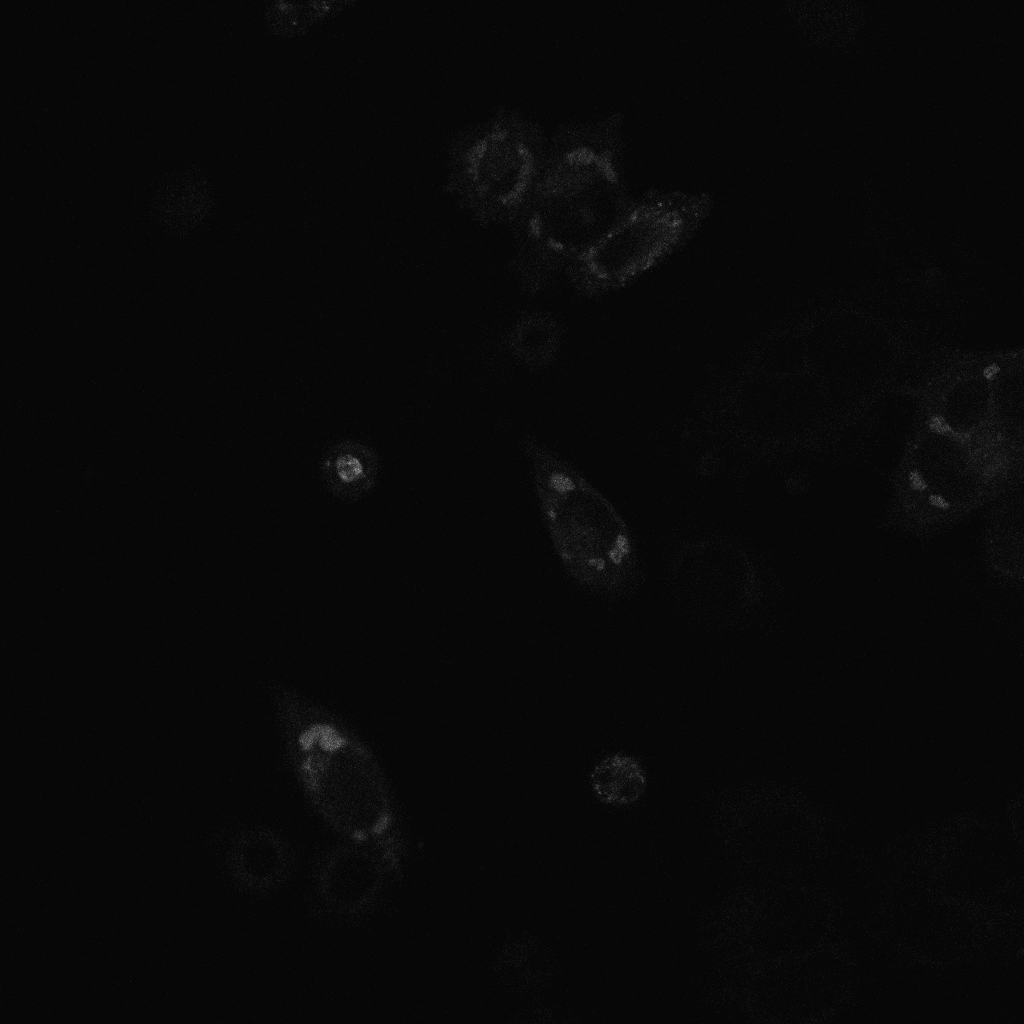

Supplement: Supplementary file 27 — Source data Fig. 5 [file 44318_2025_604_MOESM27_ESM.zip › Figure 5/5D/5D_image_40x stack_Maximum intensity projection.czi - C=1_PINK1 YFP.tif]

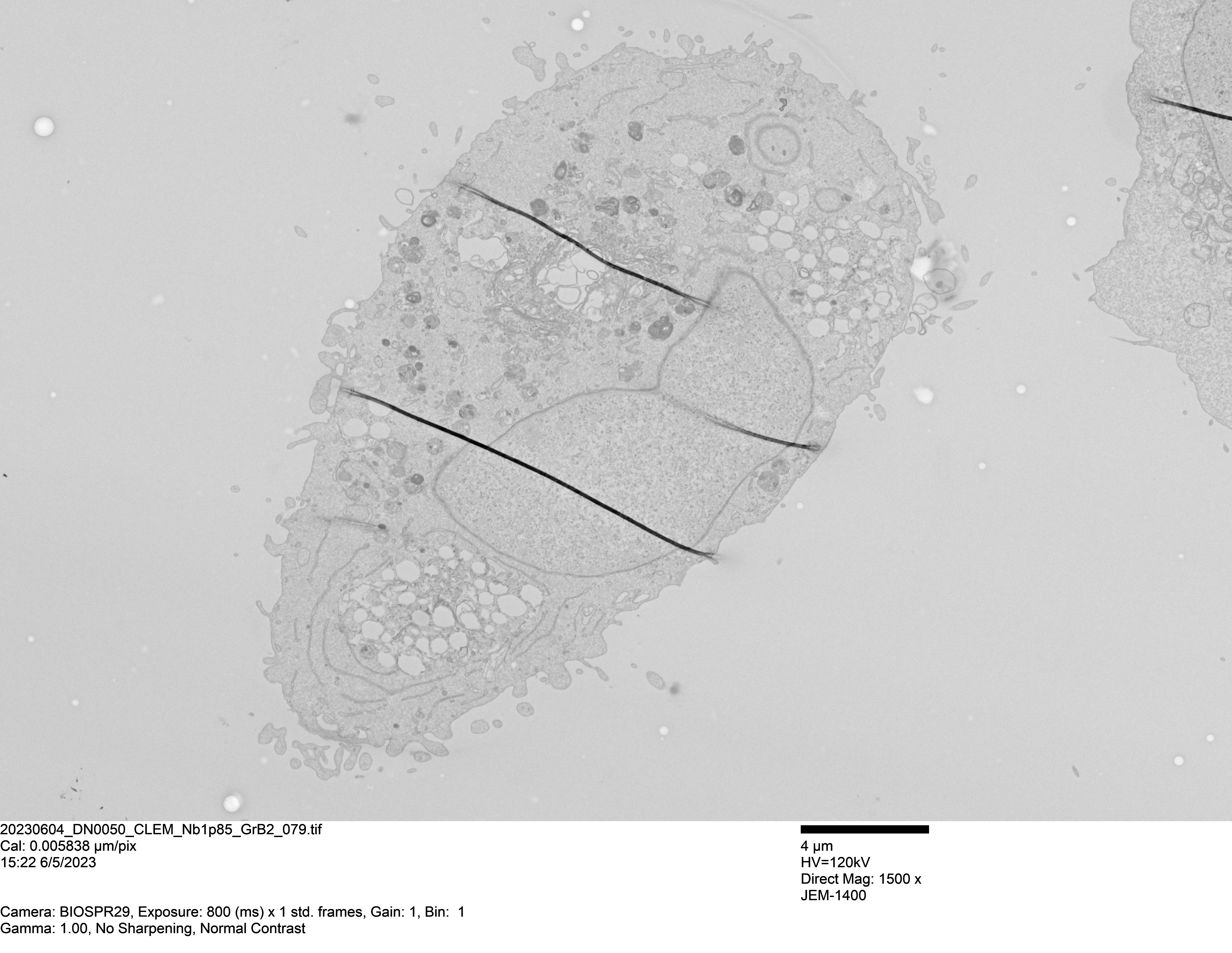

Supplement: Supplementary file 27 — Source data Fig. 5 [file 44318_2025_604_MOESM27_ESM.zip › Figure 5/5D/5D_image_EM_1500X.tif]

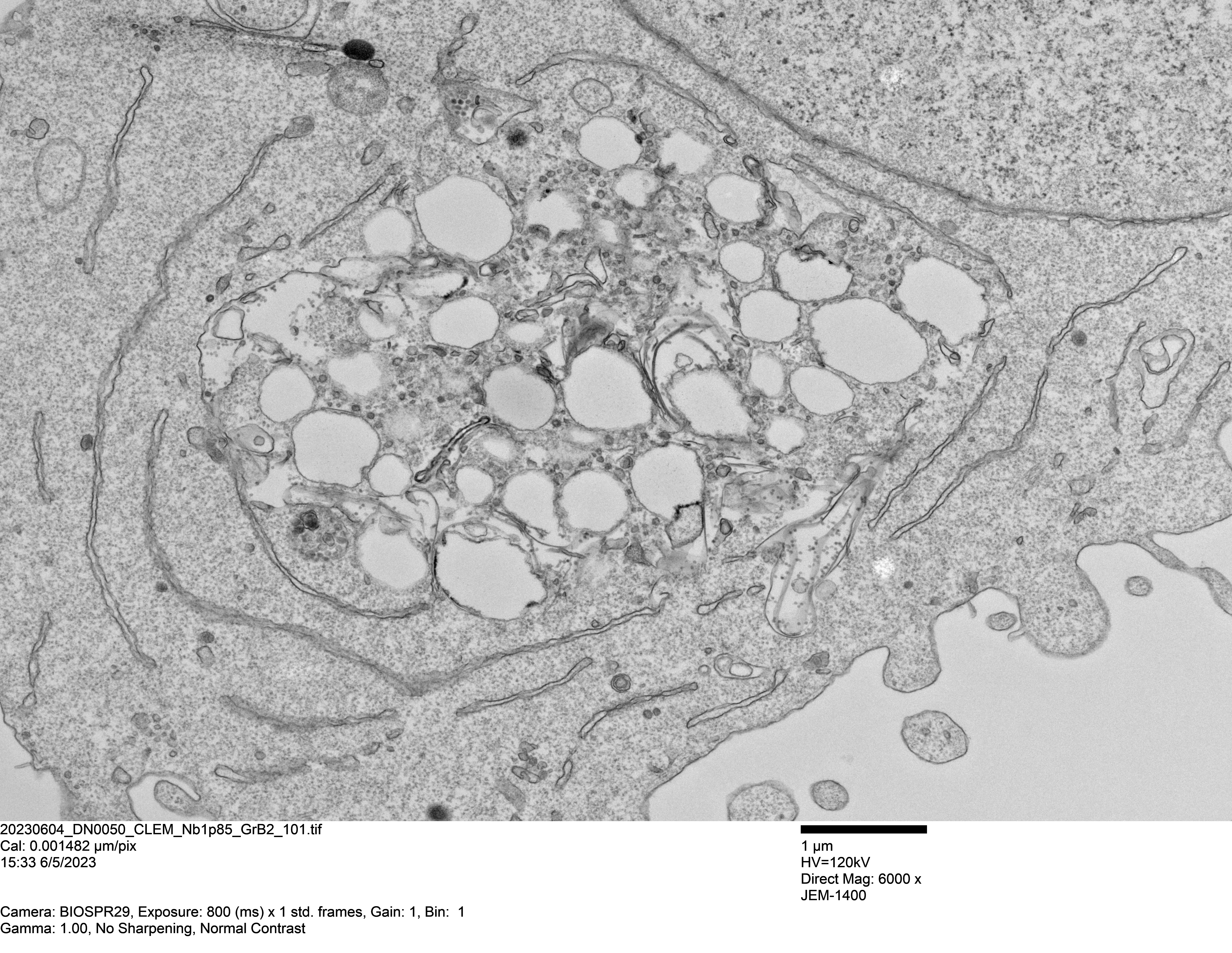

Supplement: Supplementary file 27 — Source data Fig. 5 [file 44318_2025_604_MOESM27_ESM.zip › Figure 5/5D/5D_image_EM_6000X.tif]

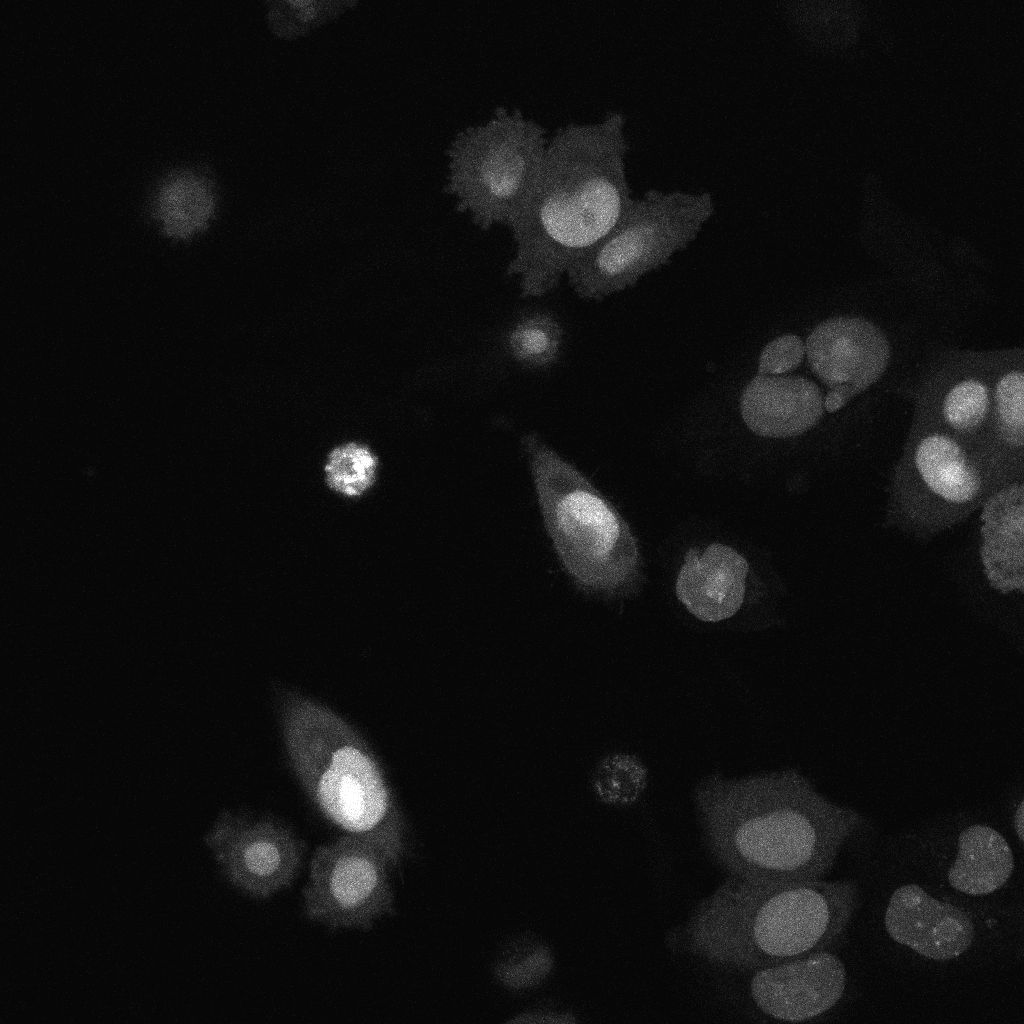

Supplement: Supplementary file 27 — Source data Fig. 5 [file 44318_2025_604_MOESM27_ESM.zip › Figure 5/5D/5D_image_40x stack_Maximum intensity projection.czi - C=2_TOMM22 sgRNA.tif]

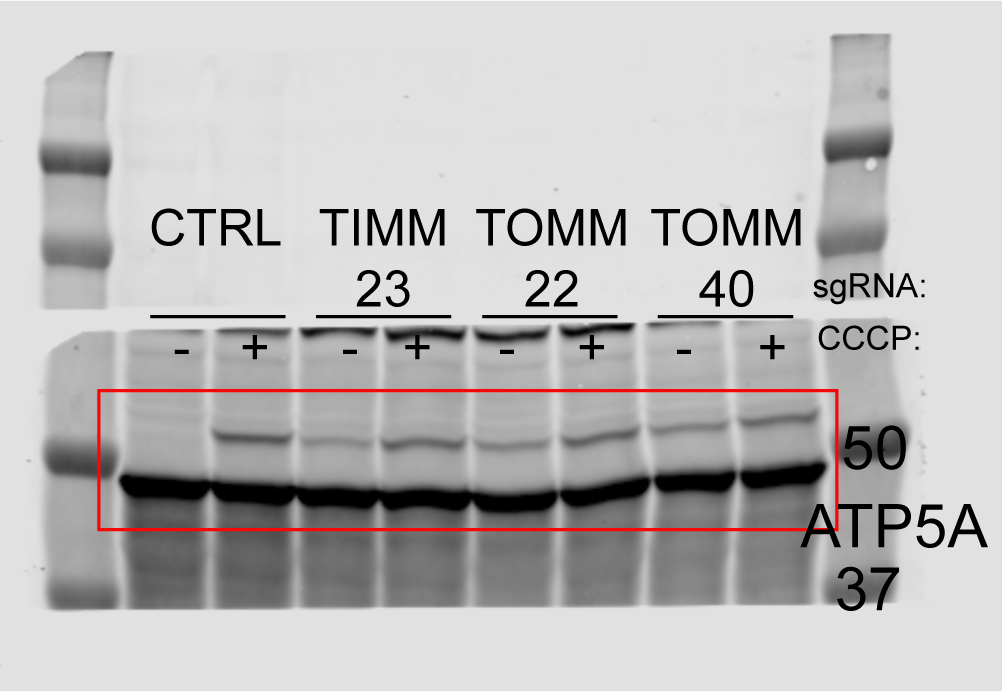

Supplement: Supplementary file 27 — Source data Fig. 5 [file 44318_2025_604_MOESM27_ESM.zip › Figure 5/5H/5H right blot_western atp5a.tif]

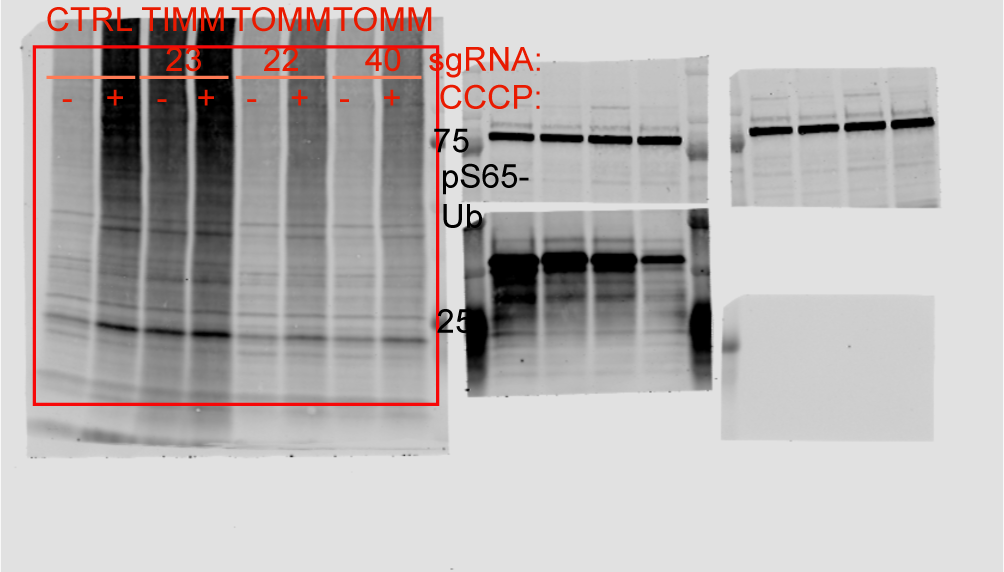

Supplement: Supplementary file 27 — Source data Fig. 5 [file 44318_2025_604_MOESM27_ESM.zip › Figure 5/5H/5H left blot_western pS65 Ub.tif]

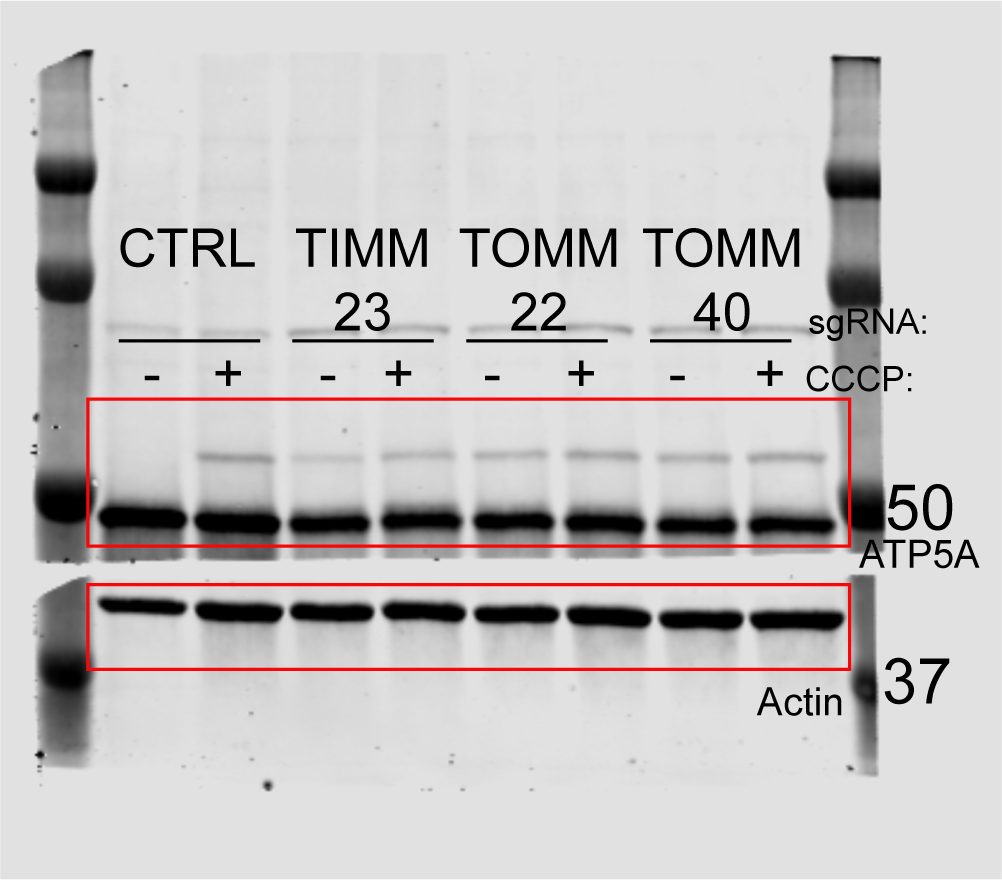

Supplement: Supplementary file 27 — Source data Fig. 5 [file 44318_2025_604_MOESM27_ESM.zip › Figure 5/5H/5H left blot_western atp5a and actin for pink1 and atp5a.tif]

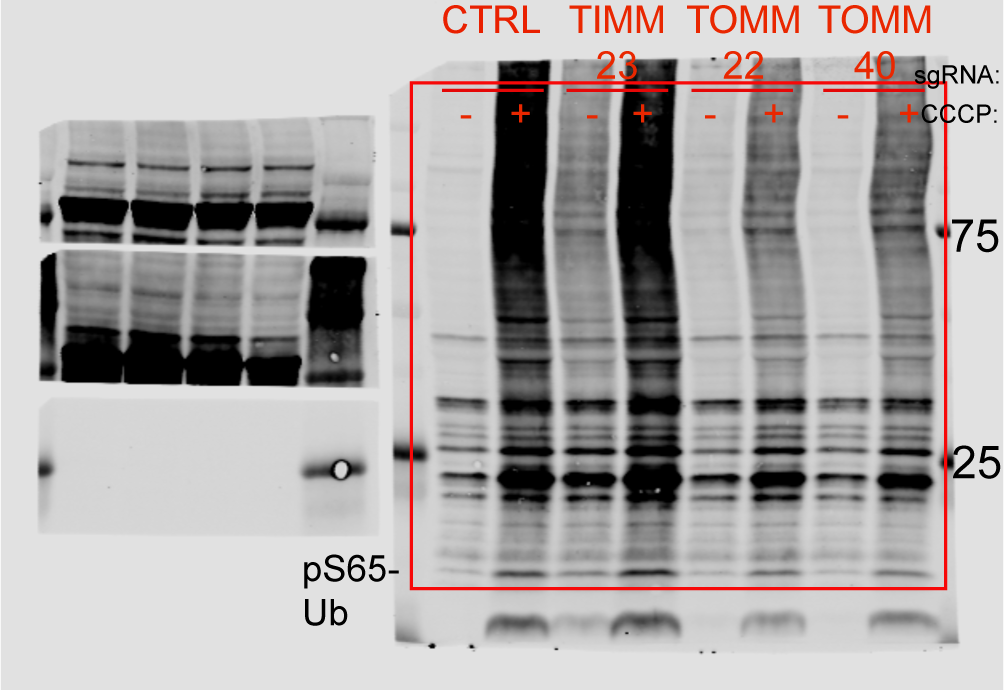

Supplement: Supplementary file 27 — Source data Fig. 5 [file 44318_2025_604_MOESM27_ESM.zip › Figure 5/5H/5H right blot_western pS65 Ub_right blot endog.tif]

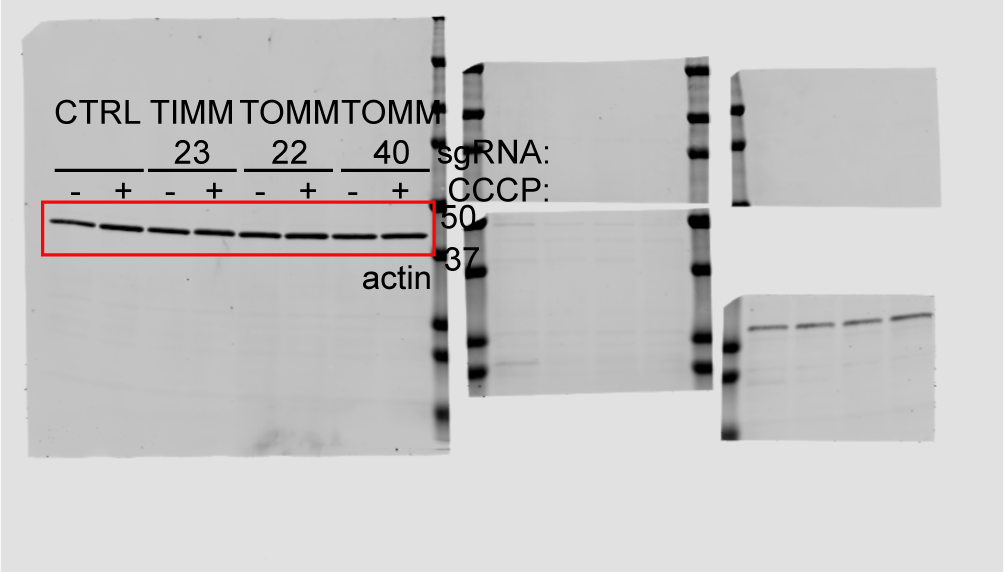

Supplement: Supplementary file 27 — Source data Fig. 5 [file 44318_2025_604_MOESM27_ESM.zip › Figure 5/5H/5H left blot_western actin for pS65 Ub.tif]

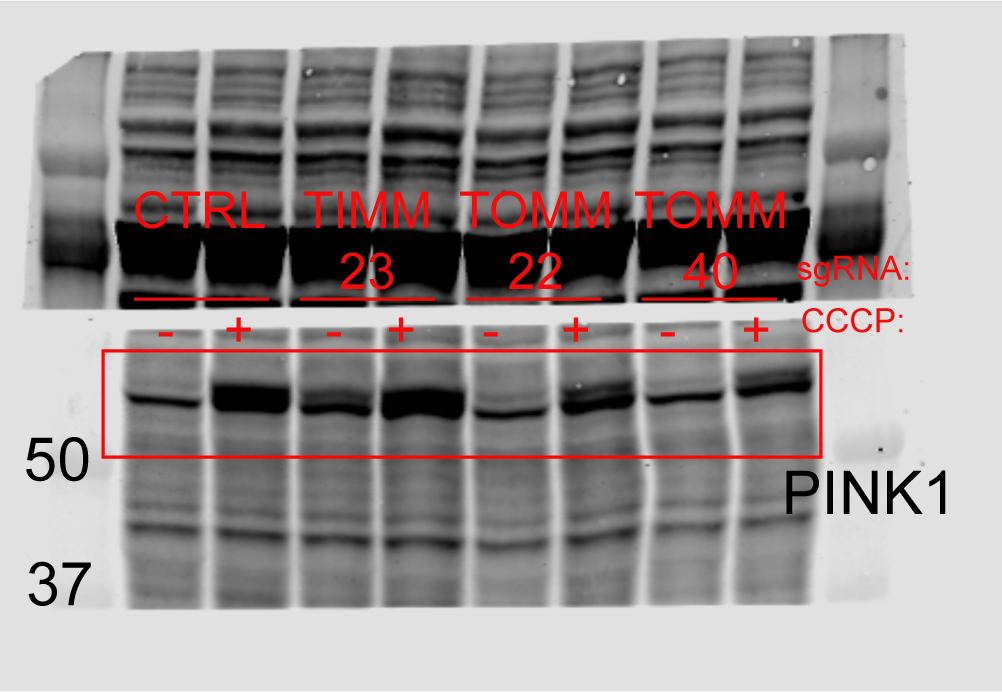

Supplement: Supplementary file 27 — Source data Fig. 5 [file 44318_2025_604_MOESM27_ESM.zip › Figure 5/5H/5H right blot_western pink1.tif]

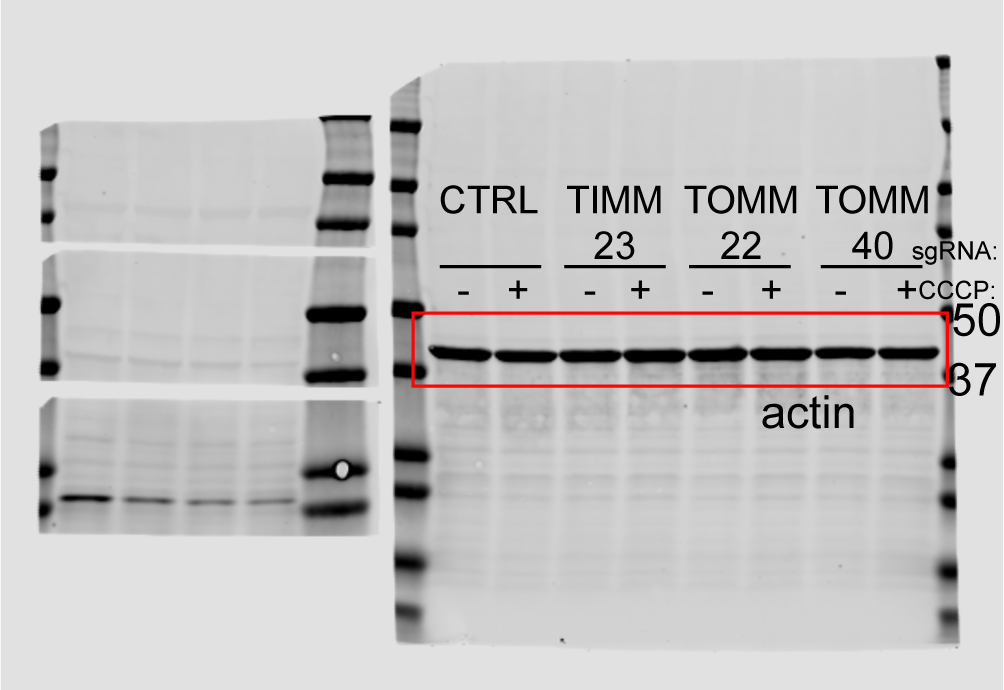

Supplement: Supplementary file 27 — Source data Fig. 5 [file 44318_2025_604_MOESM27_ESM.zip › Figure 5/5H/5H right blot_western actin for pS65 Ub.tif]

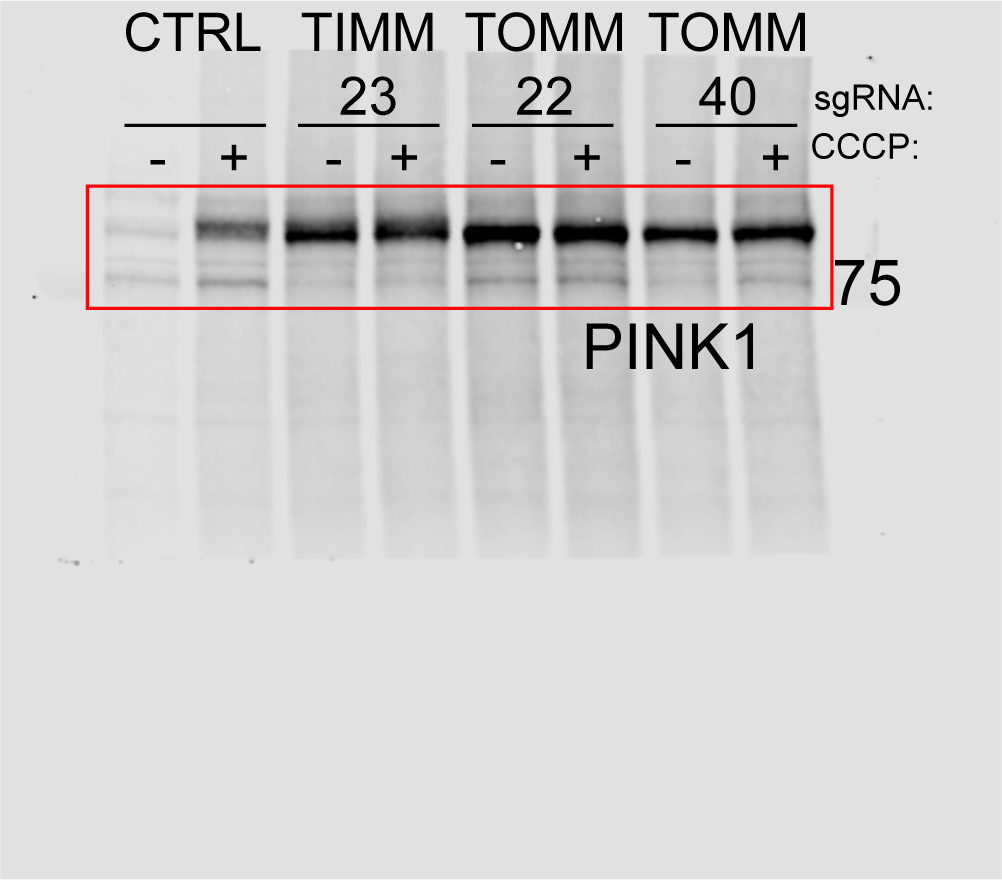

Supplement: Supplementary file 27 — Source data Fig. 5 [file 44318_2025_604_MOESM27_ESM.zip › Figure 5/5H/5H left blot_western pink1.tif]

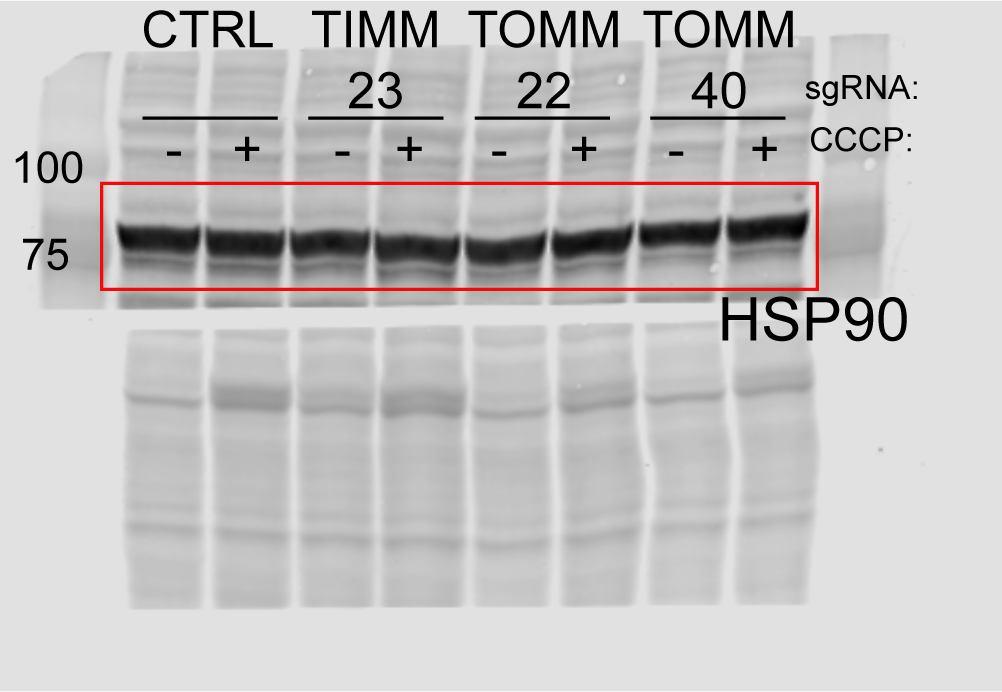

Supplement: Supplementary file 27 — Source data Fig. 5 [file 44318_2025_604_MOESM27_ESM.zip › Figure 5/5H/5H right blot_western hsp90 for pink1 and atp5a.tif]

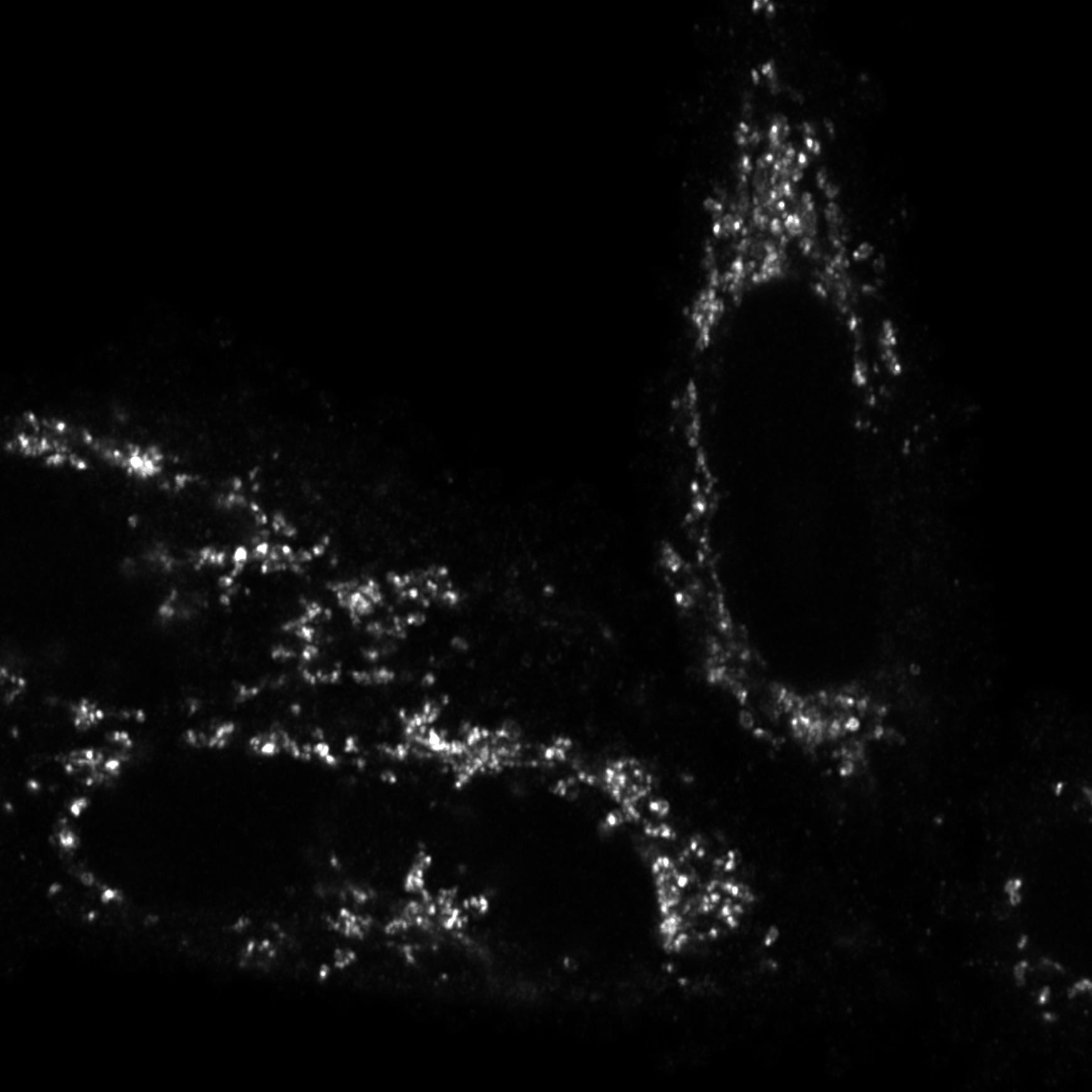

Supplement: Supplementary file 27 — Source data Fig. 5 [file 44318_2025_604_MOESM27_ESM.zip › Figure 5/5C/5C_image_TIMM23KOpool_MIP_merge.tif]

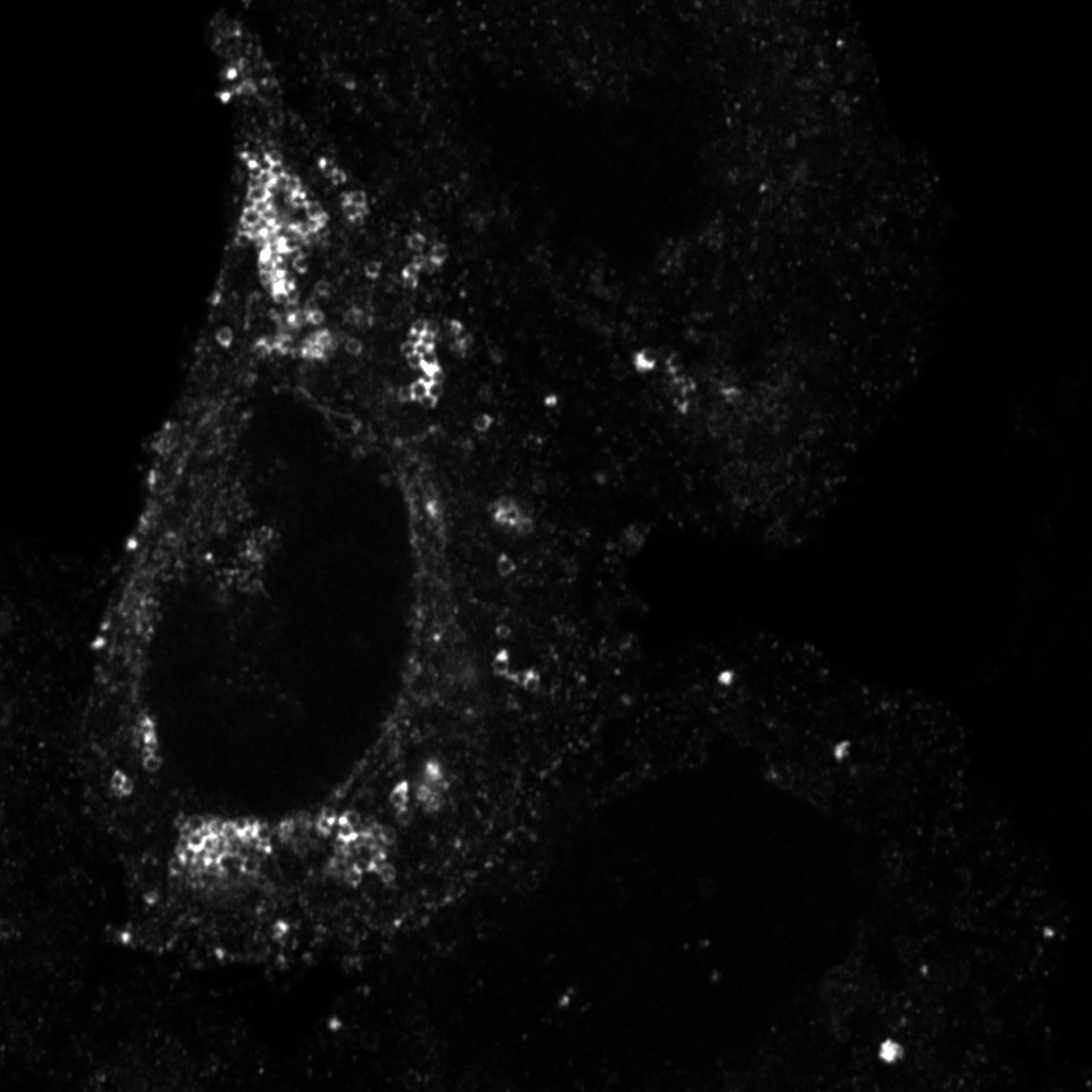

Supplement: Supplementary file 27 — Source data Fig. 5 [file 44318_2025_604_MOESM27_ESM.zip › Figure 5/5C/5C_image_TOMM22KOpool_MIP_merge.tif]

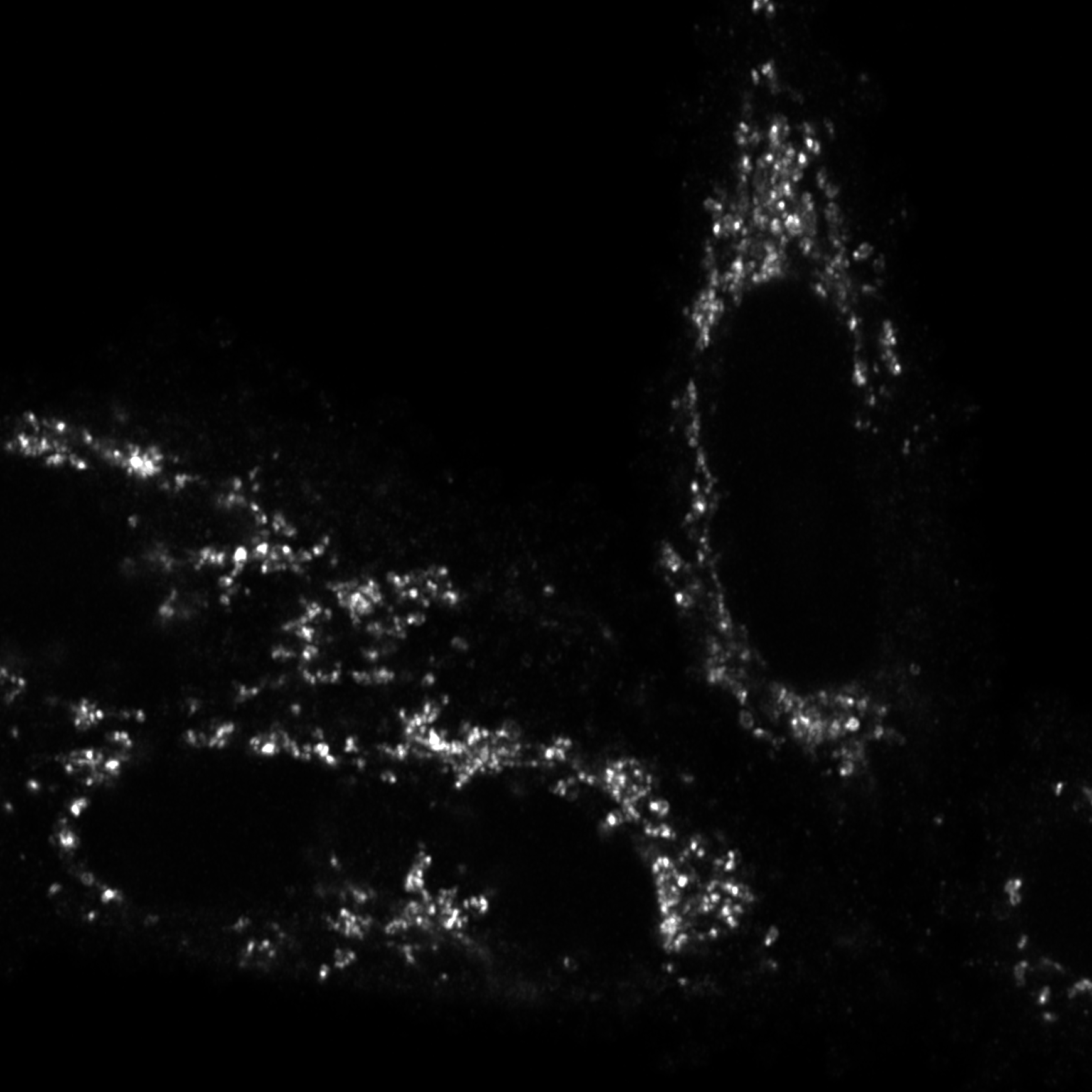

Supplement: Supplementary file 27 — Source data Fig. 5 [file 44318_2025_604_MOESM27_ESM.zip › Figure 5/5C/5C_image_TIMM23KOpool_MIP_PINK1YFP.tif]

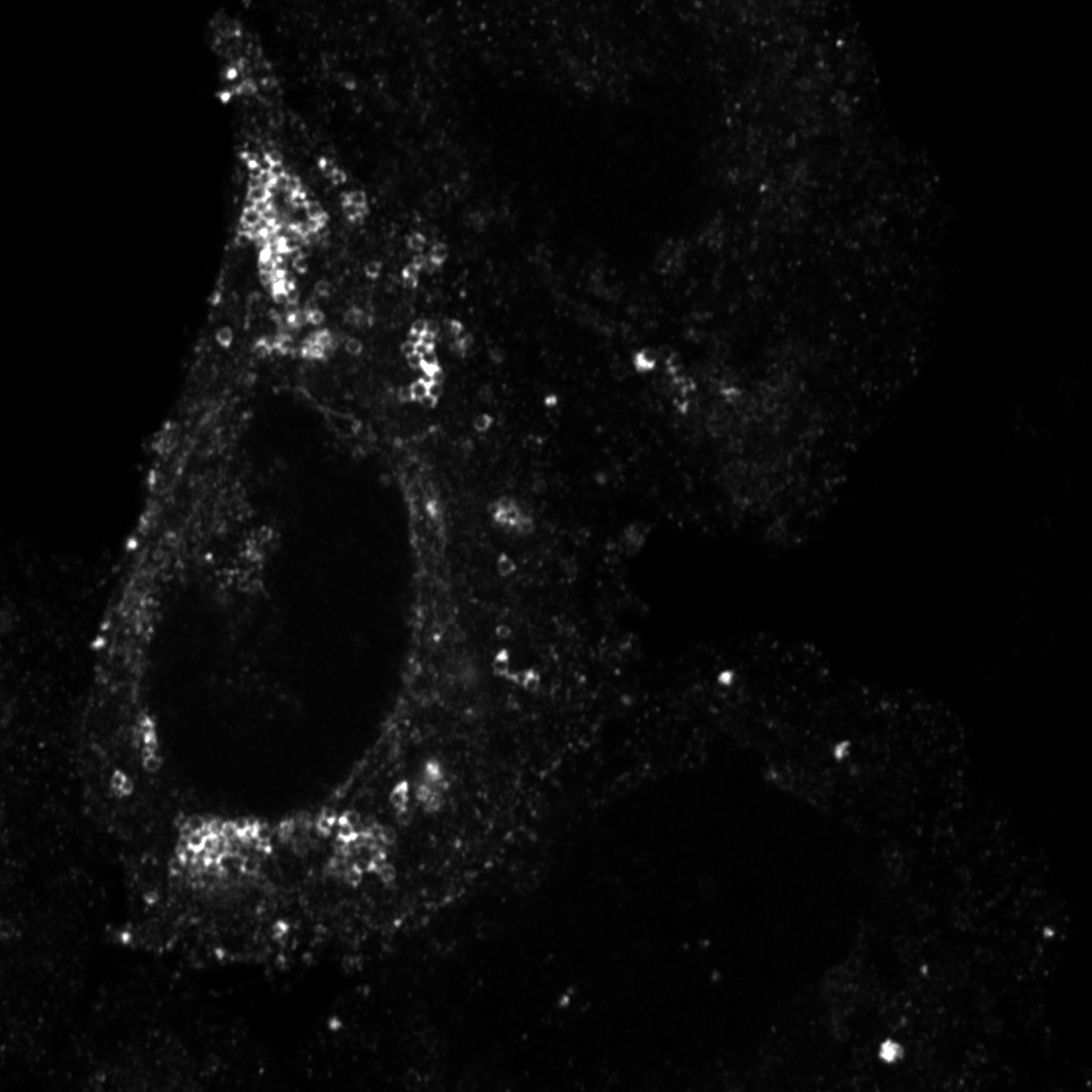

Supplement: Supplementary file 27 — Source data Fig. 5 [file 44318_2025_604_MOESM27_ESM.zip › Figure 5/5C/5C_image_TOMM22KOpool_MIP_PINK1YFP.tif]

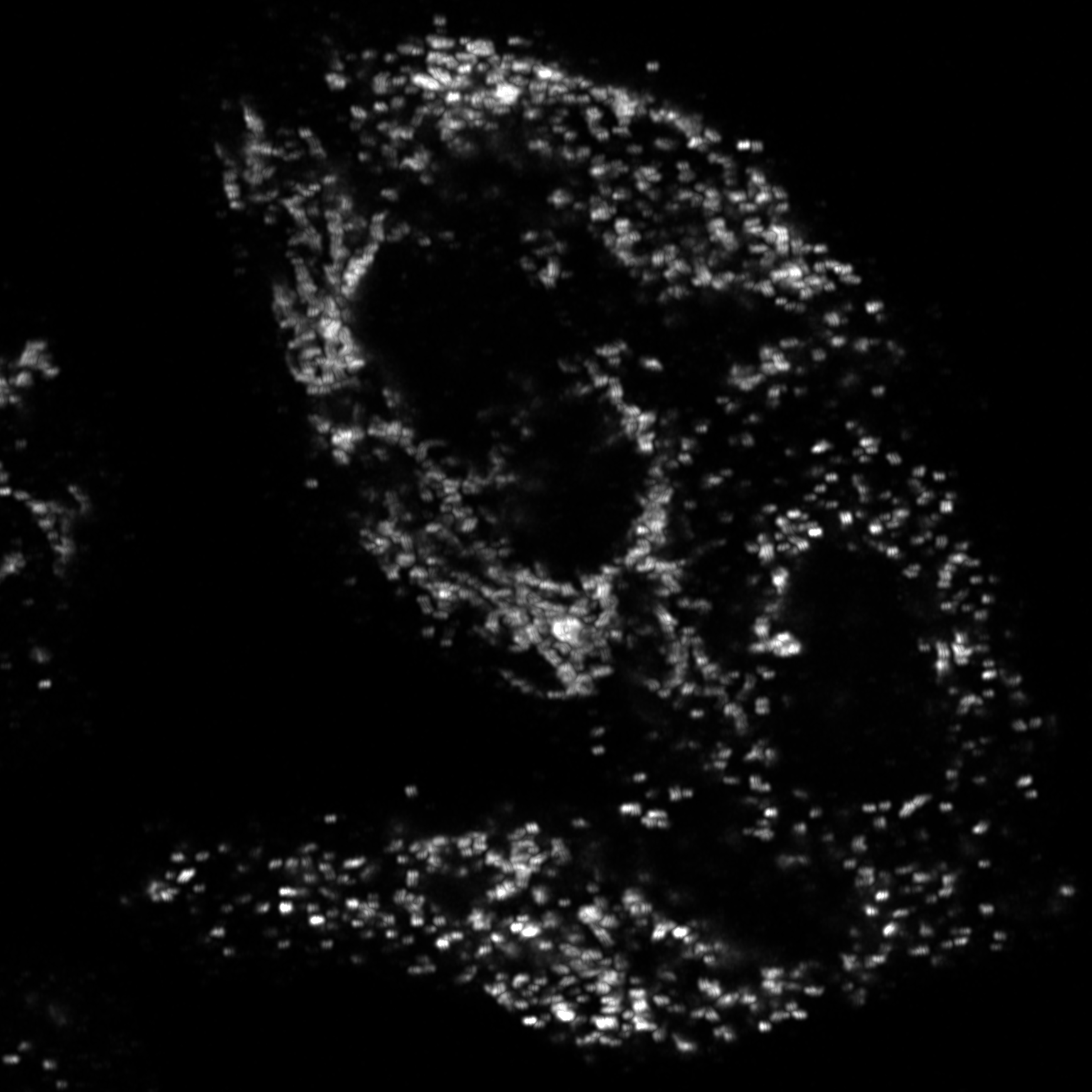

Supplement: Supplementary file 27 — Source data Fig. 5 [file 44318_2025_604_MOESM27_ESM.zip › Figure 5/5C/5C_image_TOMM40KOpool_MIP_TOMM70abAlexa647.tif]

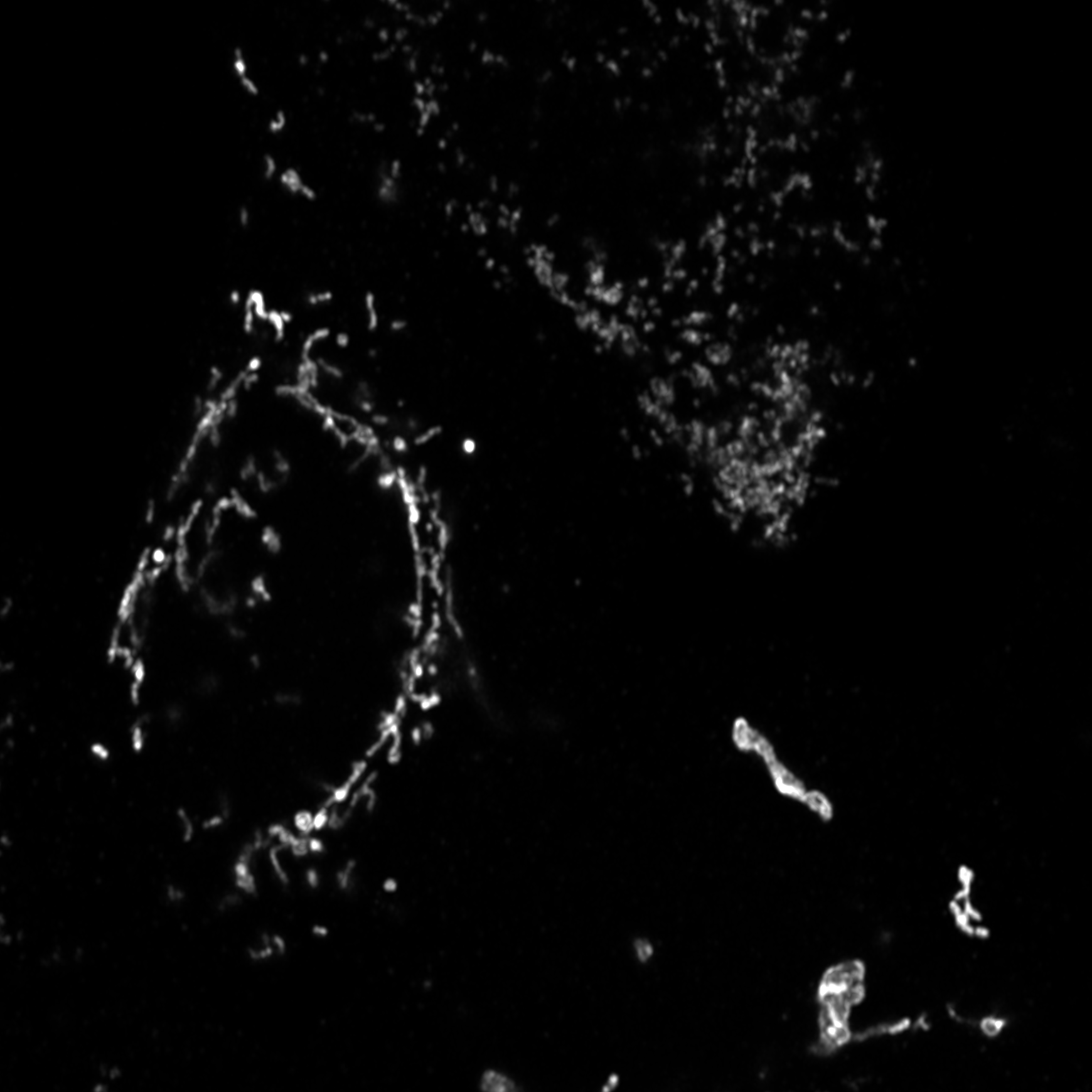

Supplement: Supplementary file 27 — Source data Fig. 5 [file 44318_2025_604_MOESM27_ESM.zip › Figure 5/5C/5C_image_TOMM22KOpool_MIP_TOMM7abAlexa647.tif]

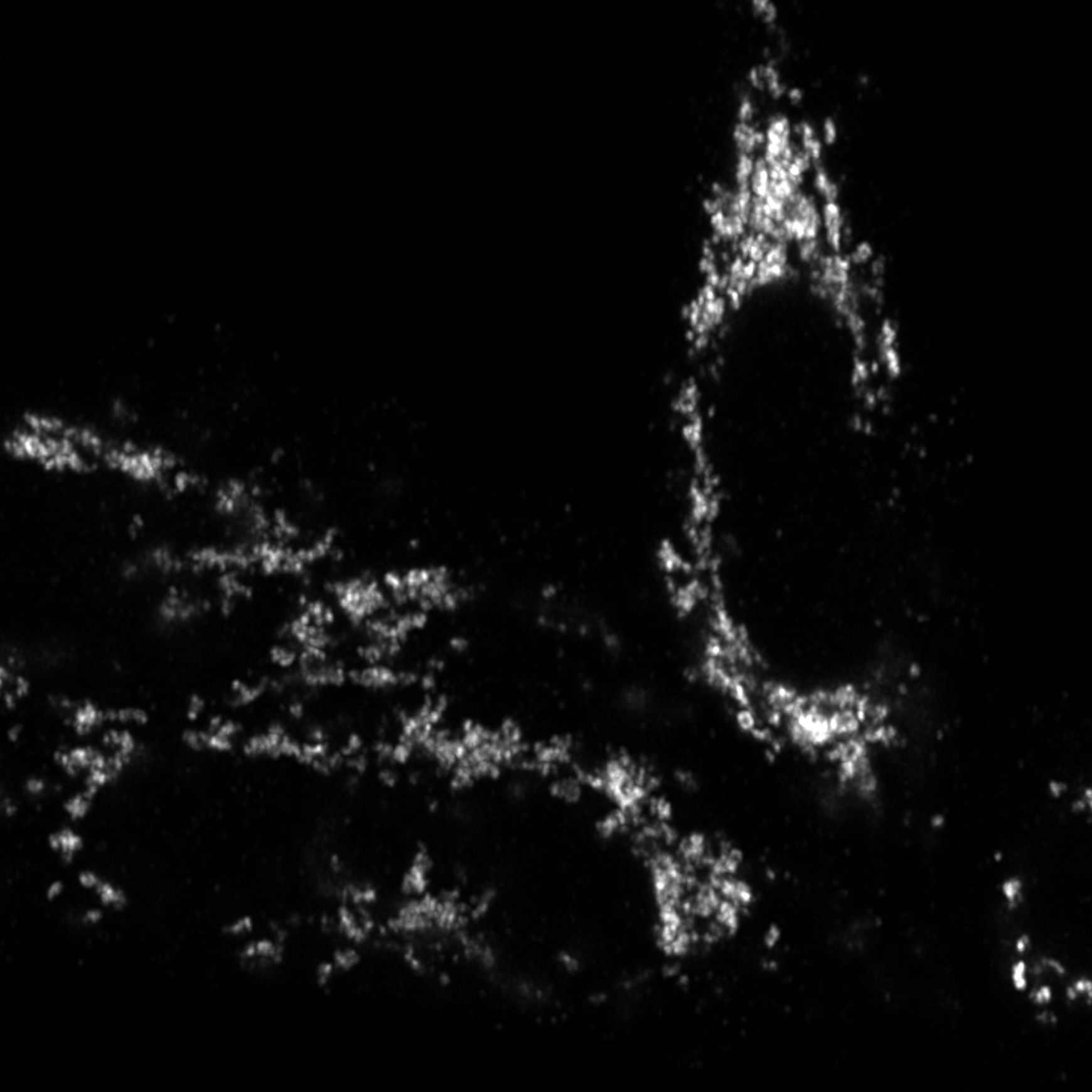

Supplement: Supplementary file 27 — Source data Fig. 5 [file 44318_2025_604_MOESM27_ESM.zip › Figure 5/5C/5C_image_TIMM23KOpool_MIP_TOMM70abAlexa647.tif]

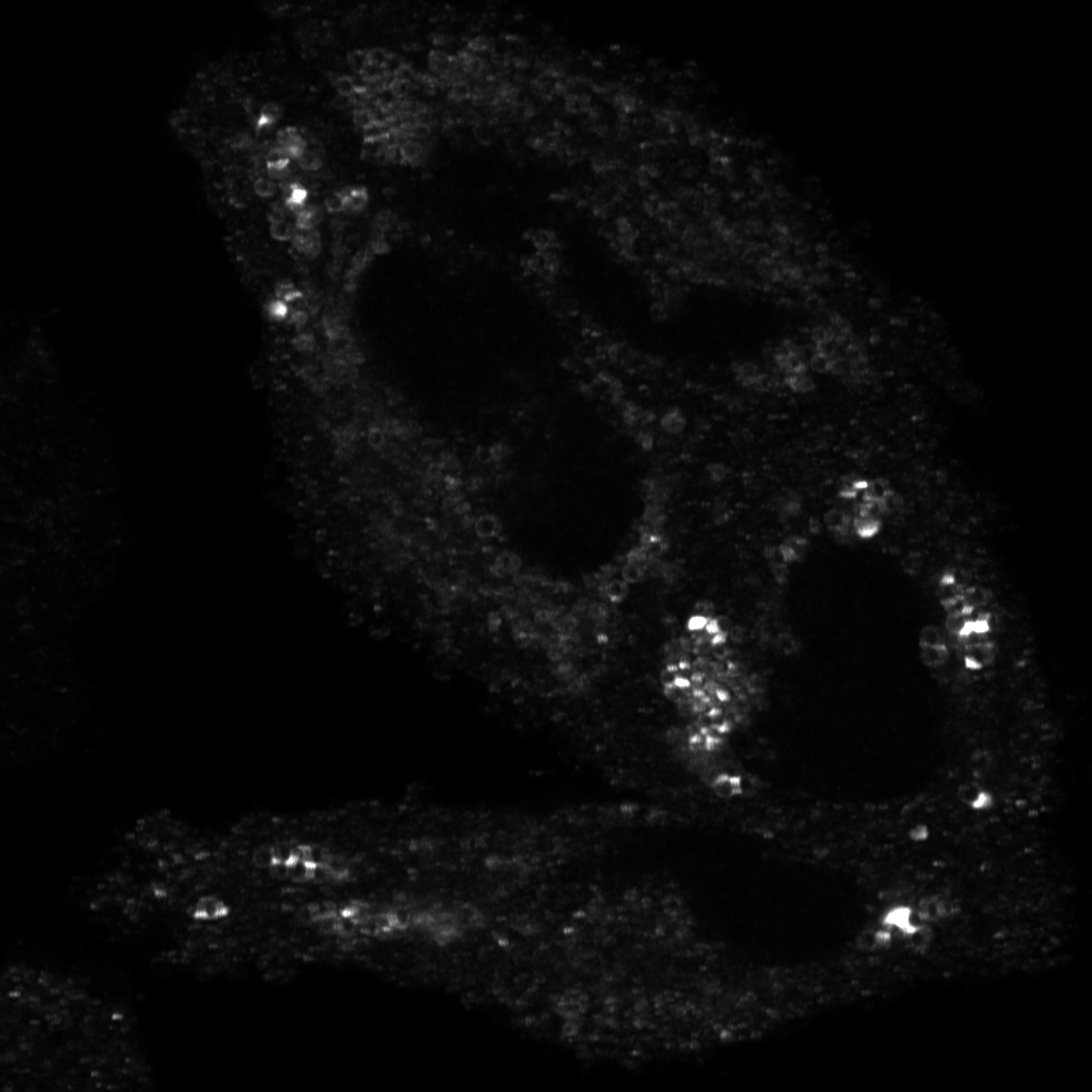

Supplement: Supplementary file 27 — Source data Fig. 5 [file 44318_2025_604_MOESM27_ESM.zip › Figure 5/5C/5C_image_TOMM40KOpool_MIP_PINK1YFP.tif]

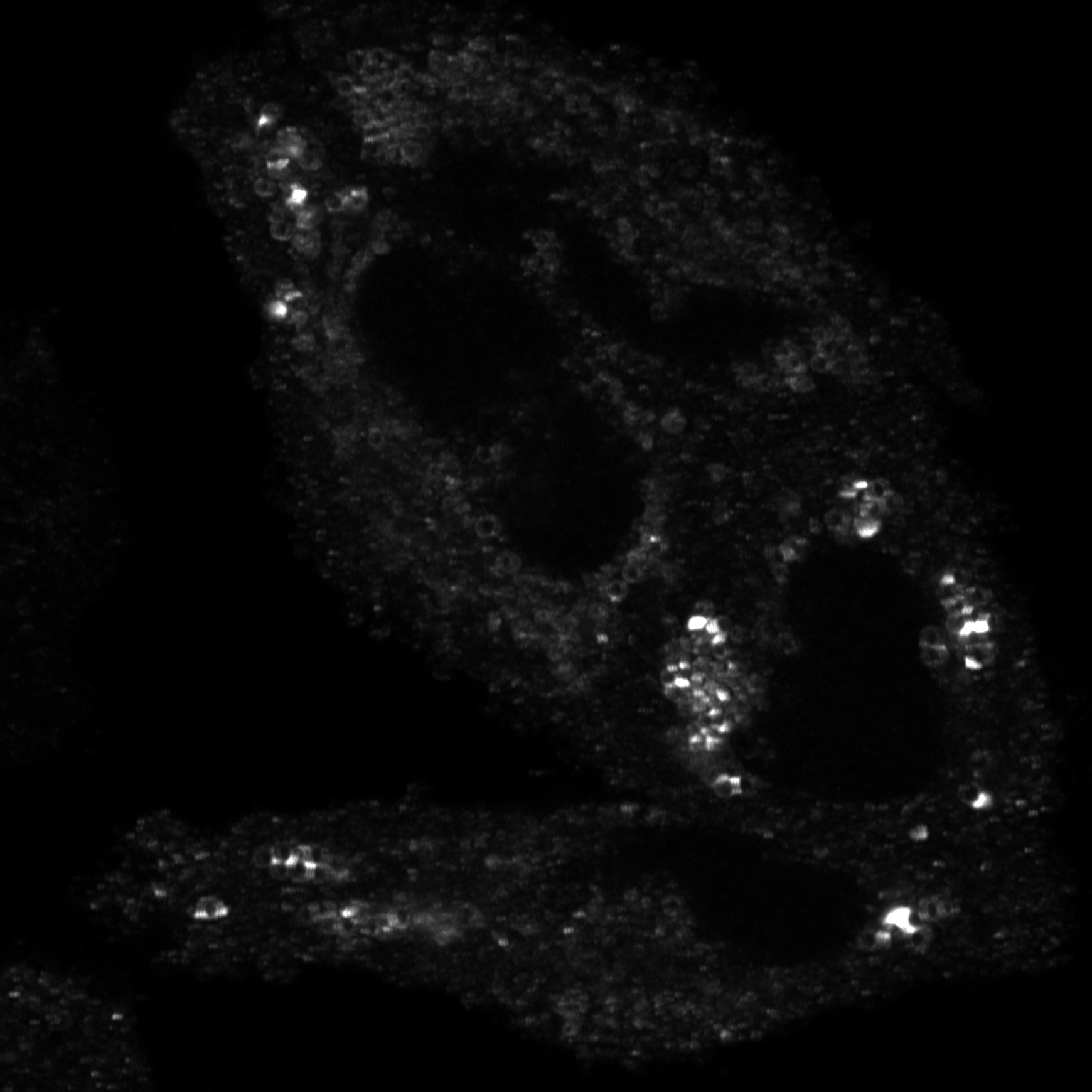

Supplement: Supplementary file 27 — Source data Fig. 5 [file 44318_2025_604_MOESM27_ESM.zip › Figure 5/5C/5C_image_TOMM40KOpool_MIP_merge.tif]

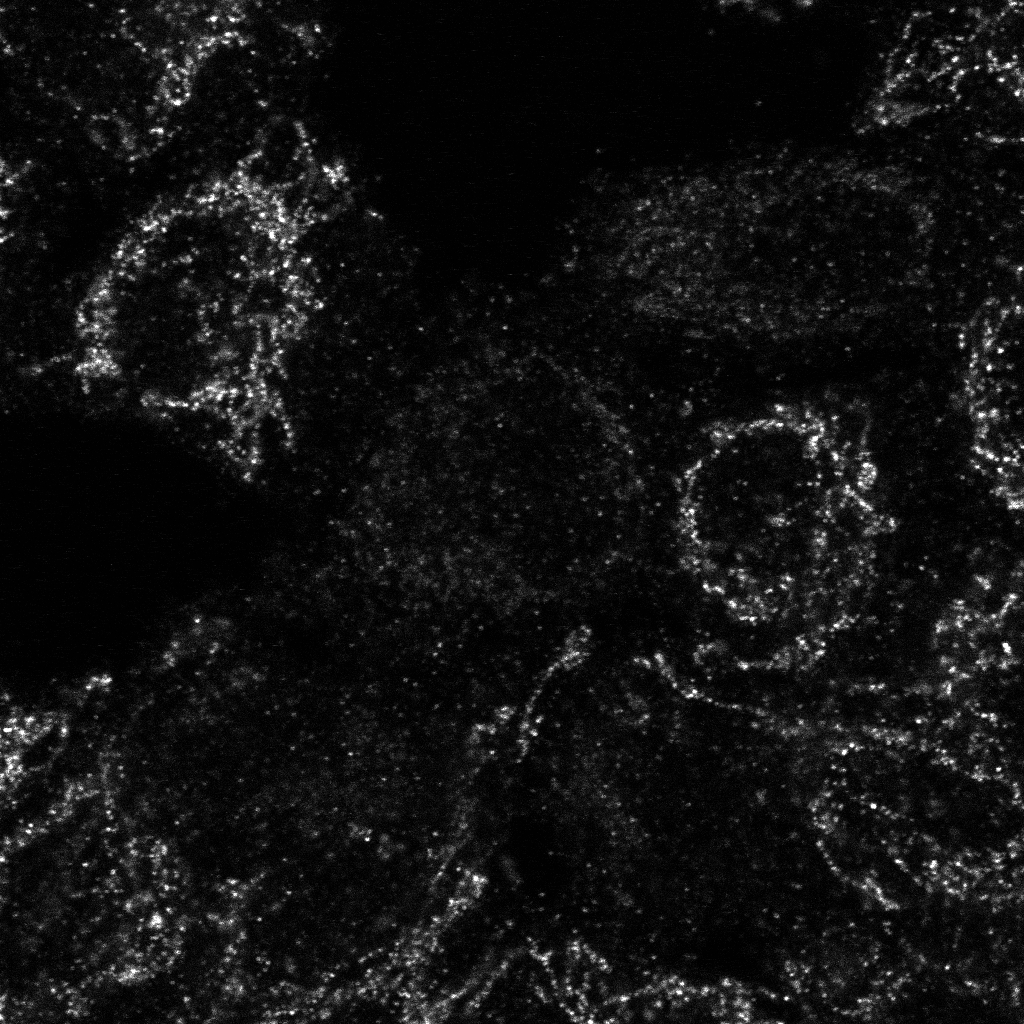

Supplement: Supplementary file 27 — Source data Fig. 5 [file 44318_2025_604_MOESM27_ESM.zip › Figure 5/5E/5E_image_TIMM23KOpool_channel3_antiTIMM23abAlexa594.tif]

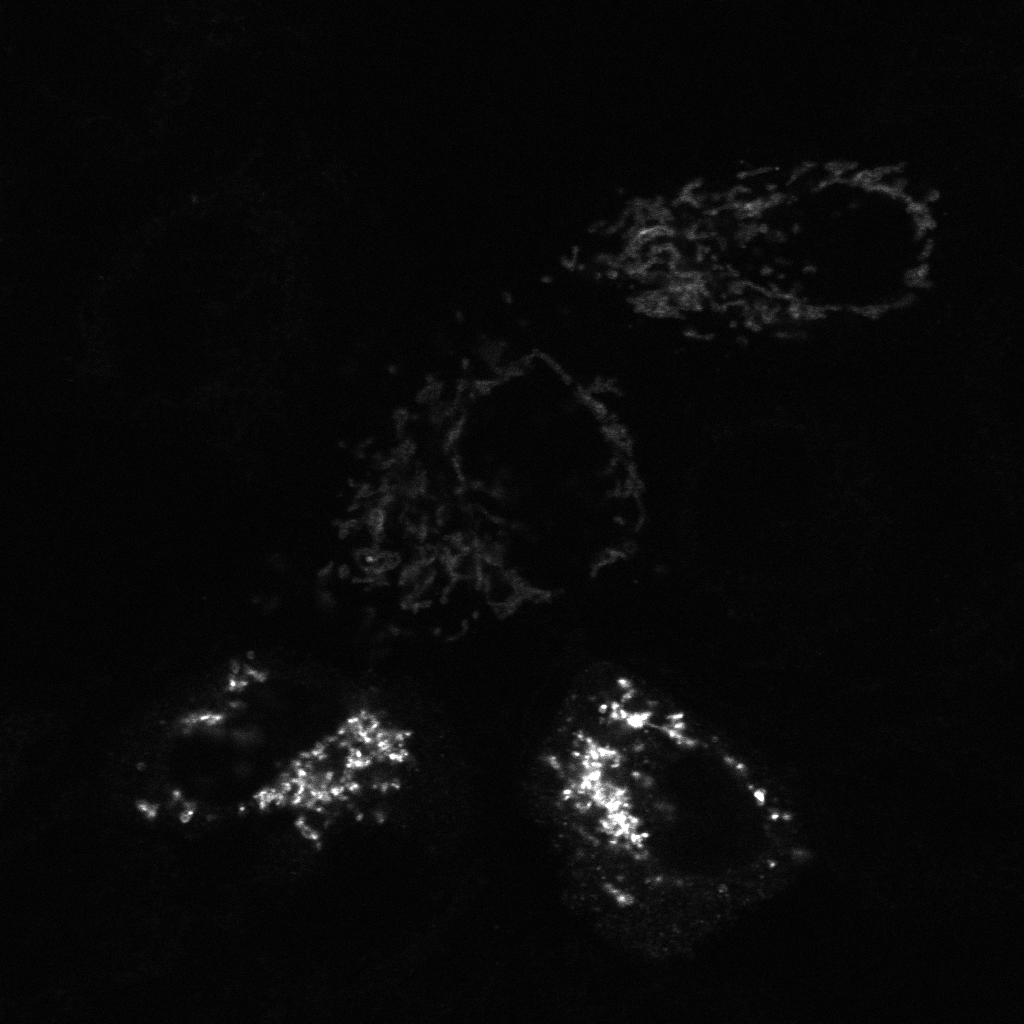

Supplement: Supplementary file 27 — Source data Fig. 5 [file 44318_2025_604_MOESM27_ESM.zip › Figure 5/5E/5E_image_TIMM23KOpool_channel2_PINK1YFP.tif]

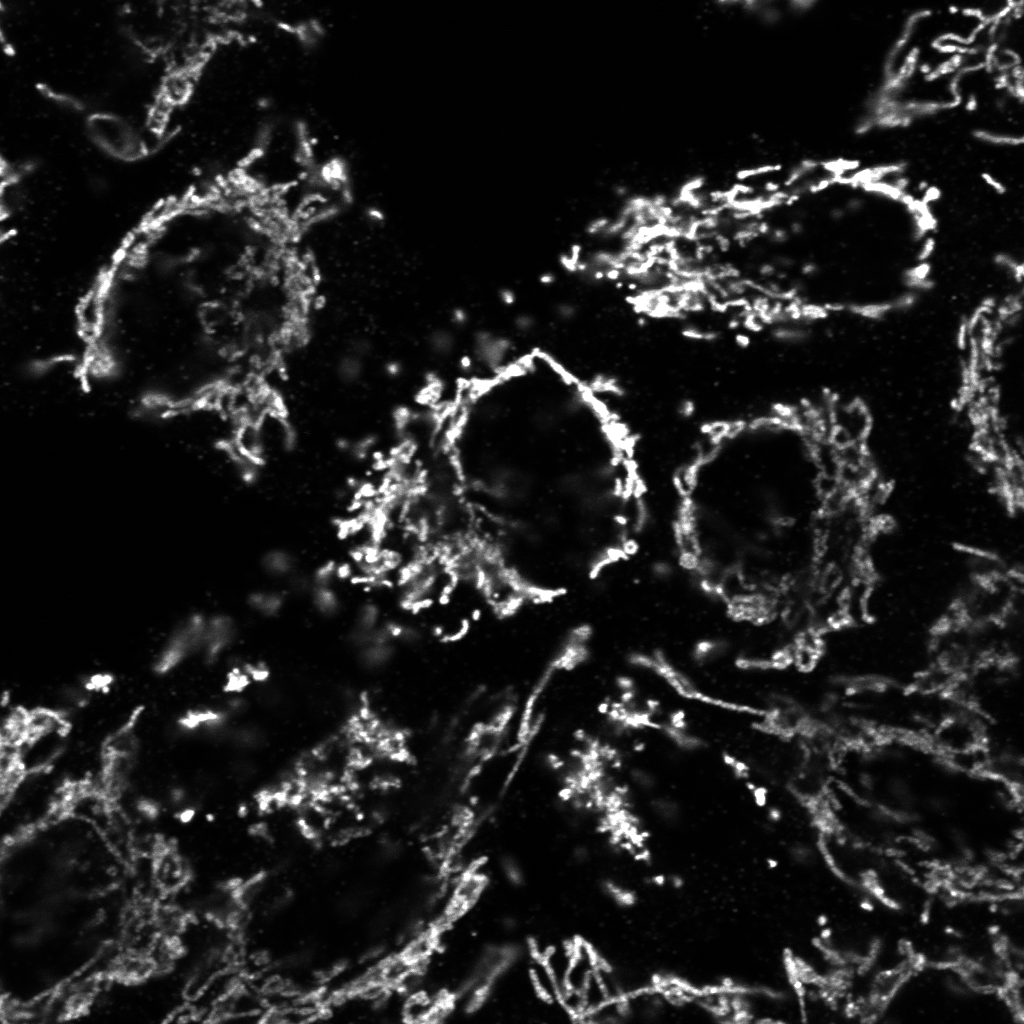

Supplement: Supplementary file 27 — Source data Fig. 5 [file 44318_2025_604_MOESM27_ESM.zip › Figure 5/5E/5E_image_TIMM23KOpool_channel4_antiTOMM40abAlexa647.tif]

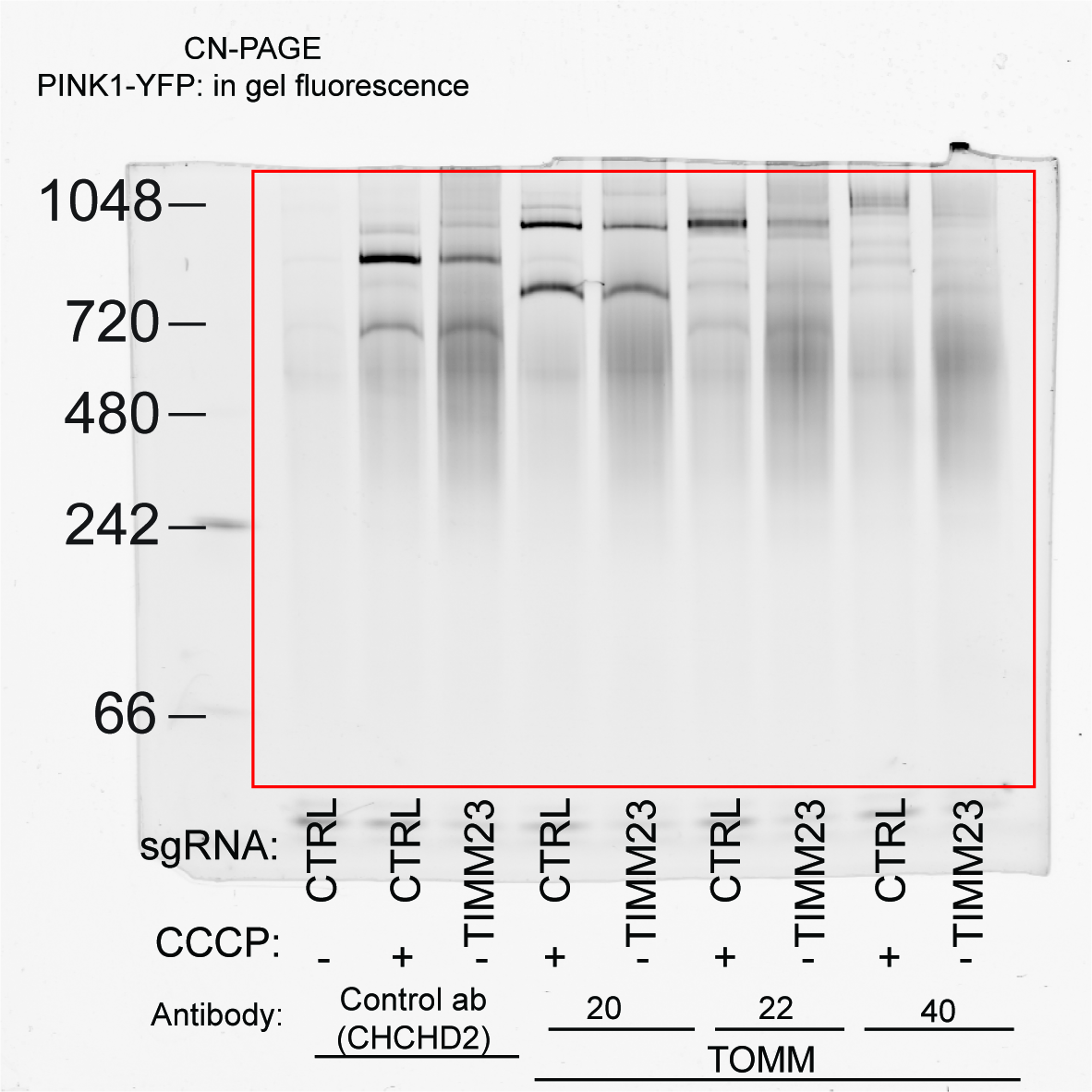

Supplement: Supplementary file 27 — Source data Fig. 5 [file 44318_2025_604_MOESM27_ESM.zip › Figure 5/5J/5J_CN_PAGE image.tif]

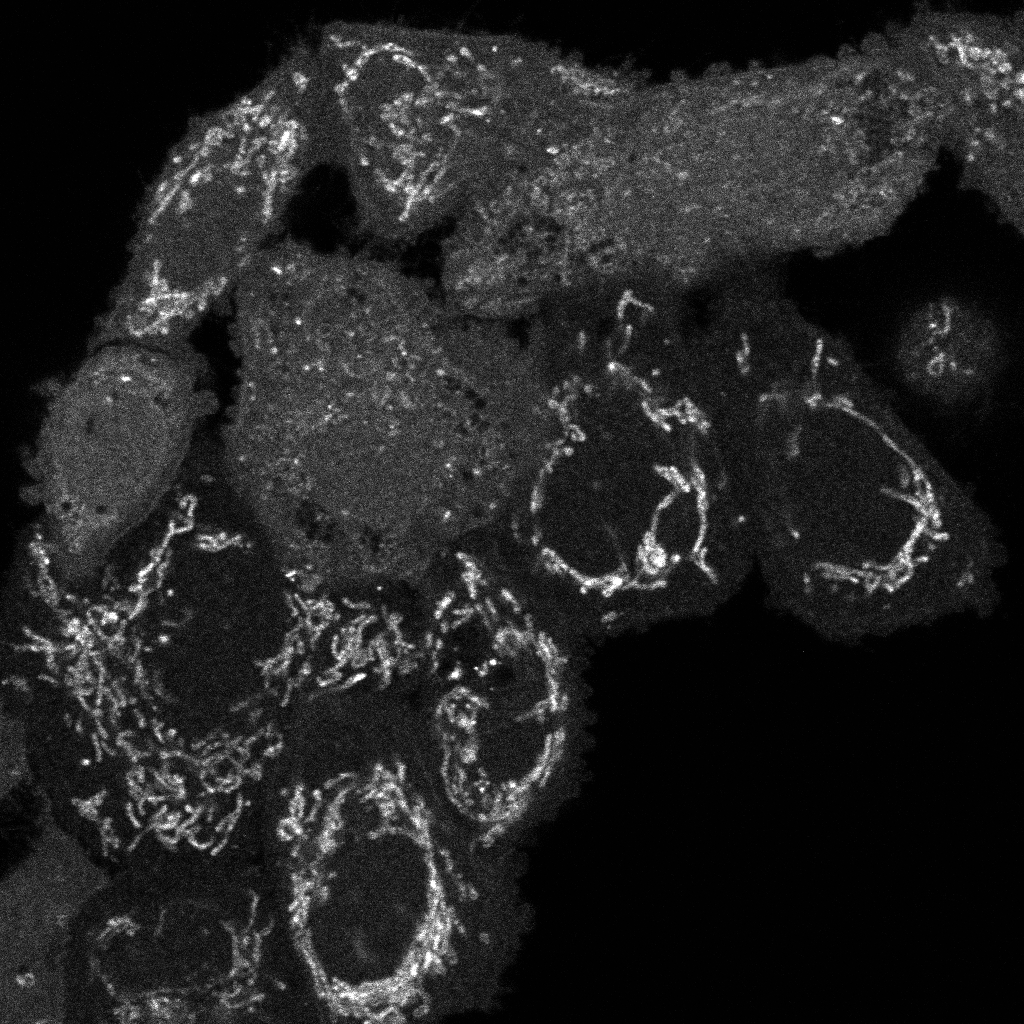

Supplement: Supplementary file 28 — Source data Fig. 6 - except I [file 44318_2025_604_MOESM28_ESM.zip › Figure 6 - except I/6C/6C_image_TIMM44KOpool_channel2_MTSmCherry.tif]

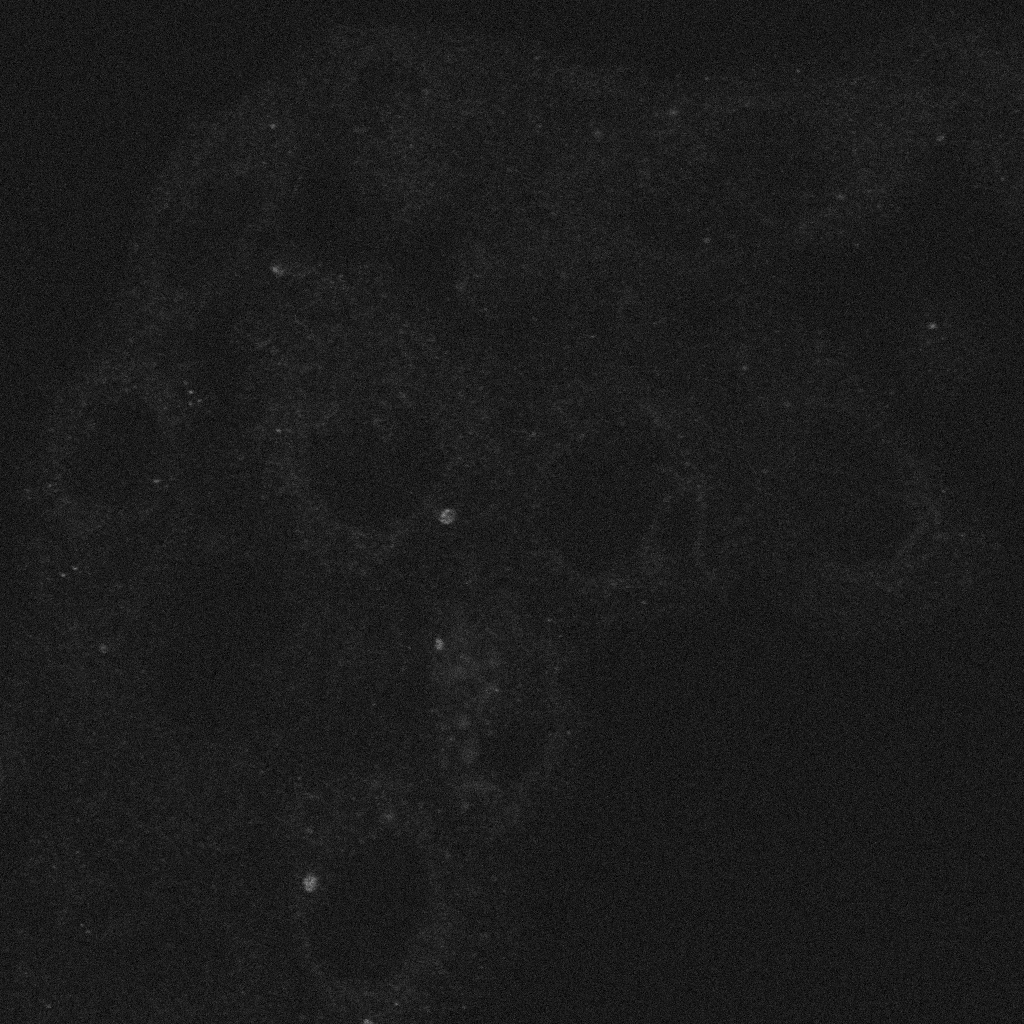

Supplement: Supplementary file 28 — Source data Fig. 6 - except I [file 44318_2025_604_MOESM28_ESM.zip › Figure 6 - except I/6C/6C_image_TIMM44KOpool_channel1_PINK1YFP.tif]

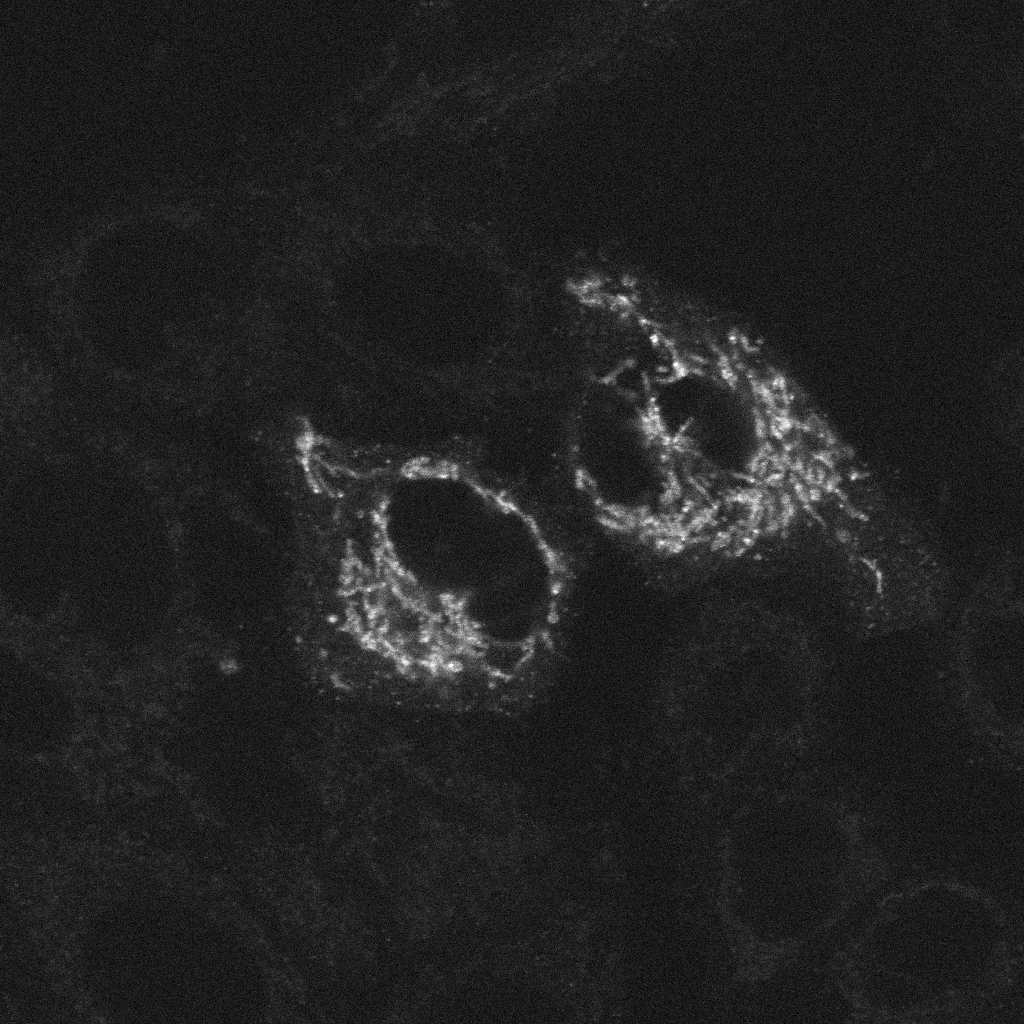

Supplement: Supplementary file 28 — Source data Fig. 6 - except I [file 44318_2025_604_MOESM28_ESM.zip › Figure 6 - except I/6C/6C_image_TIMM23KOpool_channel1_PINK1YFP.tif]

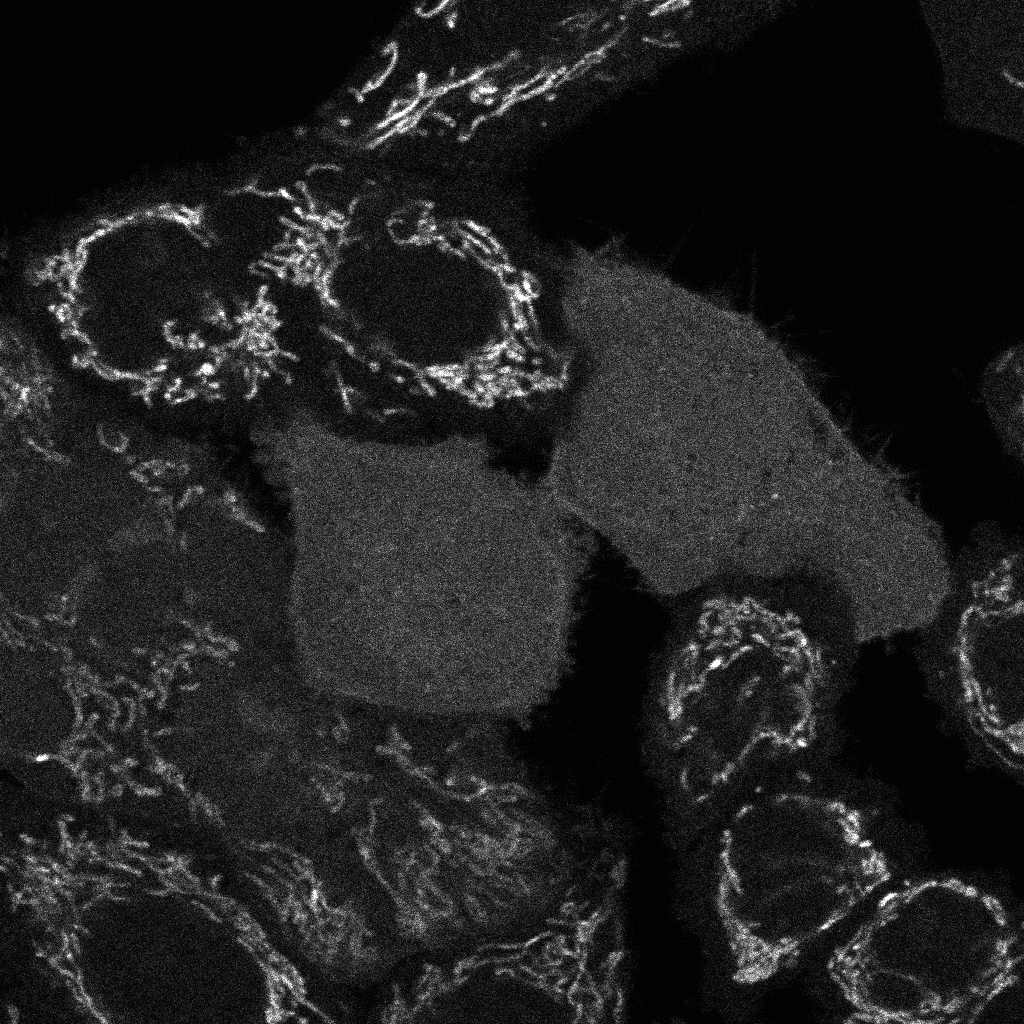

Supplement: Supplementary file 28 — Source data Fig. 6 - except I [file 44318_2025_604_MOESM28_ESM.zip › Figure 6 - except I/6C/6C_image_TIMM23KOpool_channel2_MTSmCherry.tif]

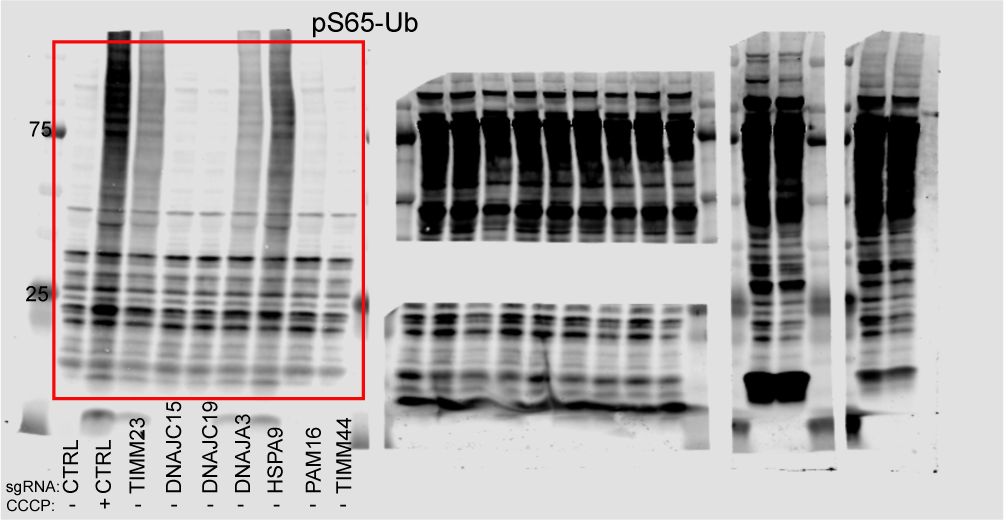

Supplement: Supplementary file 28 — Source data Fig. 6 - except I [file 44318_2025_604_MOESM28_ESM.zip › Figure 6 - except I/6A/6A_western pS65 Ub.tif]

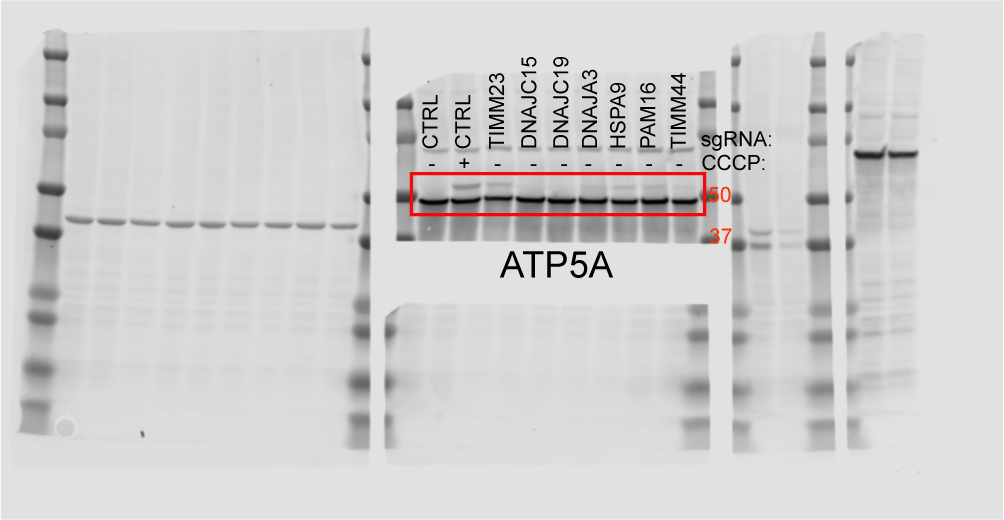

Supplement: Supplementary file 28 — Source data Fig. 6 - except I [file 44318_2025_604_MOESM28_ESM.zip › Figure 6 - except I/6A/6A_western atp5a.tif]

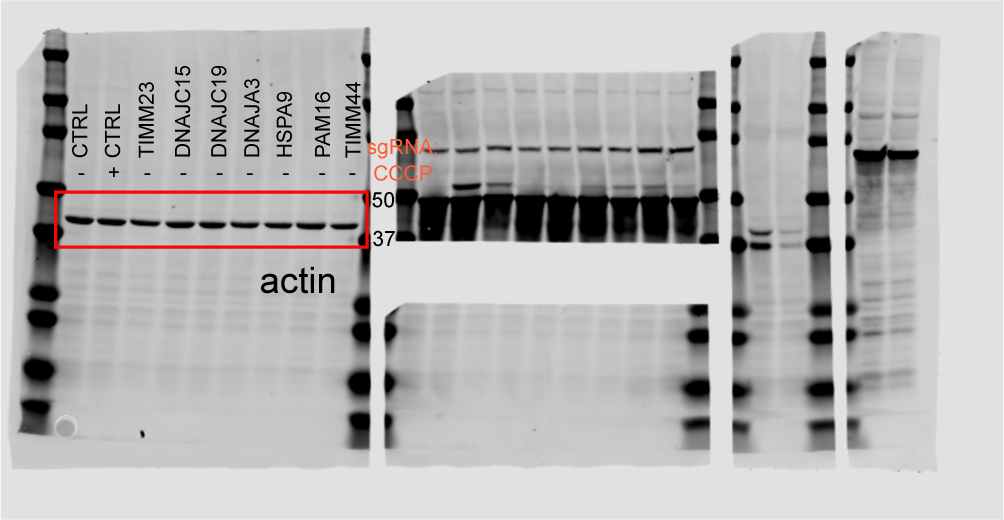

Supplement: Supplementary file 28 — Source data Fig. 6 - except I [file 44318_2025_604_MOESM28_ESM.zip › Figure 6 - except I/6A/6A_western actin for pS65 Ub.tif]

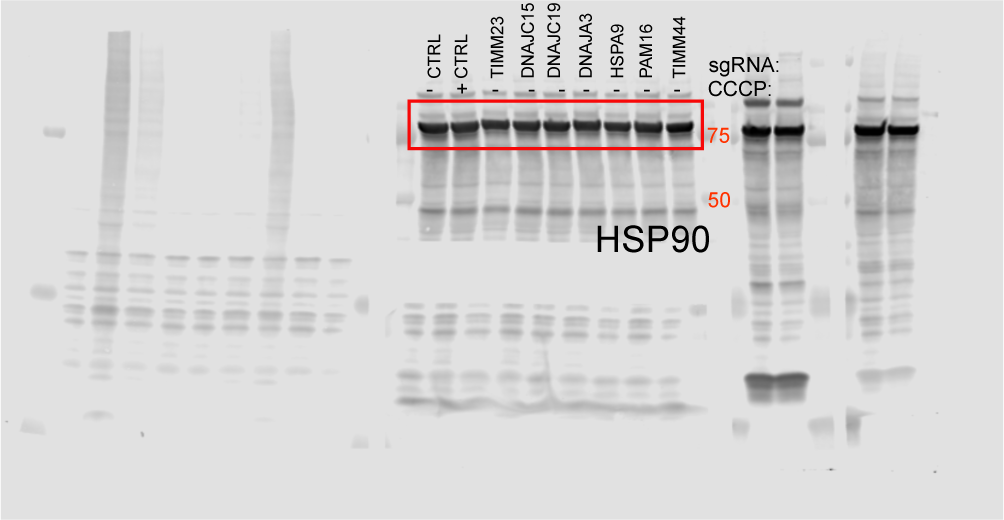

Supplement: Supplementary file 28 — Source data Fig. 6 - except I [file 44318_2025_604_MOESM28_ESM.zip › Figure 6 - except I/6A/6A_western hsp90 for atp5a.tif]

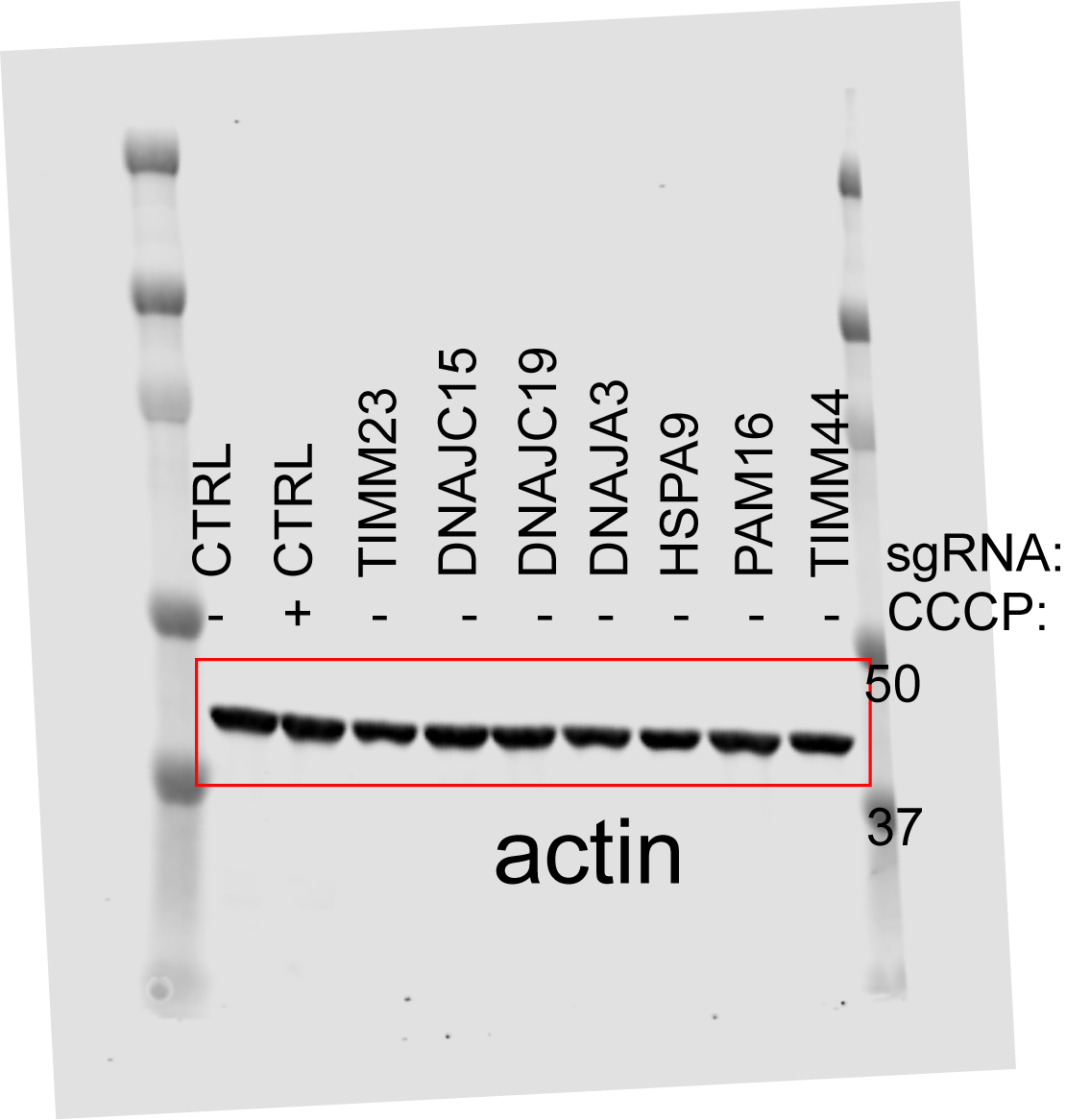

Supplement: Supplementary file 28 — Source data Fig. 6 - except I [file 44318_2025_604_MOESM28_ESM.zip › Figure 6 - except I/6A/6A_western actin for pink1_tilt_3degrees.tif]

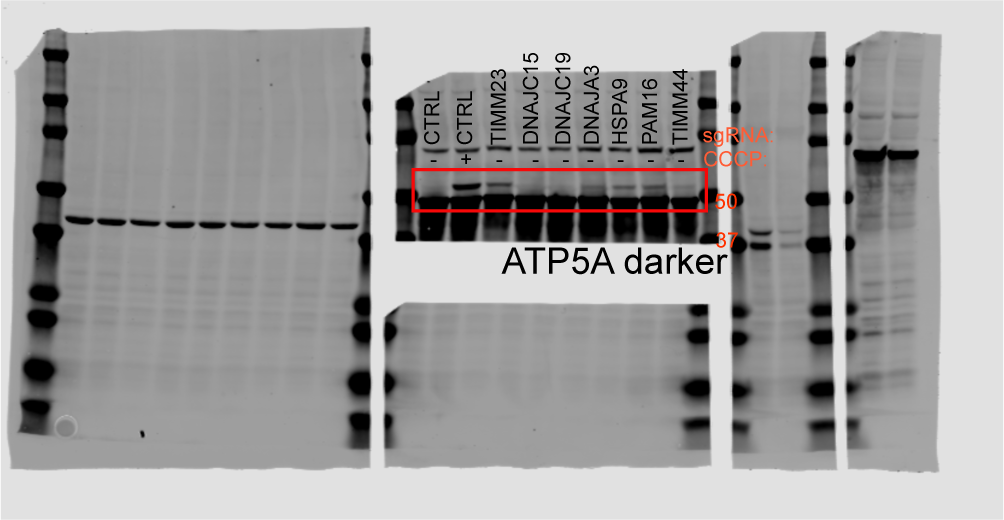

Supplement: Supplementary file 28 — Source data Fig. 6 - except I [file 44318_2025_604_MOESM28_ESM.zip › Figure 6 - except I/6A/6A_western atp5a darker.tif]

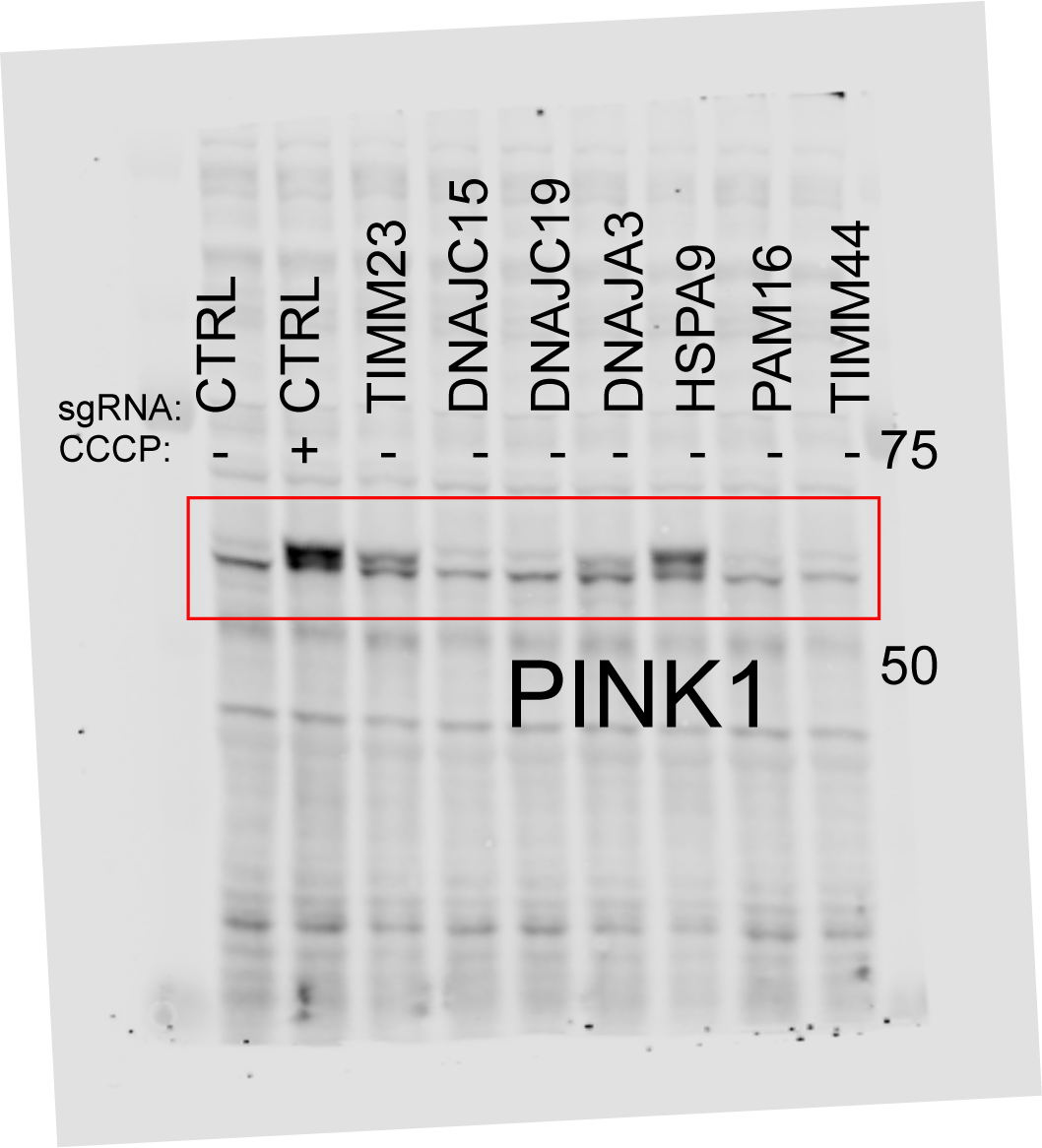

Supplement: Supplementary file 28 — Source data Fig. 6 - except I [file 44318_2025_604_MOESM28_ESM.zip › Figure 6 - except I/6A/6A_western pink1_tilt_3degrees.tif]

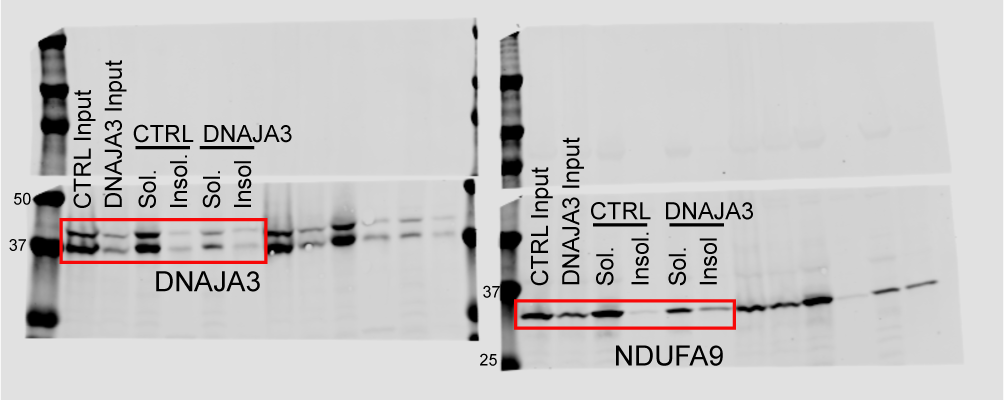

Supplement: Supplementary file 28 — Source data Fig. 6 - except I [file 44318_2025_604_MOESM28_ESM.zip › Figure 6 - except I/6E/6E_western dnaja3 and ndufa9.tif]

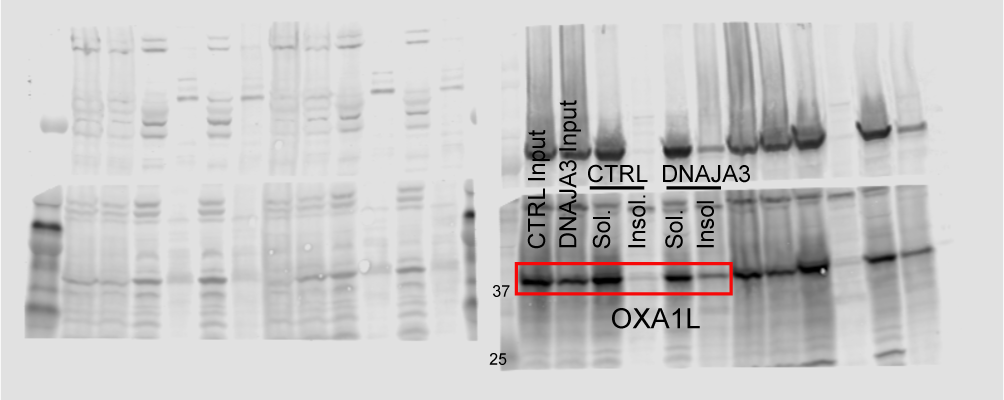

Supplement: Supplementary file 28 — Source data Fig. 6 - except I [file 44318_2025_604_MOESM28_ESM.zip › Figure 6 - except I/6E/6E_western oxa1L.tif]

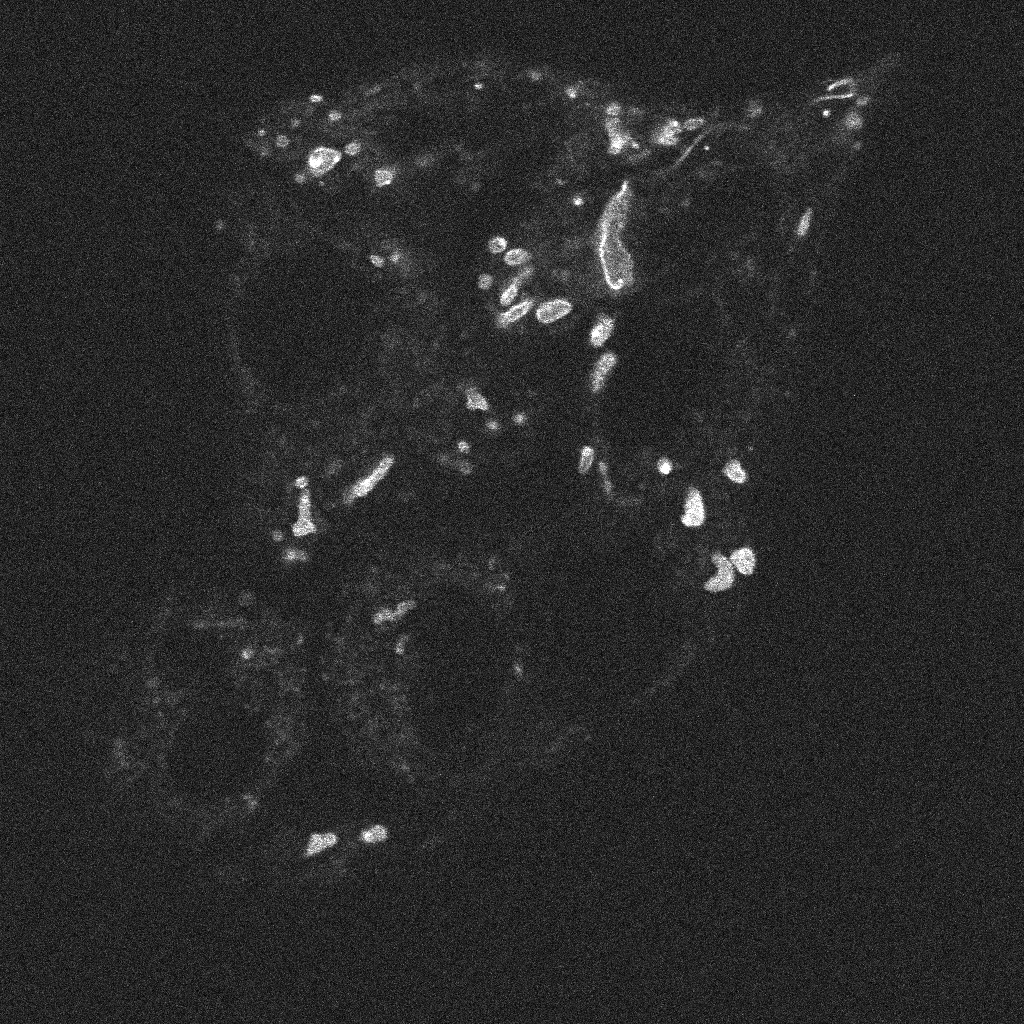

Supplement: Supplementary file 29 — Source data Fig. 6I Part 1 [file 44318_2025_604_MOESM29_ESM.zip › Fig6I_confocal and EM mitochondria/6I_channel 1_PINK1 YFP.tif]

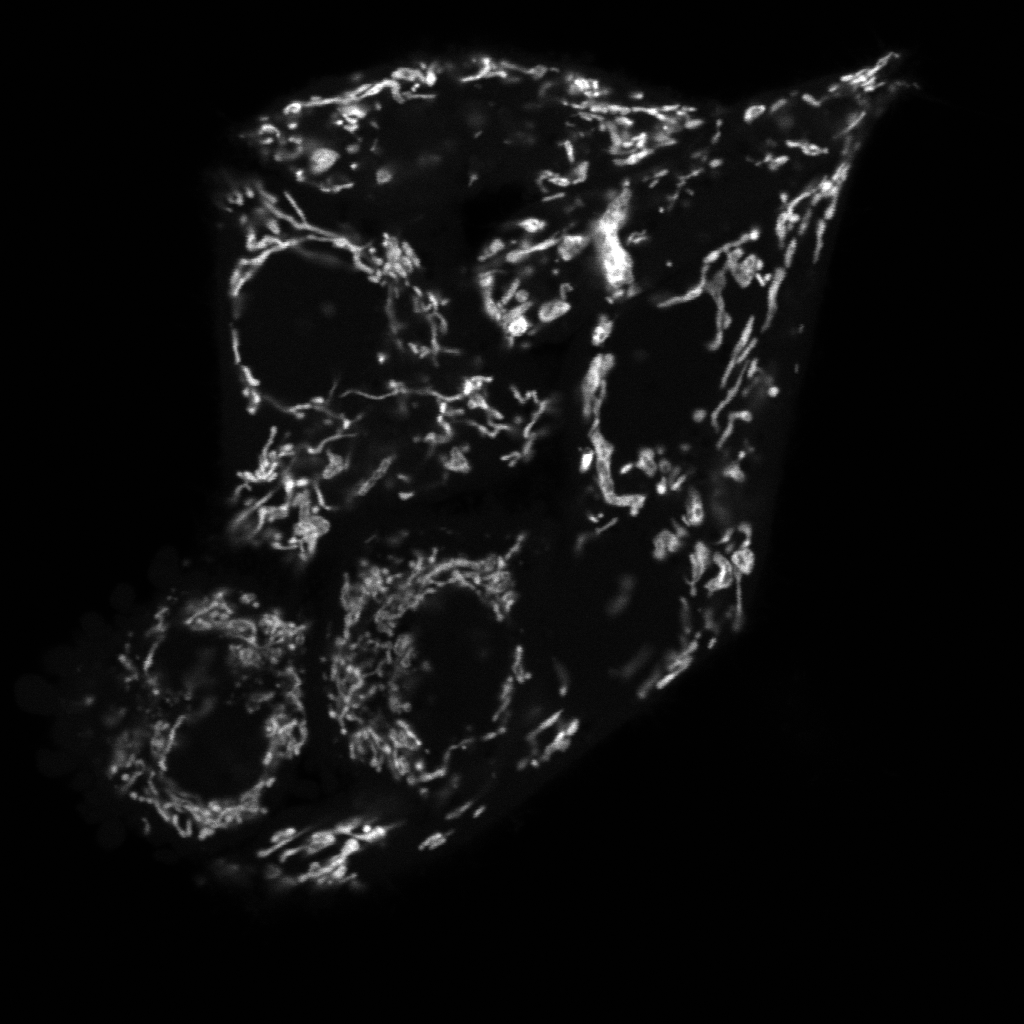

Supplement: Supplementary file 29 — Source data Fig. 6I Part 1 [file 44318_2025_604_MOESM29_ESM.zip › Fig6I_confocal and EM mitochondria/6I_channel 2_mts mCherry.tif]

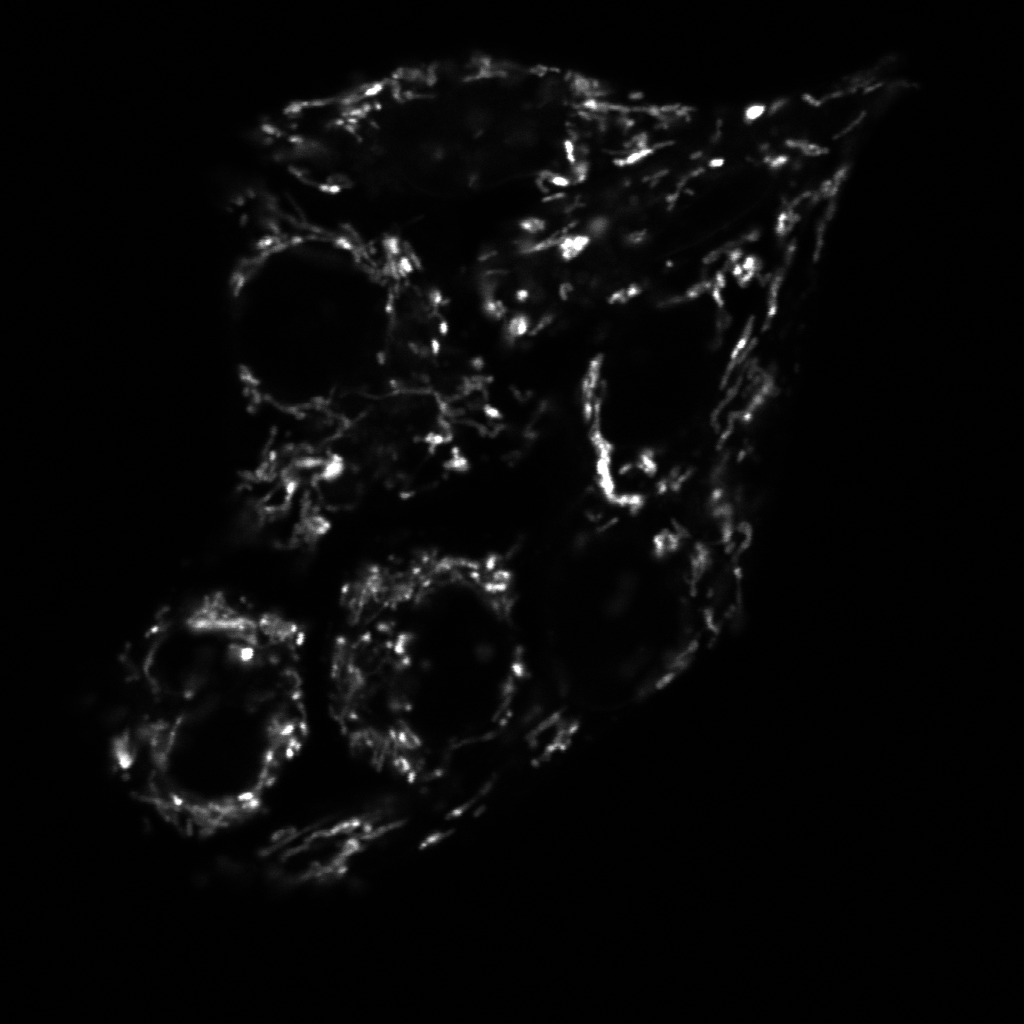

Supplement: Supplementary file 29 — Source data Fig. 6I Part 1 [file 44318_2025_604_MOESM29_ESM.zip › Fig6I_confocal and EM mitochondria/6I_channel 3_MMP.tif]

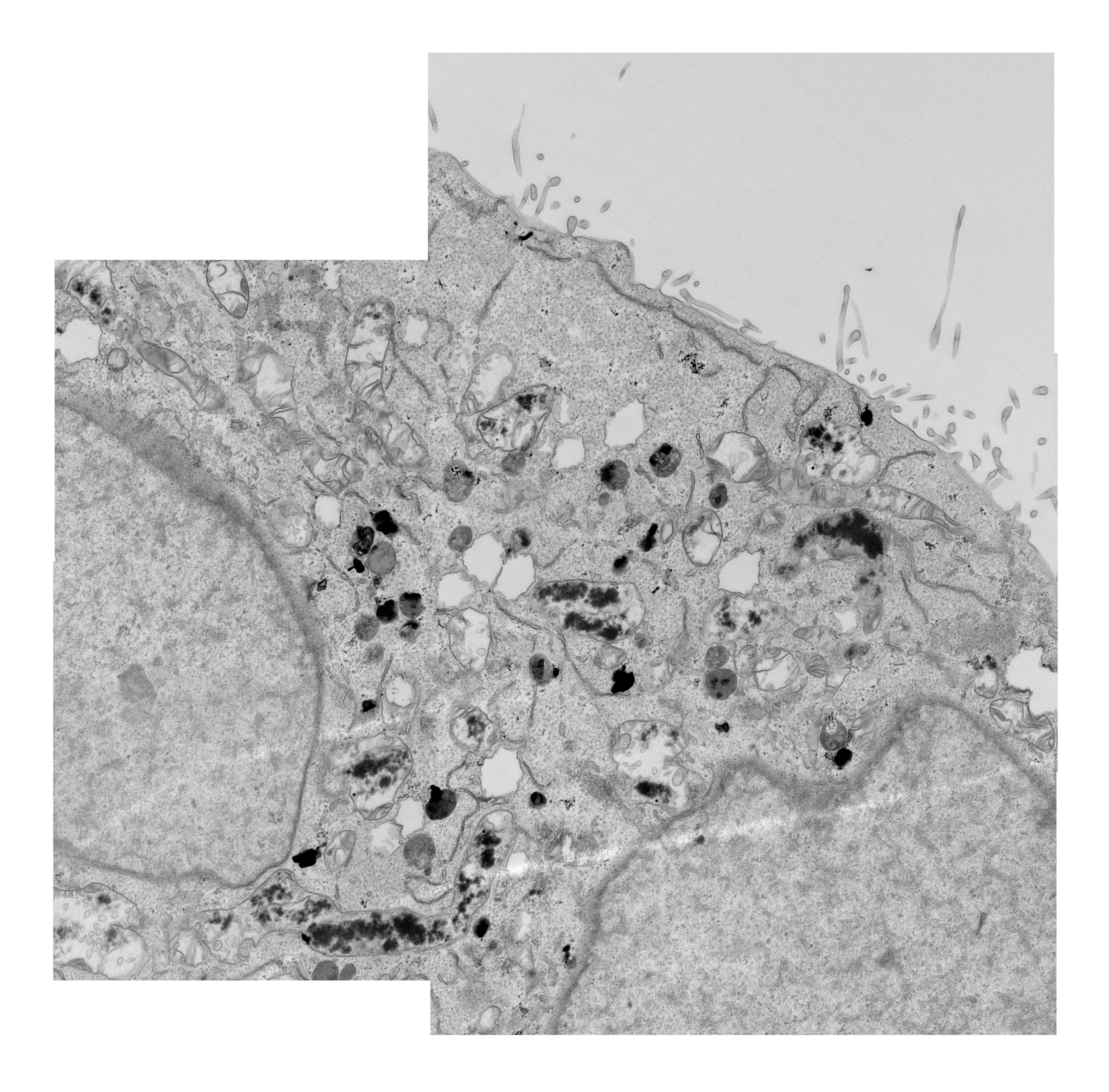

Supplement: Supplementary file 30 — Source data Fig. 6I Part 2 [file 44318_2025_604_MOESM30_ESM.zip › Fig6I_EM_field of view for montage/6I_montage field of view_Fig6I_300dpi.tif]

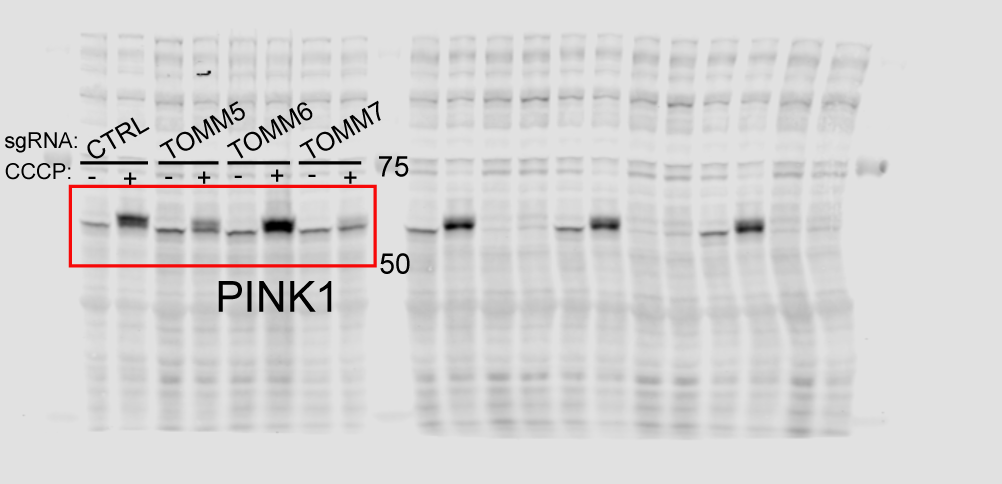

Supplement: Supplementary file 31 — Source data Fig. 7 [file 44318_2025_604_MOESM31_ESM.zip › Figure 7/7B/7B right blot_western pink1.tif]

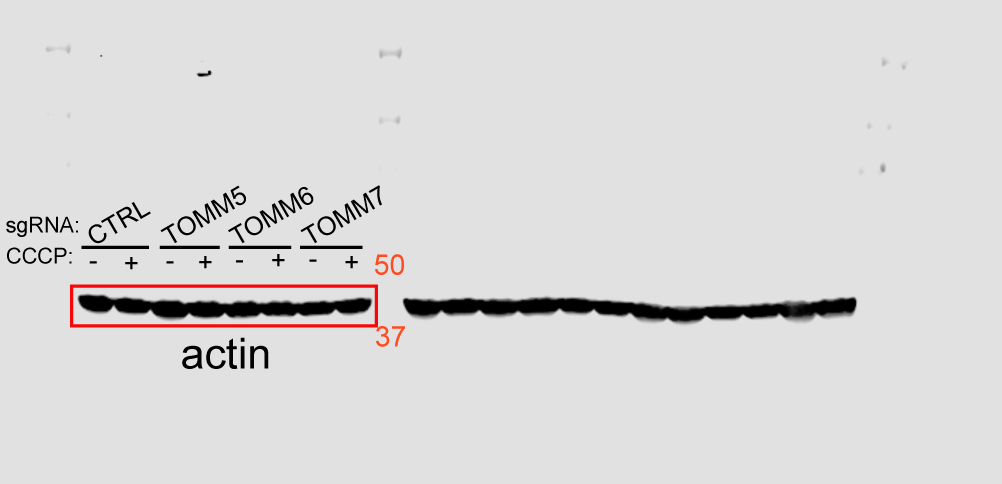

Supplement: Supplementary file 31 — Source data Fig. 7 [file 44318_2025_604_MOESM31_ESM.zip › Figure 7/7B/7B right blot_western actin for pink1.tif]

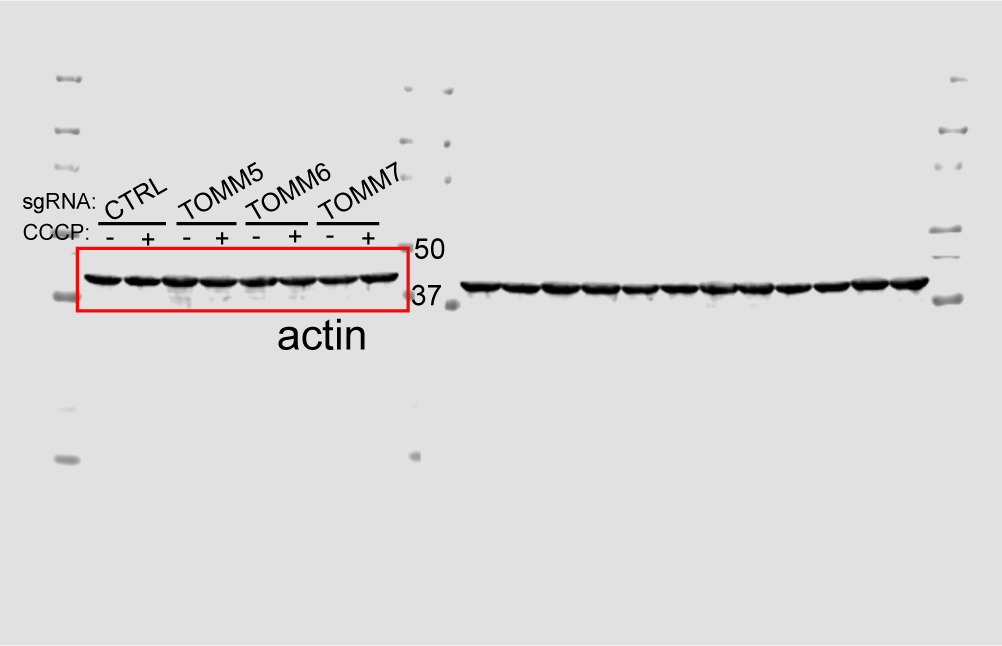

Supplement: Supplementary file 31 — Source data Fig. 7 [file 44318_2025_604_MOESM31_ESM.zip › Figure 7/7B/7B right blot_western actin for tomm7.tif]

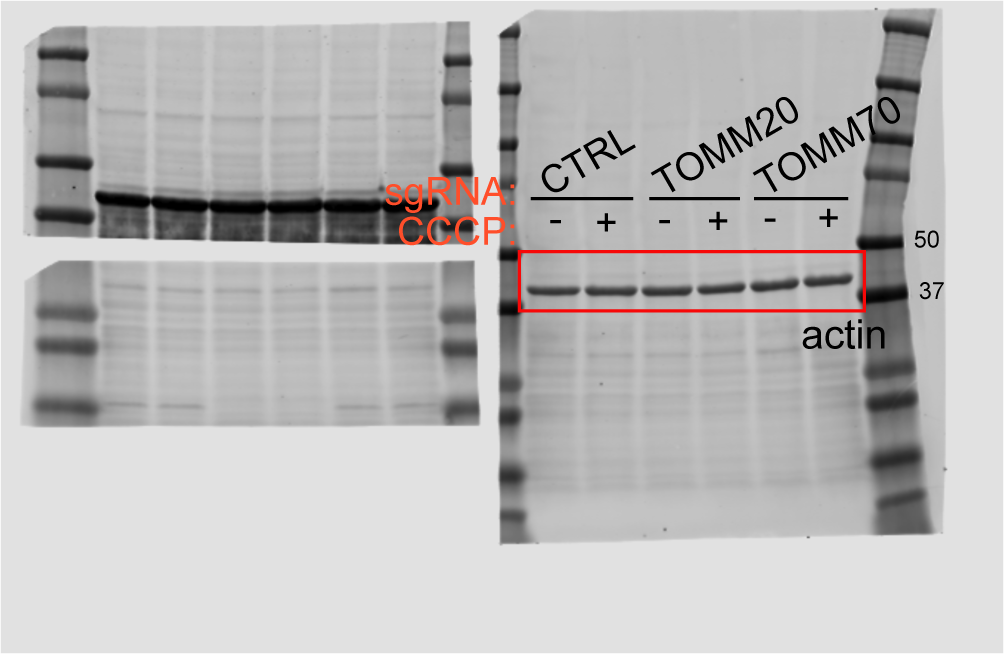

Supplement: Supplementary file 31 — Source data Fig. 7 [file 44318_2025_604_MOESM31_ESM.zip › Figure 7/7B/7B left blot_western actin for pS65 Ub.tif]

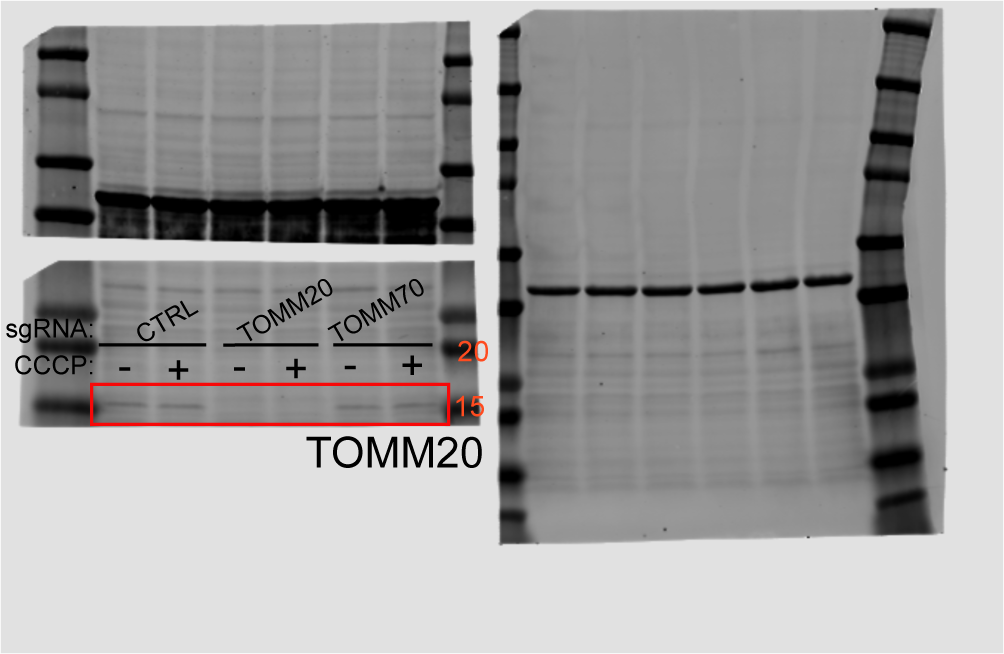

Supplement: Supplementary file 31 — Source data Fig. 7 [file 44318_2025_604_MOESM31_ESM.zip › Figure 7/7B/7B left blot_western tom20.tif]

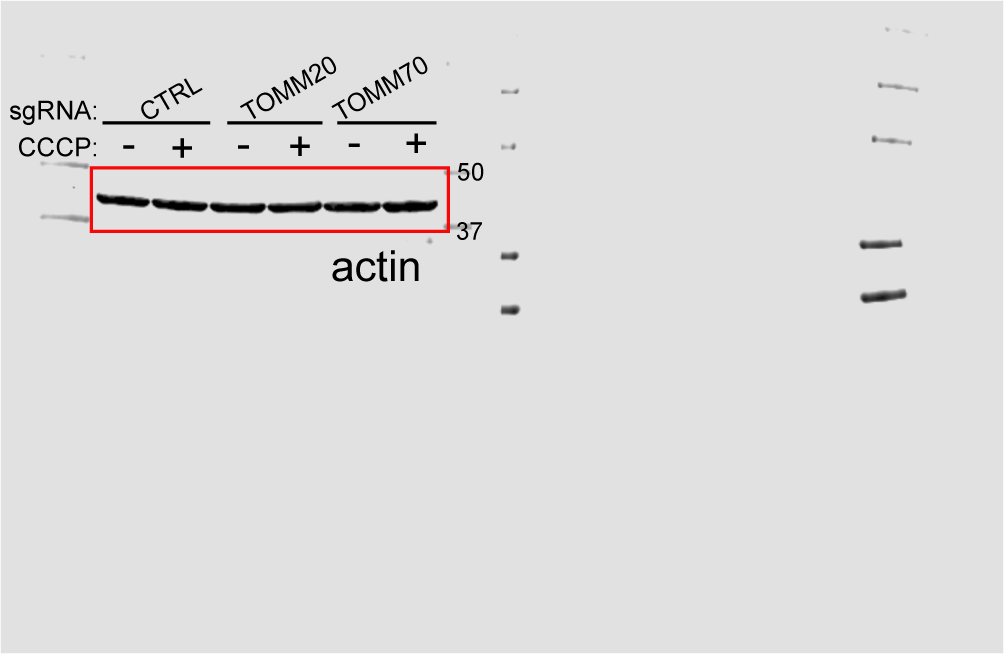

Supplement: Supplementary file 31 — Source data Fig. 7 [file 44318_2025_604_MOESM31_ESM.zip › Figure 7/7B/7B left blot_western actin for tomm70 and tomm20.tif]

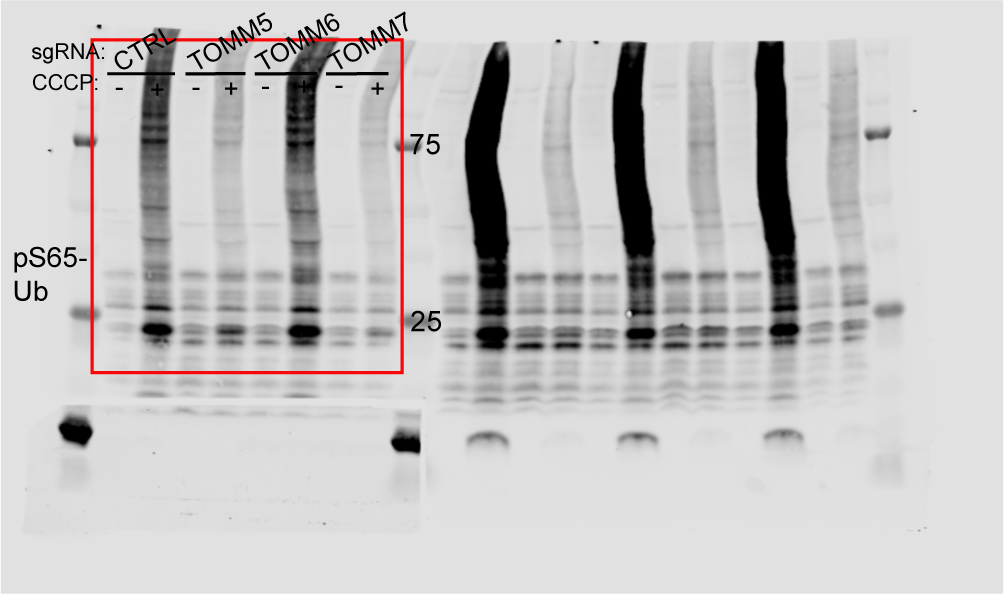

Supplement: Supplementary file 31 — Source data Fig. 7 [file 44318_2025_604_MOESM31_ESM.zip › Figure 7/7B/7B right blot_western pS65 Ub.tif]
