# Supplementary figures and images for: A unified mechanism for mitochondrial damage sensing in PINK1-Parkin–mediated mitophagy (part 2 of 2)
Source: EMBO J. 2025 Nov 20;45(1):64–105. doi: 10.1038/s44318-025-00604-z (PMC12759083; doi:10.1038/s44318-025-00604-z)

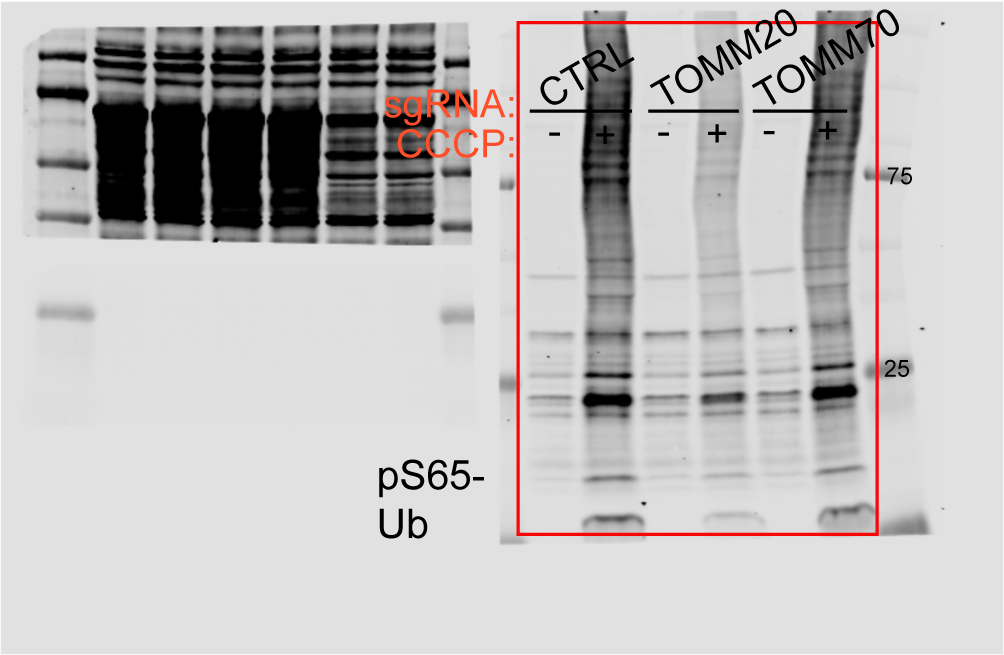

Supplement: Supplementary file 31 — Source data Fig. 7 [file 44318_2025_604_MOESM31_ESM.zip › Figure 7/7B/7B left blot_western pS65 Ub.tif]

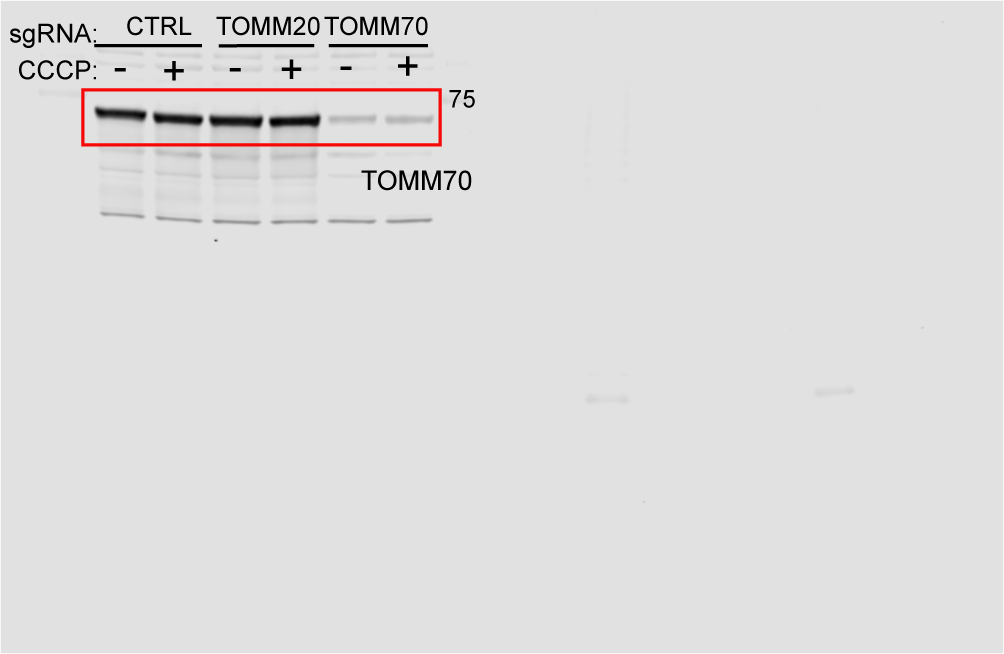

Supplement: Supplementary file 31 — Source data Fig. 7 [file 44318_2025_604_MOESM31_ESM.zip › Figure 7/7B/7B left blot_western tomm70.tif]

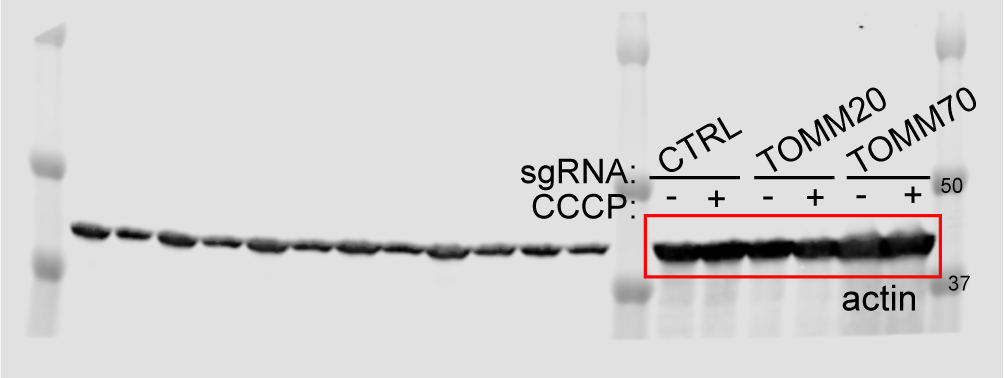

Supplement: Supplementary file 31 — Source data Fig. 7 [file 44318_2025_604_MOESM31_ESM.zip › Figure 7/7B/7B left blot_western actin for pink1.tif]

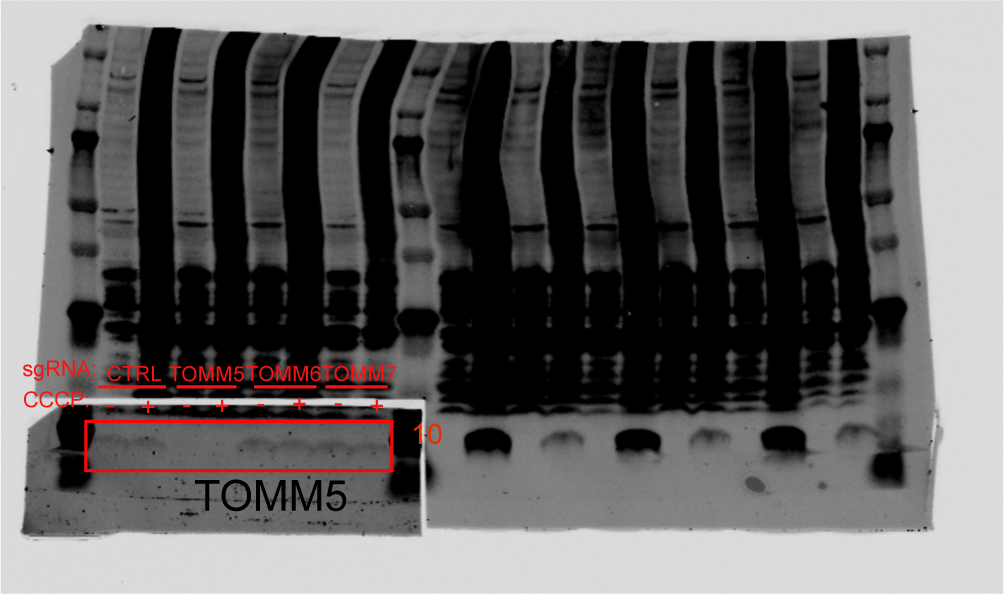

Supplement: Supplementary file 31 — Source data Fig. 7 [file 44318_2025_604_MOESM31_ESM.zip › Figure 7/7B/7B right blot_western tomm5.tif]

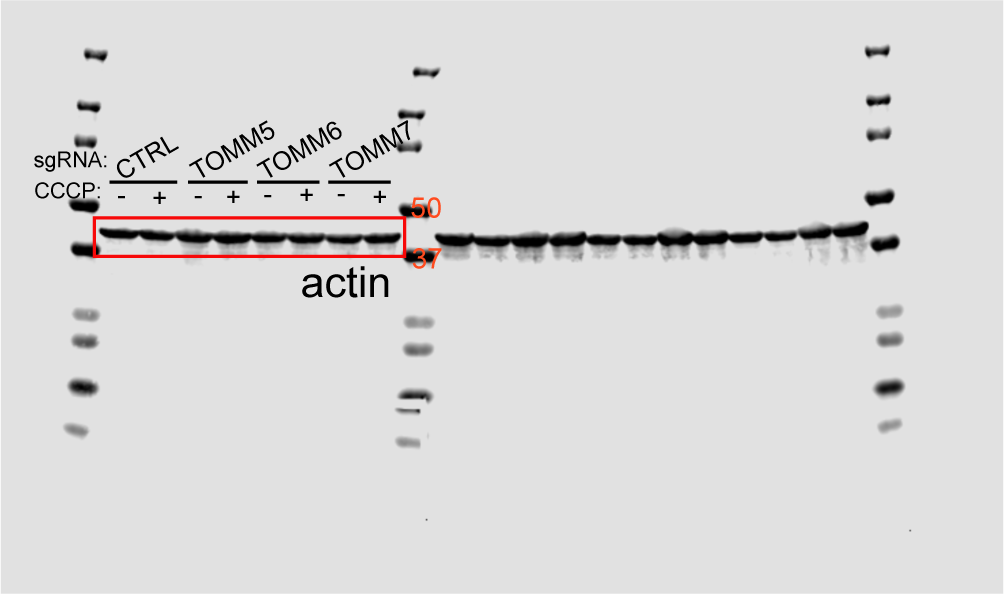

Supplement: Supplementary file 31 — Source data Fig. 7 [file 44318_2025_604_MOESM31_ESM.zip › Figure 7/7B/7B right blot_western actin for pS65 Ub and tomm5.tif]

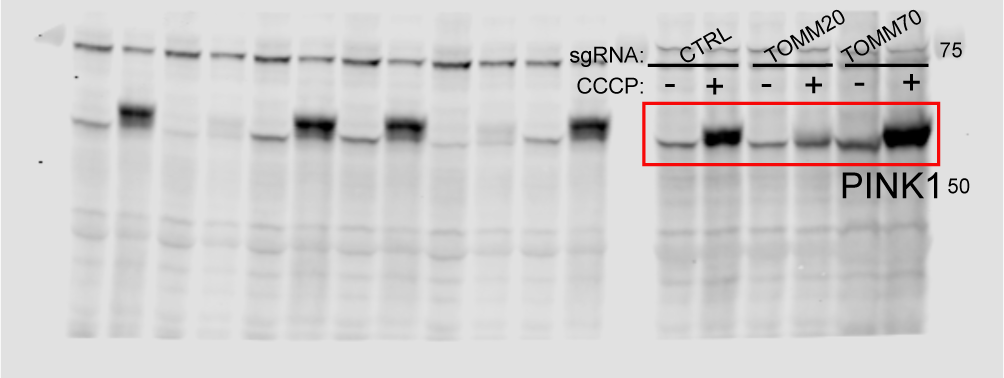

Supplement: Supplementary file 31 — Source data Fig. 7 [file 44318_2025_604_MOESM31_ESM.zip › Figure 7/7B/7B left blot_western pink1.tif]

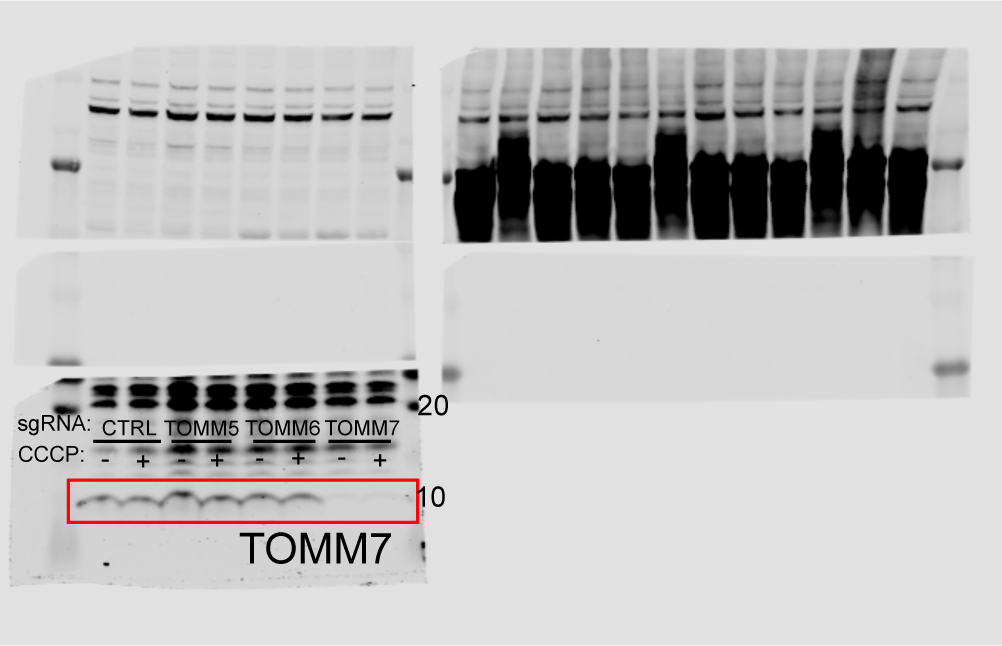

Supplement: Supplementary file 31 — Source data Fig. 7 [file 44318_2025_604_MOESM31_ESM.zip › Figure 7/7B/7B right blot_western tomm7.tif]

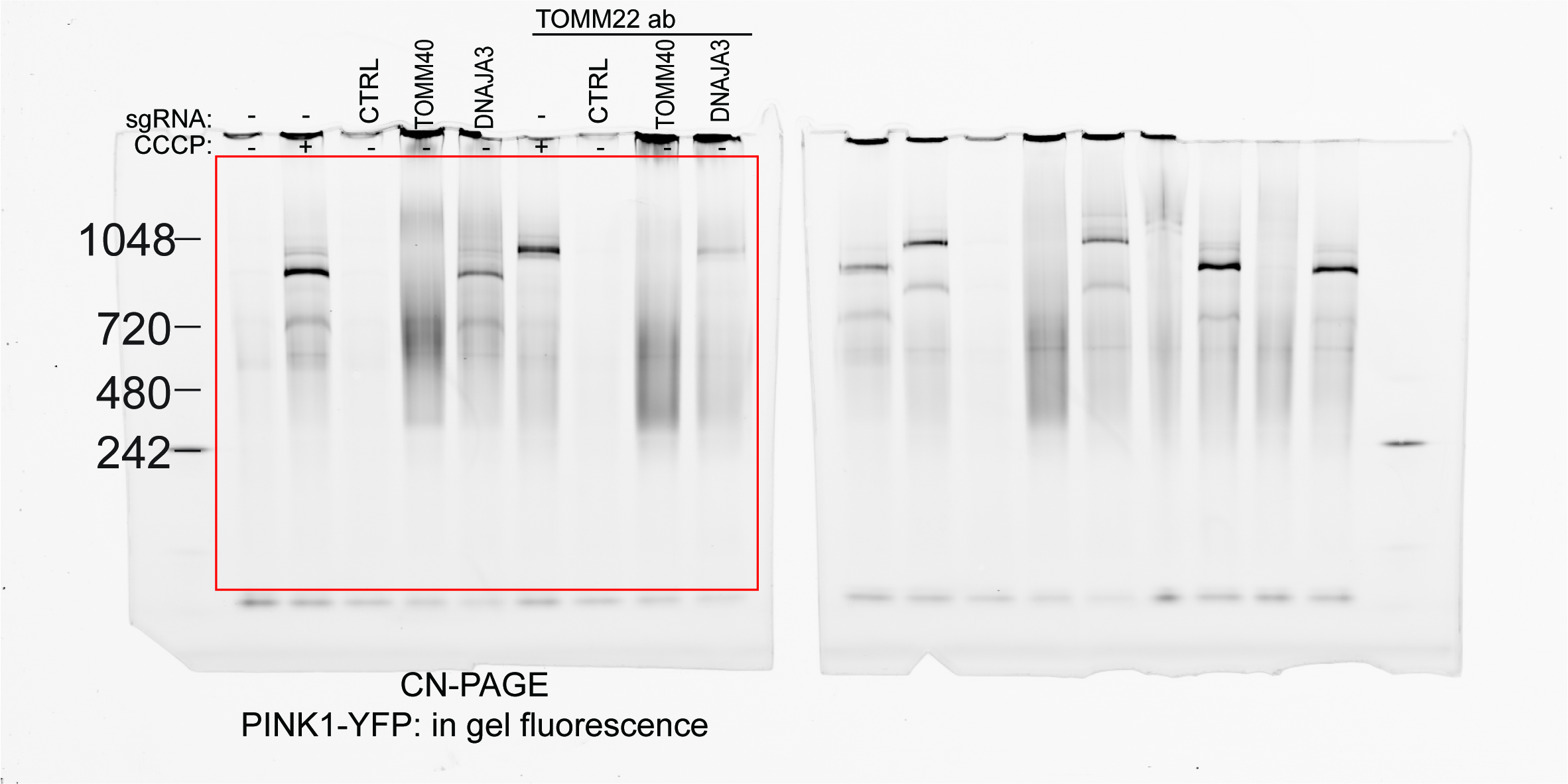

Supplement: Supplementary file 31 — Source data Fig. 7 [file 44318_2025_604_MOESM31_ESM.zip › Figure 7/7D/7D_CN_PAGE tom40 sgrna_image.tif]
