# Supplementary material for: A unified mechanism for mitochondrial damage sensing in PINK1-Parkin–mediated mitophagy
Source: EMBO J. 2025 Nov 20;45(1):64–105. doi: 10.1038/s44318-025-00604-z (PMC12759083; doi:10.1038/s44318-025-00604-z)
Supplement: Supplementary file 32 — Expanded View Figures [file 44318_2025_604_MOESM32_ESM.pdf]

Expanded View Figures

Figure EV1. APEX-ER staining reliably identifies HeLa cells with knockdown of mitochondrial proteins for assessment of mitochondrial ultrastructure by TEM.

(**A**) Transmitted light image of HeLa$^{dCas9\text{-}BFP\text{-}ZIM3}$ cells shows the brown DAB reaction product after ~12 min of development time in cells transduced with APEX-ER (solid arrowheads) compared to cells expressing low or no APEX-ER (open arrowheads). Scale bar = 100 μm. (**B**) TEM image of a cell with low or no APEX-ER staining in the ER lumen (open arrowheads) next to a cell with darkly stained ER lumen (closed arrowheads) indicating a high level of APEX-ER expression. Scale bar = 2 μm. (**C**) Examples of abnormal ultrastructural features of mitochondria expressing sgRNAs for the protein indicated in the lower left corner of each image. Yellow arrows indicate the abnormal cristae feature listed at the top of each image; yellow asterisks indicate areas with sparse cristae. Red arrows indicate the fluffy aggregate observed in the matrix of cells transduced with sgRNA DNAJA3. Blue asterisks indicate cytosol enclosed cup-shaped mitochondria. In some cases, examples shown were cropped from the same cell. Scale bar = 500 nm.

 © The Author(s)

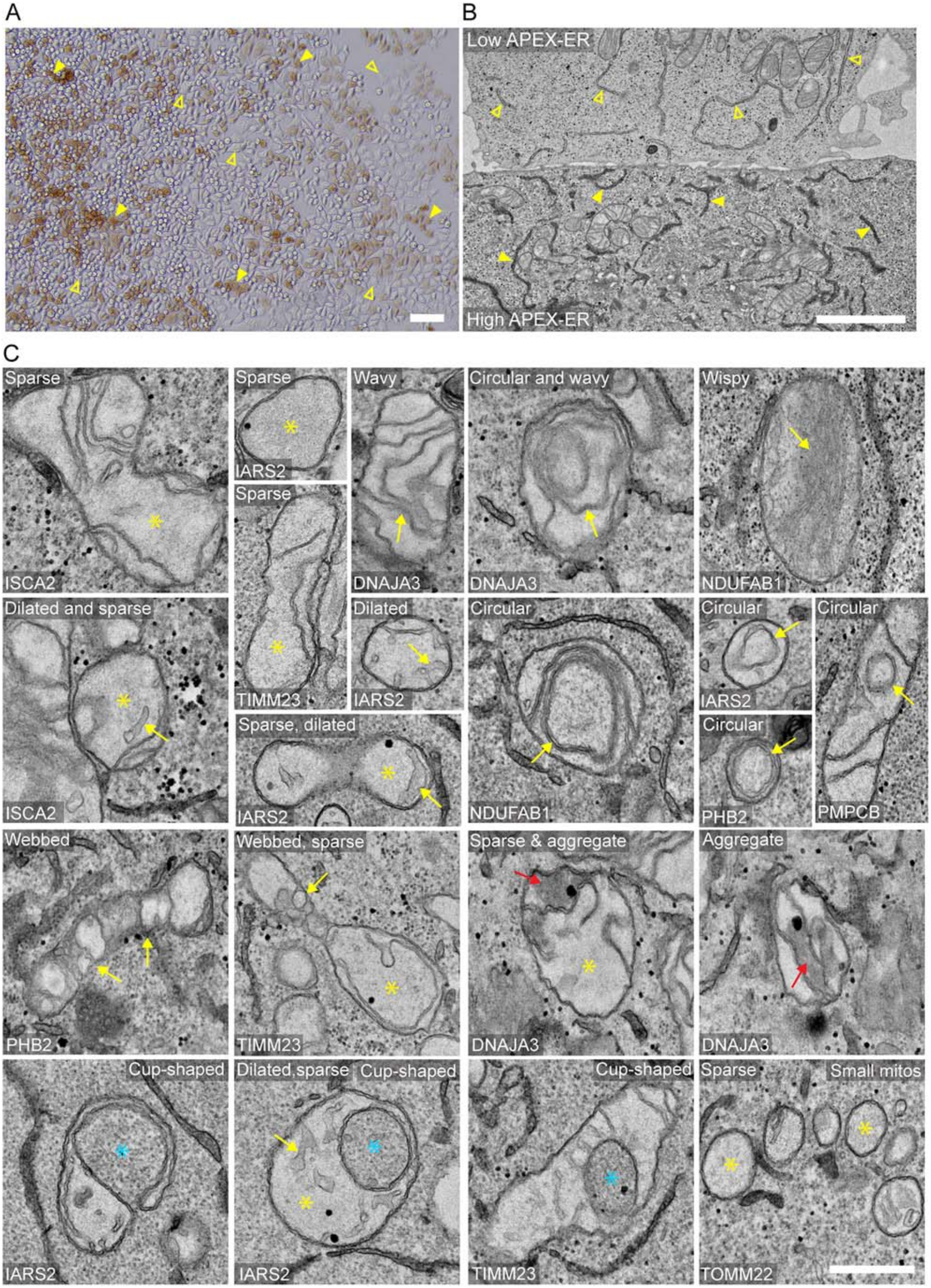
A
B
Low APEX-ER
High APEX-ER
C
Sparse
ISCA2
Sparse
IARS2
Sparse
TIMM23
Wavy
DNAJA3
Circular and wavy
DNAJA3
Wispy
NDUFAB1
Dilated and sparse
ISCA2
Dilated
IARS2
Sparse, dilated
IARS2
Circular
NDUFAB1
Circular
IARS2
Circular
PHB2
Circular
PMPCB
Webbed
PHB2
Webbed, sparse
TIMM23
Sparse & aggregate
DNAJA3
Aggregate
DNAJA3
Cup-shaped
IARS2
Dilated,sparse
Cup-shaped
IARS2
Cup-shaped
TIMM23
Sparse
Small mitos
TOMM22

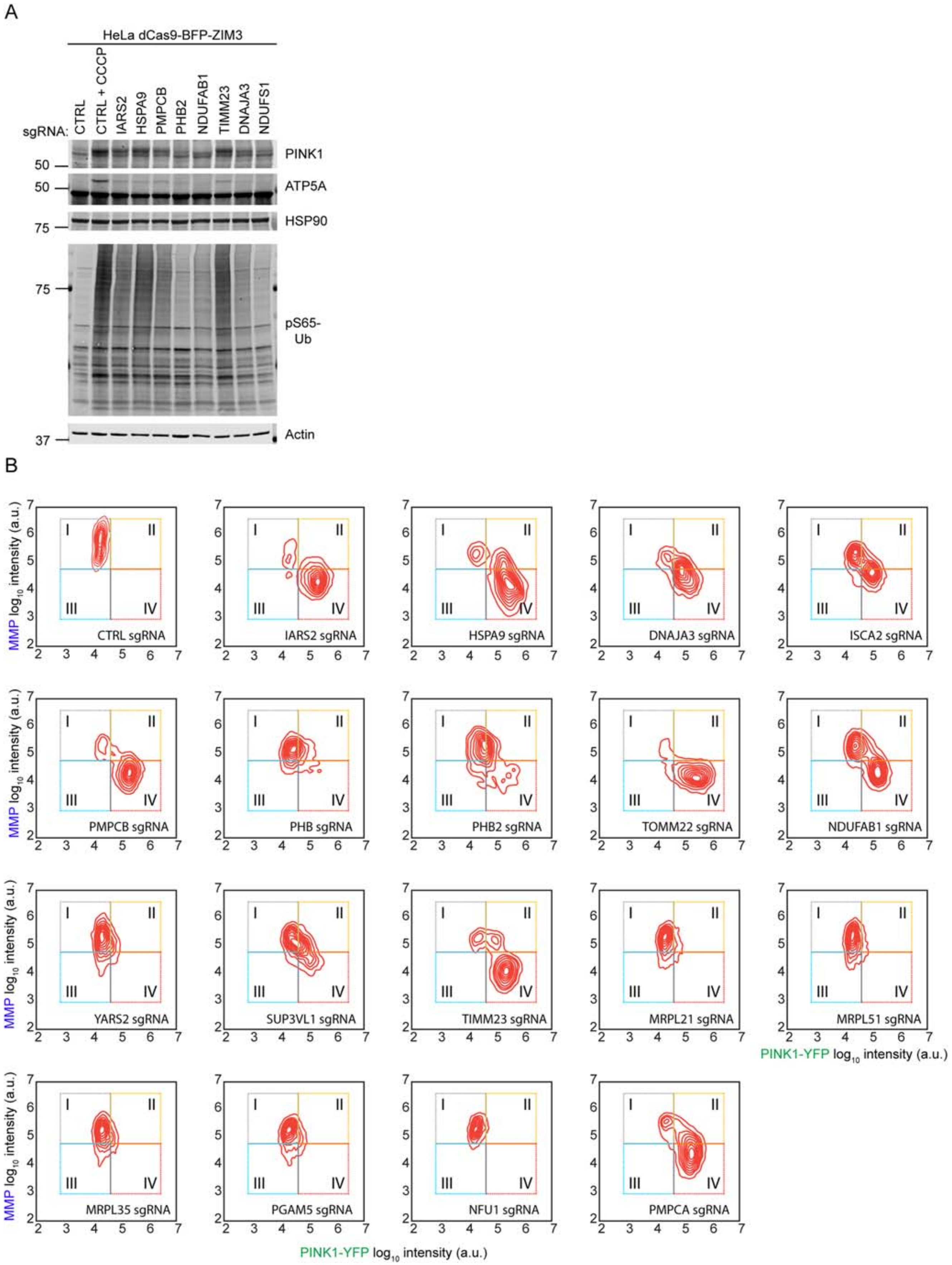
A
HeLa dCas9-BFP-ZIM3
sgRNA:
CTRL
CTRL + CCCP
IARS2
HSPA9
PMPCB
PHB2
NDUFAB1
TIMM23
DNAJA3
NDUFS1
PINK1
ATP5A
HSP90
pS65-Ub
Actin
50
50
75
75
37
B
MMP log10 intensity (a.u.)
PINK1-YFP log10 intensity (a.u.)
I
II
III
IV
CTRL sgRNA
IARS2 sgRNA
HSPA9 sgRNA
DNAJA3 sgRNA
ISCA2 sgRNA
PMPCB sgRNA
PHB sgRNA
PHB2 sgRNA
TOMM22 sgRNA
NDUFAB1 sgRNA
YARS2 sgRNA
SUP3VL1 sgRNA
TIMM23 sgRNA
MRPL21 sgRNA
MRPL51 sgRNA
MRPL35 sgRNA
PGAM5 sgRNA
NFU1 sgRNA
PMPCA sgRNA

© The Author(s)

Figure EV2. Top PINK1–Parkin activators stabilize endogenous PINK1 and lower MMP.

(**A**) Representative immunoblots of HeLa$^{dCas9\text{-}BFP\text{-}ZIM3}$ cells treated with 10 µM CCCP for 4 h or transduced with indicated sgRNAs, illustrating PINK1 stabilization and activation. $N = 3$ independent experiments. (**B**) Representative 2D kernel density plots comparing single-cell PINK1-YFP intensity and intensity of the MMP sensitive dye MitoLite NIR as in (Fig. 4F).

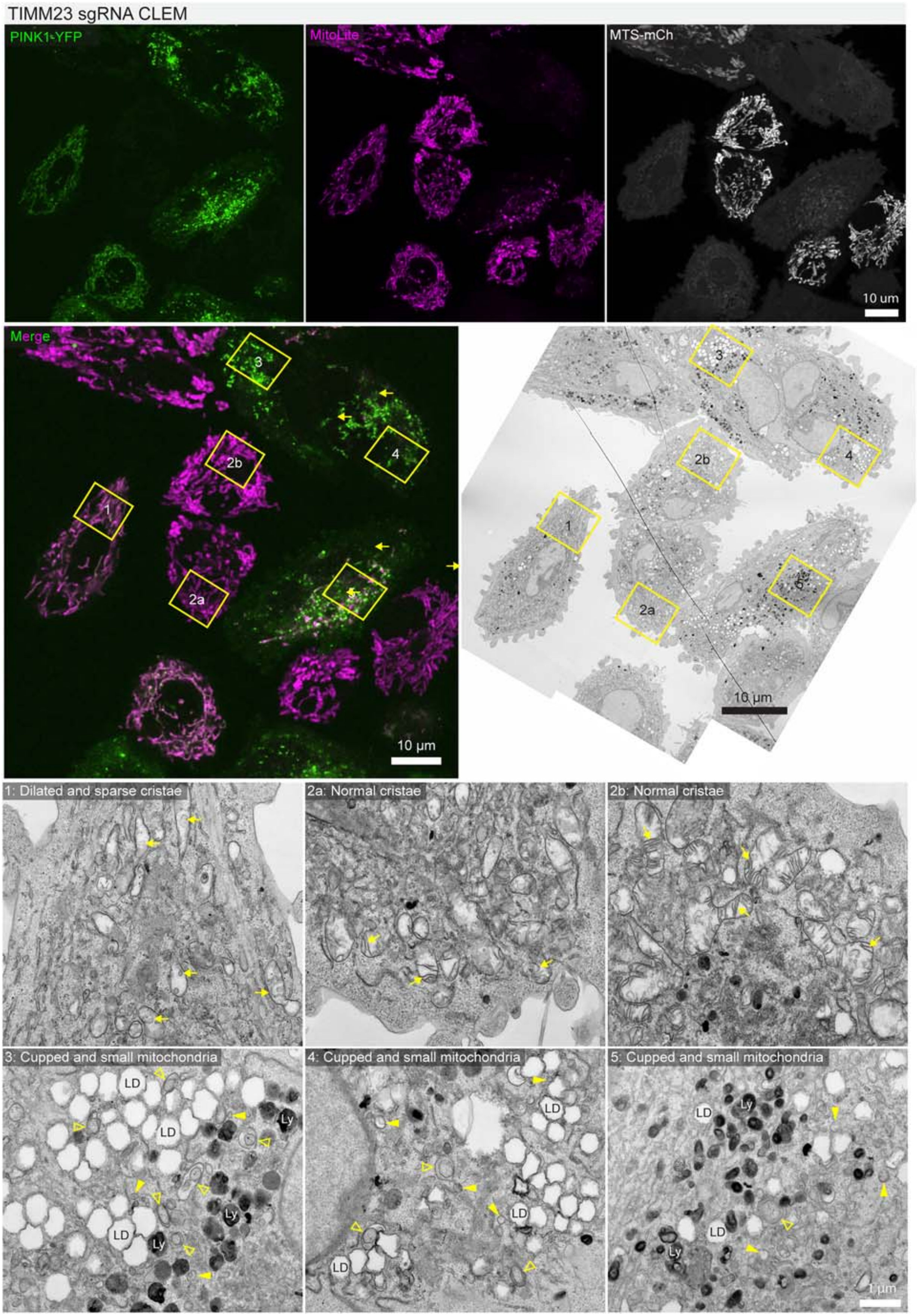
TIMM23 sgRNA CLEM
PINK1-YFP
MitoLite
MTS-mCh
10 um
Merge
3
4
2b
1
2a
5
10 μm
10 μm
1: Dilated and sparse cristae
2a: Normal cristae
2b: Normal cristae
3: Cupped and small mitochondria
4: Cupped and small mitochondria
5: Cupped and small mitochondria
LD
Ly
1 μm

◀ **Figure EV3. CLEM of TIMM23 knockdown cells demonstrates different ultrastructural features of PINK1-targeted mitochondria with and without MMP.**

CLEM images from a TIMM23 KD HeLa$^{PINK1\text{-}YFP+MTS\text{-}mCh}$ cells, illustrating that cells with maintained MMP and PINK1-YFP accumulation did not result in mitochondrial cupping (region 1) while cells where PINK1-YFP accumulated and MMP was lost mitochondrial cupping was observed (regions 3–5), these cells also had lipid droplets and lysosomes around the mitochondria. Live cells were imaged in the presence of MMP dye MitoLite, followed by fixation and EM processing/imaging. Yellow boxes show the corelating cells between the light microscopy and EM. Arrows – cristae feature, empty arrowhead – cupped mito, closed arrowhead – small mito, LD – lipid droplet, and Ly – lysosome. Scale bar = 10 µm on all images but Scale bar = 1 µm on zoomed in regions of TEM (bottom of figure).

© The Author(s)

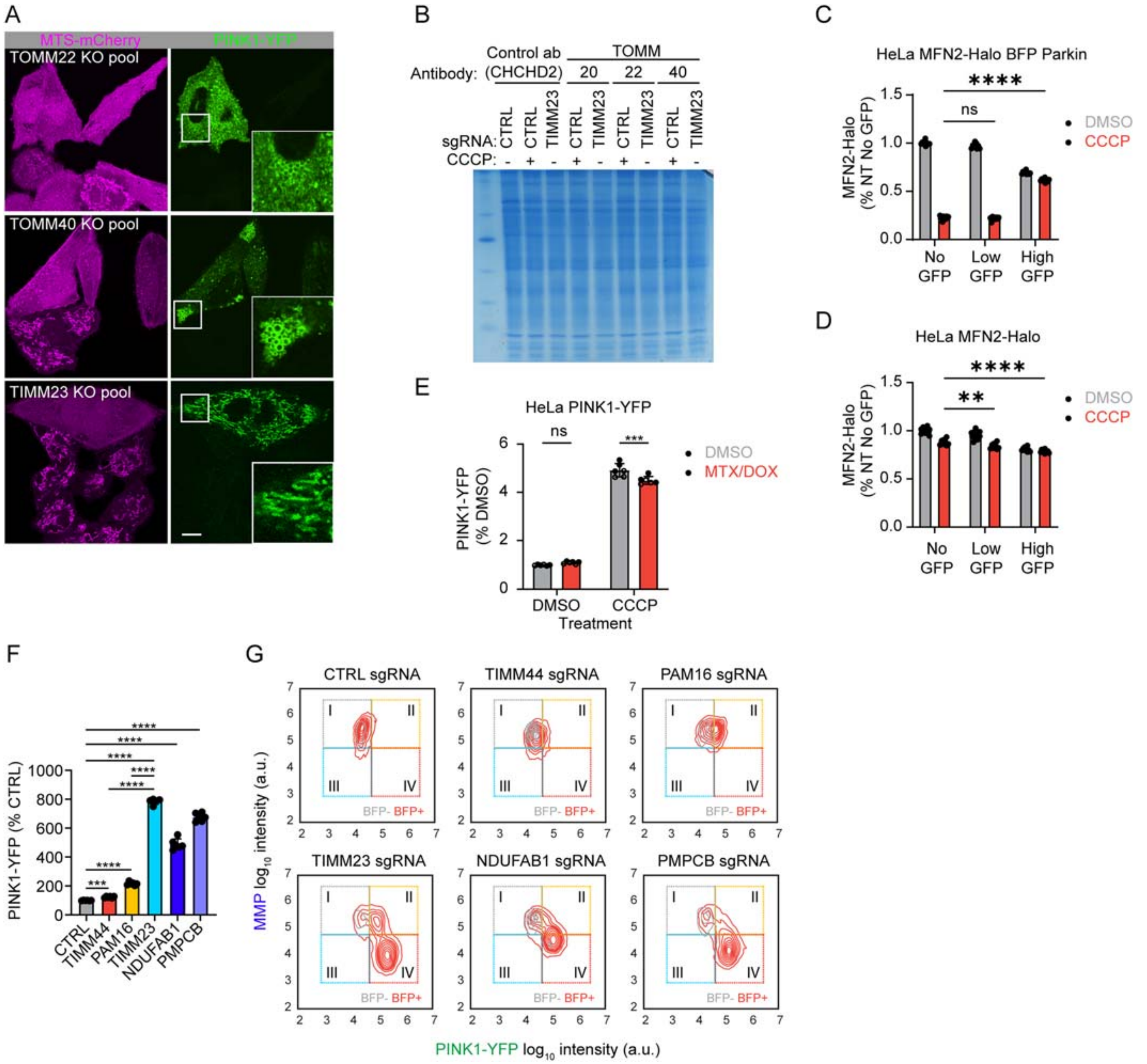

Figure EV4. PINK1 is differentially affected by loss of the TOM complex, TIM23, and the PAM import motor.

(**A**) Representative confocal images of TOMM22, TOMM40, TIMM23 KO pools in HeLa$^{PINK1\text{-}YFP+MTS\text{-}mCh}$ cells showing PINK1-YFP accumulates in the same pattern as observed by CRISPRi. Images were obtained 7 or 8 days after electroporation with Cas9 ribonucleoprotein complexes. Scale bar 10 = µm. (**B**) Total protein measured via SimplyBlue SafeStain of same gel as in (Fig. 5J), demonstrating equal loading. (**C**) Flow cytometry data of HeLa$^{MFN2\text{-}Halo}$ cells + BFP-Parkin transfected with ATP5MG-mCherry-sfGFP and treated +/− 10 µM CCCP for 4 h, illustrating differences in MFN2-Halo levels in the presence of the clogger. ns$P = 0.5976$, ****$P \le 0.0001$ (exact P value $P < 1e\text{-}15$) by two-way ANOVA with Tukey's multiple comparisons test. Error bars mean +/− SD. $N = 3$ independent experiments, 9 replicates. (**D**) Flow cytometry data of HeLa$^{MFN2\text{-}Halo}$ cells transfected with ATP5MG-mCherry-sfGFP and treated +/− 10 µM CCCP for 4 h, illustrating differences in MFN2-Halo levels in the presence of the clogger. **$P = 0.0086$, ****$P \le 0.0001$ (exact P value $P = 7.4e\text{-}09$) by two-way ANOVA with Tukey's multiple comparisons test. Error bars mean +/− SD. $N = 3$ independent experiments, 10 replicates. (**E**) Flow cytometry data of HeLa$^{PINK1\text{-}YFP}$ cells expressing TOMM70 endogenously tagged with HaloTag and IMMT-DHFR clogger. Cells were treated +/− 20 µM CCCP for 4 h, demonstrating differences in PINK1-YFP stabilization. ns$P = 0.4962$, ***$P = 0.0004$ by two-way ANOVA with Šídák's multiple comparisons test. Error bars mean +/− SD. $N = 6$ replicates run on two different occasions (separate occasions denoted by open or closed circles). (**F**) Flow cytometry of HeLa$^{PINK1\text{-}YFP}$ cells. ***$P = 0.0003$, ****$P \le 0.0001$ (exact P values - CTRL vs PAM16, $P = 1.1e\text{-}05$; CTRL vs TIMM23, $P = 9.3e\text{-}07$; CTRL vs NDUFAB1, $P = 9e\text{-}06$; CTRL vs PMPCB, $P = 3.6e\text{-}07$; TIMM44 vs TIMM23, $P = 1.2e\text{-}06$; PAM16 vs TIMM23, 1.3e-08). Error bars mean +/− SD. $N = 6$ replicates (5 for TIMM23) from 2 independent transductions. (**G**) Representative 2D kernel density plots comparing single-cell PINK1-YFP intensity and intensity of the MMP sensitive dye MitoLite NIR as in (Fig. 4F).

 © The Author(s)

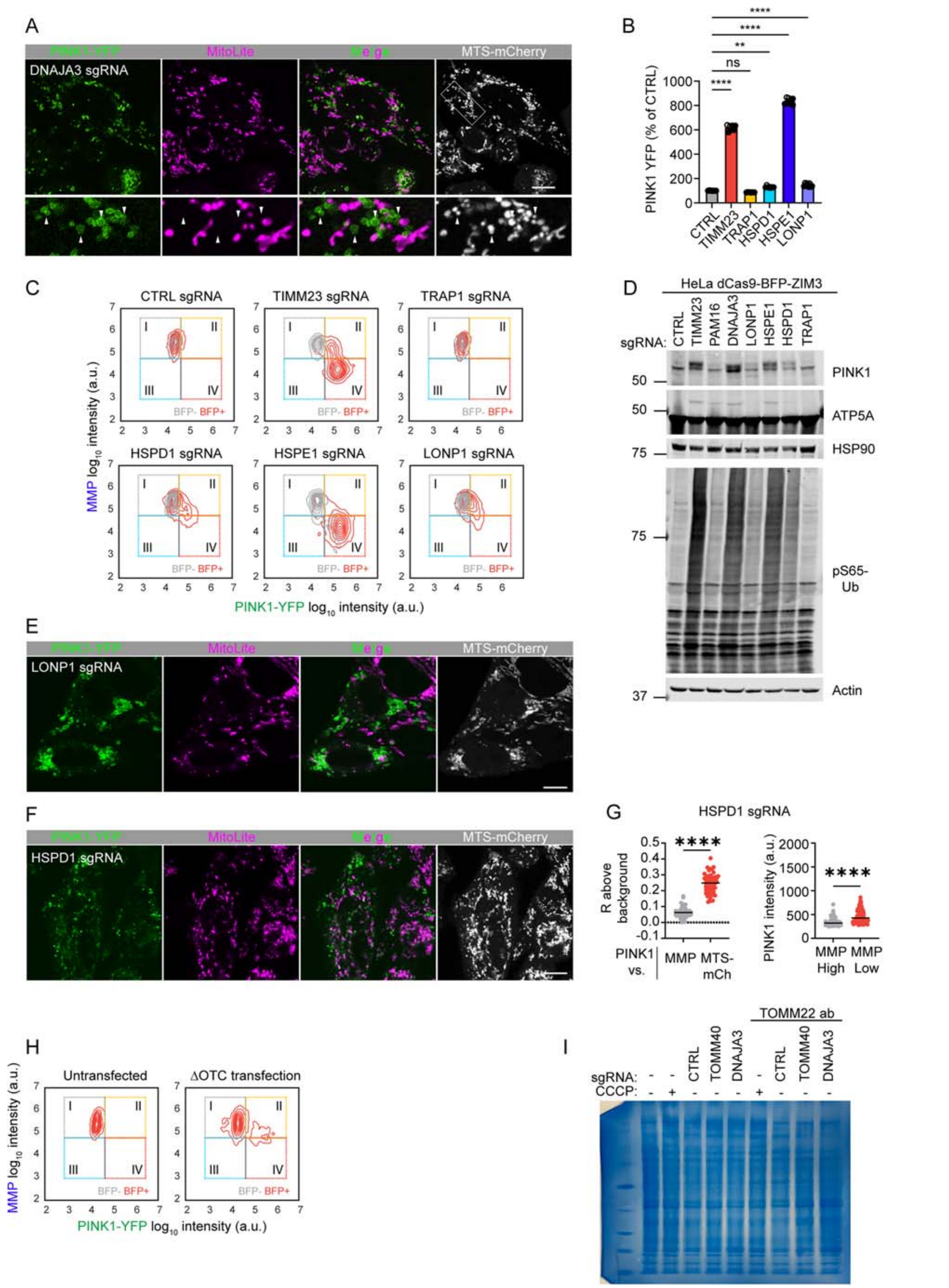
A
PINK1-YFP
MitoLite
Merge
MTS-mCherry
DNAJA3 sgRNA
B
PINK1 YFP (% of CTRL)
CTRL
TIMM23
TRAP1
HSPD1
HSPE1
LONP1
ns
C
CTRL sgRNA
TIMM23 sgRNA
TRAP1 sgRNA
HSPD1 sgRNA
HSPE1 sgRNA
LONP1 sgRNA
BFP- BFP+
MMP log10 intensity (a.u.)
PINK1-YFP log10 intensity (a.u.)
D
HeLa dCas9-BFP-ZIM3
sgRNA:
CTRL
TIMM23
PAM16
DNAJA3
LONP1
HSPE1
HSPD1
TRAP1
PINK1
ATP5A
HSP90
pS65-Ub
Actin
E
LONP1 sgRNA
F
HSPD1 sgRNA
G
HSPD1 sgRNA
R above background
PINK1 vs.
MMP
MTS-mCh
PINK1 intensity (a.u.)
MMP High
MMP Low
H
Untransfected
ΔOTC transfection
I
TOMM22 ab
sgRNA:
CCCP:
TOMM40
DNAJA3

Figure EV5. Protein misfolding in the mitochondrial matrix activates the PINK1–Parkin pathway through its effect on MMP.

(**A**) Representative confocal image of live HeLa$^{PINK1\text{-}YFP+MTS\text{-}mCh}$ cells transduced with a guide targeting DNAJA3. Rectangle images are zoomed in area of white rectangle outlined in square image above. Arrowheads point to cells that have high PINK1-YFP expression on mitochondria that have lost MMP and import is not blocked. Scale bar = 10 µm. (**B**) Flow cytometry of HeLa$^{PINK1\text{-}YFP}$ cells transduce with the indicated sgRNA, illustrating PINK1-YFP levels. ns$P = 0.3001$, **$P = 0.0013$, ****$P \leq 0.0001$ (exact P values - CTRL vs TIMM23, $P <$ 1e-15; CTRL vs HSPE1, p = 1e-15; CTRL vs LONP1, $P =$ 8.1e-07), by ordinary one-way ANOVA with Šídák's multiple comparisons test. Error bars mean +/− SD. $N =$ at least 7 replicates from 2 independent transductions (separate transductions denoted by open or closed circles). (**C**) Representative 2D kernel density plots comparing single-cell PINK1-YFP intensity and intensity of the MMP sensitive dye MitoLite NIR from the same experiment as shown in Fig. EV5B. (**D**) Representative immunoblots of HeLa$^{dCas9\text{-}BFP\text{-}ZIM3}$ cells transduced with indicated sgRNAs, illustrating PINK1 stabilization and activation. $N = 3$ independent experiments. (**E**) Representative confocal image of live HeLa$^{PINK1\text{-}YFP+MTS\text{-}mCh}$ cells transduced with a guide targeting LONP1. PINK1-YFP accumulated preferentially on mitochondria with low MMP. Scale bar = 10 µm. (**F**) Representative confocal image of live HeLa$^{PINK1\text{-}YFP+MTS\text{-}mCh}$ cells transduced with a guide targeting HSPD1. PINK1-YFP accumulated preferentially on mitochondria with low MMP. Scale bar = 10 µm. (**G**) Left graph quantification of cells in (Fig. EV5F) was performed as in (Fig. 3G). ****$P \leq 0.0001$ (exact P value $P <$ 1e-15) by two-tailed Mann–Whiteney test. $N = 56$ cells from 4 wells and 2 separate transductions. Right graph quantification of cells in (Fig. EV5F) was performed as in (Fig. 3F). ****$P \leq 0.0001$ (exact P value $P <$ 1e-15) by Wilcoxon matched-pairs signed rank test. $N = 56$ cells from 4 wells and 2 separate transductions. (**H**) Representative 2D kernel density plots comparing single-cell PINK1-YFP intensity and intensity of the MMP sensitive dye MitoLite NIR in HeLa$^{PINK1\text{-}YFP+TOMM70\text{-}Halo}$ cells +/− transient transfection of ΔOTC. (**I**) Total protein measured via SimplyBlue SafeStain of same gel in (Fig. 7D), demonstrating equal loading.

 © The Author(s)
